# Both Alpha- and Beta-Rhizobia Occupy the Root Nodules of *Vachellia karroo* in South Africa

**DOI:** 10.3389/fmicb.2019.01195

**Published:** 2019-06-04

**Authors:** Chrizelle W. Beukes, Francois S. Boshoff, Francina L. Phalane, Ahmed I. Hassen, Marianne M. le Roux, Tomasz Stȩpkowski, Stephanus N. Venter, Emma T. Steenkamp

**Affiliations:** ^1^Department of Biochemistry, Genetics and Microbiology, Forestry and Agricultural Biotechnology Institute, University of Pretoria, Pretoria, South Africa; ^2^Agricultural Research Council, Plant Health and Protection Institute, Pretoria, South Africa; ^3^South African National Biodiversity Institute, Pretoria National Botanical Garden, Pretoria, South Africa; ^4^Department of Botany and Plant Biotechnology, University of Johannesburg, Johannesburg, South Africa; ^5^Autonomous Department of Microbial Biology, Faculty of Agriculture and Biology, Warsaw University of Life Sciences – SGGW, Warsaw, Poland

**Keywords:** alpha-rhizobia, beta-rhizobia, *Acacia karroo*, *Vachellia karroo*, *Paraburkholderia*, *Bradyrhizobium*, South Africa

## Abstract

*Vachellia karroo* (formerly *Acacia karroo*) is a wide-spread legume species indigenous to southern Africa. Little is known regarding the identity or diversity of rhizobia that associate with this plant in its native range in South Africa. The aims of this study were therefore: (i) to gather a collection of rhizobia associated with *V. karroo* from a wide range of geographic locations and biomes; (ii) to identify the isolates and infer their evolutionary relationships with known rhizobia; (iii) to confirm their nodulation abilities by using them in inoculation assays to induce nodules under glasshouse conditions. To achieve these aims, soil samples were collected from 28 locations in seven biomes throughout South Africa, which were then used to grow *V. karroo* seedlings under nitrogen-free conditions. The resulting 88 bacterial isolates were identified to genus-level using 16S rRNA sequence analysis and to putative species-level using *recA*-based phylogenetic analyses. Our results showed that the rhizobial isolates represented members of several genera of Alphaproteobacteria (*Bradyrhizobium*, *Ensifer*, *Mesorhizobium*, and *Rhizobium*), as well as *Paraburkholderia* from the Betaproteobacteria. Our study therefore greatly increases the known number of *Paraburkholderia* isolates which can associate with this southern African mimosoid host. We also show for the first time that members of this genus can associate with legumes, not only in the Fynbos biome, but also in the Albany Thicket and Succulent Karoo biomes. Twenty-six putative species were delineated among the 88 isolates, many of which appeared to be new to Science with other likely being conspecific or closely related to *E. alkalisoli*, *M. abyssinicae*, *M. shonense*, and *P. tropica*. We encountered only a single isolate of *Bradyrhizobium*, which is in contrast to the dominant association of this genus with Australian *Acacia*. *V. karroo* also associates with diverse genera in the Grassland biome where it is quite invasive and involved in bush encroachment. Our findings therefore suggest that *V. karroo* is a promiscuous host capable of forming effective nodules with both alpha- and beta-rhizobia, which could be a driving force behind the ecological success of this tree species.

## Introduction

*Vachellia karroo* (formerly *Acacia karroo*) (Banfi and Galasso, 2008; Kyalangalilwa et al., 2013) is a legume tree species belonging to the mimosoid clade, which is an informal group nested within the newly recircumscribed Caesalpinioideae subfamily (LPWG, 2017). *Vachellia* contains 161 species and has a pantropical distribution, although 73 species can be found in Africa and Madagascar (Lewis, 2005; Kyalangalilwa et al., 2013). This is a relatively small genus when compared to the Australian endemic genus *Acacia* sensu stricto, which contains 1021 species (Kyalangalilwa et al., 2013). *V. karroo* is commonly referred to as “sweet thorn” and has significant economic importance (Hayward, 2004). It has been shown to have medicinal properties as its leaves and bark can be used to treat diarrhoea, while an exudate from the tree can be used as an emollient for conjunctivitis and parts of the tree also have broad-spectrum anti-microbial activity (van Wyk, 2011; Cock and van Vuuren, 2015; Maroyi, 2017). The leaves, pods and fruits have the potential to be used as fodder for ruminant livestock (Gxasheka et al., 2015; Brown et al., 2016; Dingaan and du Preez, 2018), while its flowers are important for honey production (Dingaan and du Preez, 2018). With regards to human consumption, its roasted seeds can be used as a coffee substitute and its gum in the production of sweets (Cock and van Vuuren, 2015).

Among tree species, *V. karroo* has the widest distribution in South Africa (Dingaan and du Preez, 2018), extending from the Southwestern Cape northwards into Namibia, Botswana, Zambia, Angola and Zimbabwe (Taylor and Barker, 2012). The species can grow in different soils (ranging from sand to heavy clays), in relatively wet to arid areas (200–1500 mm annual rainfall) and from sea level to an elevation of 1800 m (Maroyi, 2017). As a pioneer, *V. karroo* inhabits various biomes and has the ability to grow and thrive in a range of climatic and edaphic conditions (Brain et al., 1997; Taylor and Barker, 2012), where it is resistant to factors such as salinity, drought, fire and frost (Brain et al., 1997). This is thought to contribute significantly to its invasiveness, because *V. karroo* is the foremost woody invader in South African grasslands (O’Connor, 1995; Taylor and Barker, 2012), a phenomenon referred to as “bush encroachment” (Dingaan and du Preez, 2018).

As with most other legumes, *V. karroo* can form nitrogen-fixing root nodules (Barnes, 2001) in mutualistic symbioses with specific soil bacteria referred to as rhizobia (Poole et al., 2018). These bacteria do not form a monophyletic group and are represented by species in 18 genera of the Alpha- and Betaproteobacteria (Lee and Hirsch, 2006; Poole et al., 2018; de Lajudie et al., 2019). To differentiate between rhizobia from these two classes, they are informally referred to as alpha- and beta-rhizobia (Moulin et al., 2001). Of these two groups, the beta-rhizobia was discovered more recently (Moulin et al., 2001), with one of the first recognised beta-rhizobial species, *Paraburkholderia tuberum* (formerly *Burkholderia tuberum*; Sawana et al., 2014), reported as a symbiont of the indigenous South African papilionoid legume, *Aspalathus carnosa* (Lemaire et al., 2016a), while also being able to nodulate several *Cyclopia* species and siratro (*Macroptilium atropurpureum*; Elliott et al., 2007a). Numerous nodulating *Paraburkholderia* species have since been reported from South Africa, particularly from the Cape Floristic Region in the Western Cape Province (de Meyer et al., 2013a,b, 2014; Steenkamp et al., 2015), and all in association with papilionoid legumes. This is in contrast to other parts of the world, especially South America, where *Paraburkholderia* is primarily associated with mimosoid legumes (Chen et al., 2005, 2007; Bontemps et al., 2010; Mishra et al., 2012; Bournaud et al., 2013). Various studies thus suggest that South Africa and South America represent distinct centres of diversity for the beta-rhizobial members of this genus (Gyaneshwar et al., 2011; Beukes et al., 2013; Lemaire et al., 2015a; Silva et al., 2018).

Very little information is available regarding the identity and diversity of the rhizobial partners of *V. karroo*, especially from South Africa and areas where *V. karroo* occurs naturally (Ngwenya et al., 2016). The only previous study that has focused on *V. karroo* rhizobia in South Africa showed it is nodulated by diverse symbionts, representing “fast- and slow-growers,” (Ngwenya et al., 2016), which is similar to what has been found in an earlier study from Libya (Mohamed et al., 2000). The terms “fast- and slow-growing rhizobia” typically refer to the alpha-rhizobial genera *Rhizobium* and *Bradyrhizobium*, respectively (Leary et al., 2006). The first DNA-based study to attempt identification of the rhizobial symbionts of *V. karroo* was performed in Kenya with a portion of the 16S ribosomal RNA (rRNA) gene to diagnose an isolate of *Rhizobium* (McInroy et al., 1999). A more recent study from Algeria employed full-length 16S rRNA gene sequences to identify *V. karroo* symbionts as members of the alpha-rhizobial genera *Rhizobium* and *Ensifer* (Boukhatem et al., 2012). Furthermore, a number of studies on the rhizobia of *Acacia* sensu lato (excluding *V. karroo*) in the northern, eastern and western parts of Africa have shown that these isolates represent members of the alpha-rhizobial genera *Bradyrhizobium*, *Ensifer*, *Mesorhizobium*, and *Rhizobium* (Khbaya et al., 1998; McInroy et al., 1999; Mohamed et al., 2000; Ba et al., 2002; Odee et al., 2002; Wolde-meskel et al., 2004, 2005; Sarr et al., 2005; Romdhane et al., 2006; Diouf et al., 2007; Degefu et al., 2011; Abdi et al., 2012).

The mutualistic interaction between rhizobia and legumes could be an important factor when the plant is introduced into a new environment (Stanton-Geddes and Anderson, 2011; Klock et al., 2015; La Pierre et al., 2017). The symbiosis is especially important to the establishment, growth and survival of the legume when soils are nutrient-poor (Thrall et al., 2000, 2005; Klock et al., 2015). Knowledge regarding the diversity and identity of the rhizobial symbionts of *V. karroo* would therefore be crucial for understanding their role in the ecological success of this legume. To address this knowledge gap, our overall goal was to determine the identity of the rhizobial symbionts of *V. karroo* growing in diverse biomes across South Africa. The aims of the study were: (i) to assemble a collection of rhizobial isolates associated with *V. karroo* from a broad range of geographic locations and biomes, and (ii) to determine the identity of the rhizobia using DNA sequence information from the widely used 16S rRNA and *recA* genes. How strict or broad the range of possible rhizobial partners of *V. karroo* is could provide important information on the potential of this legume to find a suitable rhizobial partner in a particular environment.

## Materials and Methods

### Isolate Collection and Nodulation Confirmation

**FIGURE 1:**
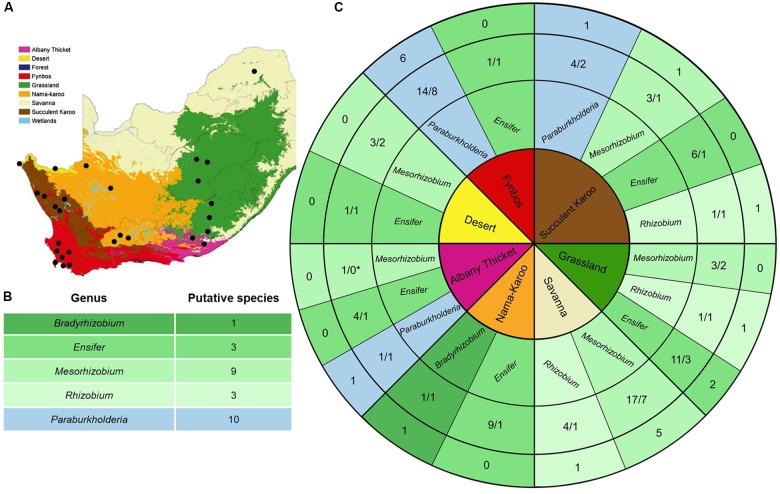
**(A)** Biome map of South Africa, Lesotho and Swaziland (as in Driver et al., 2005; from the national Spatial Biodiversity Assessment of 2004, courtesy of SANBI). The nine biomes are coloured according to the key and black dots indicate the sampling localities. **(B)** Summary of the putative species encountered in this study. **(C)** The core of this diagram lists the seven sampled biomes (colour-coded as in **A**). The second track indicates the genera isolated from each biome (with alpha-rhizobia appearing in shades of green while beta-rhizobia appear in blue), while the third track shows the number of isolates sampled from each genus for that specific biome relative to the number of *recA* phylogeny-based putative species they formed part of. The outer track shows the number of unique *recA* phylogeny-based putative species recovered for a specific biome for each rhizobial genus. We were unable to amplify the *recA* gene sequence for the *Mesorhizobium* isolate recovered from the Albany Thicket which is why it is indicated with an asterisk, as it could not be assigned to a species.

To collect rhizobial isolates associated with *V. karroo* from a wide geographic area in South Africa, we used a “trapping” approach. In this approach, soil close to the roots of *V. karroo* (i.e., rhizosphere soil) was collected and used for planting and growing the germinated seeds of the host plant under nitrogen-free conditions. Such an environment forces the seedlings to interact with the rhizobia in the collected soil and form nodules to obtain nitrogen for growth. To this end, we collected soil from 28 locations across six of South Africa’s nine provinces and included seven biomes [biome definitions *sensu* Mucina and Rutherford (2006); Table 1 and Figure 1A]. Seedlings of *V. karroo* were grown from seed that were first scarified with concentrated sulphuric acid for 2 h, followed by five rinses in sterile distilled water (sH_2_O) and a further three-hour imbibement in sH_2_O. The swollen seeds (indicative of a damaged seed coat through which water could be absorbed) were plated onto 1.5% water agar (w/v) plates and incubated at 28°C in the dark for 2 days for germination to occur.

**Table 1:** Information regarding the rhizobia associated with *Vachellia karroo* included in this study.

Biome^a^	Province^b^	Location	Isolate^c^	Nodulation Status^d^	Genus Placement^e^	Putative species^f^ (=conspecific^g^)
Albany Thicket	EC	Grahamstown	8A (LN890724)	nd	*Ensifer*	VK-2
			8B (LN890725)	nd	*Ensifer*	VK-2
	EC	Jansenville	7A (LN890722)	nd	*Ensifer*	VK-2
			7B (LN890723)	nd	*Ensifer*	VK-2
			7D (LN890677)	nd	*Mesorhizobium*	ND
			7E (LN890699)	nd	***Paraburkholderia***	VK-16
Desert	NC	Pofadder	4A (LN890675)	nd	*Mesorhizobium*	VK-4 (*M. abyssinicae*)
			4B (LN890678)	nn	*Mesorhizobium*	VK-4 (*M. abyssinicae*)
			4C (LN890665)	nd	*Mesorhizobium*	VK-6 (*M. shonense*)
		Alexander Bay	13E (LN890731)	nn	*Ensifer*	VK-2
Fynbos	WC	Robertson	14C (LN890732)	nd	*Ensifer*	VK-2
		Cape Town (Steenberg plateau)	1 (LN890711)	C	***Paraburkholderia***	VK-17
			1.1 (LN890709)	nd	***Paraburkholderia***	VK-15
			1.2 (LN890708)	nd	***Paraburkholderia***	VK-15
			32 (LN890715)	nd	***Paraburkholderia***	VK-18
		Hermanus (Vogelgat Nature Reserve)	11 (LN890707)	nd	***Paraburkholderia***	VK-14
			11.1 (LN890714)	S, C	***Paraburkholderia***	VK-21
			11.2 (LN890710)	nd	***Paraburkholderia***	VK-15
			31.1 (LN890717)	nd	***Paraburkholderia***	VK-20
		Tulbagh (Tulbagh/Winterhoek road)	16.2 (LN890703)	nn	***Paraburkholderia***	VK-13
			29 (LN890712)	C	***Paraburkholderia***	VK-22 (*P. tropica*)
		Stellenbosch (Jonkershoek Nature Reserve)	40 (LN890713)	S	***Paraburkholderia***	VK-13
		False Bay (Cape Hangklip)	22.2 (LN890700)	S	***Paraburkholderia***	VK-13
		Rosendal farm	21 (LN890705)	S	***Paraburkholderia***	VK-13
		Gydo Pass	43 (LN890702)	nd	***Paraburkholderia***	VK-20
Grassland	EC	Stutterheim	9A (LN890719)	nn	***Burkholderia***	ND
			9B (LN890688)	nd	*Mesorhizobium*	VK-7
			9C (LN890718)	nn	***Burkholderia***	ND
			9D (LN890687)	nd	*Mesorhizobium*	VK-7
		Aliwal North	11A (LN890697)	nn	*Agrobacterium*	ND
			11D (LN890729)	nd	*Ensifer*	VK-2
		Queenstown	18A (LN890741)	nd	*Ensifer*	VK-2
			18C (LN890742)	S	*Ensifer*	VK-2
			18D (LN890743)	nd	*Ensifer*	VK-2
			18E (LN890744)	nd	*Ensifer*	VK-2
	FS	Vredefort	1B (LN890691)	C	*Rhizobium*	VK-25
			1C (LN890721)	nd	*Ensifer*	VK-2
			1D (LN890676)	nd	*Mesorhizobium*	VK-4 (*M. abyssinicae*)
			1E (LN890751)	S	*Ensifer*	VK-3
		Reddersburg	5A (LN890749)	nn	*Ensifer*	VK-1
			5B (LN890747)	C	*Ensifer*	VK-1
			5C (LN890748)	nd	*Ensifer*	VK-1
			5D (LN890750)	nd	*Ensifer*	VK-1
Nama-Karoo	EC	Graaff-Reinet	10A (LN890726)	nd	*Ensifer*	VK-2
			10C (LN890727)	nd	*Ensifer*	VK-2
			10E (LN890728)	nd	*Ensifer*	VK-2
	NC	Augrabies Nature Reserve	12A (LN890730)	nd	*Ensifer*	VK-2
		Prieska	19A (LN890745)	nd	*Ensifer*	VK-2
	WC	Beaufort West	15A (LN890733)	S	*Ensifer*	VK-2
			15B (LN890698)	nd	*Bradyrhizobium*	VK-26
			15C (LN890734)	nd	*Ensifer*	VK-2
			15D (LN890735)	nd	*Ensifer*	VK-2
			15E (LN890736)	nd	*Ensifer*	VK-2
Savanna	LP	Mookgophong	20B (LN890695)	S, C	*Rhizobium*	VK-24
			20C (LN890694)	S, C	*Rhizobium*	VK-24
			21A (LN890693)	S	*Rhizobium*	VK-24
			21B (LN890692)	S, C	*Rhizobium*	VK-24
			21C (LN890666)	S	*Mesorhizobium*	VK-6 (*M. shonense*)
			21E (LN890667)	nd	*Mesorhizobium*	VK-6 (*M. shonense*)
			22B (LN890668)	C	*Mesorhizobium*	VK-6 (*M. shonense*)
			22C (LN890669)	nd	*Mesorhizobium*	VK-6 (*M. shonense*)
			22E (LN890670)	nd	*Mesorhizobium*	VK-6 (*M. shonense*)
			23A (LN890680)	nn	*Mesorhizobium*	VK-11
			23B (LN890683)	nd	*Mesorhizobium*	VK-10
			23D (LN890679)	nd	*Mesorhizobium*	VK-11
			23E (LN890671)	nd	*Mesorhizobium*	VK-6 (*M. shonense*)
			24A (LN890682)	nn	*Mesorhizobium*	VK-12
			24D (LN890681)	C	*Mesorhizobium*	VK-12
			25A (LN890674)	nn	*Mesorhizobium*	VK-8
			25B (LN890673)	nd	*Mesorhizobium*	VK-8
			25D (LN890672)	nn	*Mesorhizobium*	VK-5
	NW	Bloemhof	3C (LN890664)	nd	*Mesorhizobium*	VK-6 (*M. shonense*)
			3D (LN890690)	C	*Mesorhizobium*	VK-7
			3E (LN890689)	nd	*Mesorhizobium*	VK-7
Succulent Karoo	NC	Hondeklip Bay	16A (LN890737)	nd	*Ensifer*	VK-2
			16B (LN890738)	nd	*Ensifer*	VK-2
			16D (LN890739)	nd	*Ensifer*	VK-2
			16E (LN890740)	nd	*Ensifer*	VK-2
		Kamieskroon (Grootberg)	35.1 (LN890716)	S, C	***Paraburkholderia***	VK-19
		Kamieskroon-Garies	44.1 (LN890704)	nd	***Paraburkholderia***	VK-13
	WC	Calitzdorp	6A (LN890746)	S, C	*Ensifer*	VK-2
			6B (LN890696)	nd	*Rhizobium*	VK-23
			6D (LN890720)	nd	*Ensifer*	VK-2
		Van Rhyns Pass	2B (LN890685)	nd	*Mesorhizobium*	VK-9
			2C (LN890684)	nd	*Mesorhizobium*	VK-9
			2D (LN890686)	nd	*Mesorhizobium*	VK-9
			3.1 (LN890706)	S, C	***Paraburkholderia***	VK-13
			9.1 (LN890701)	nd	***Paraburkholderia***	VK-13

*^a^Biomes are demarcated as defined by Mucina and Rutherford (2006) and locations were allocated to biomes per the 2012 National Vegetation Map (bgis.sanbi.org/MapViewer). ^b^Abbreviations used for the provinces are as follows: EC = Eastern Cape, FS = Free State, LP = Limpopo, NC = Northern Cape, NW = North West and WC = Western Cape. ^c^The isolate name is followed by the 16S rRNA accession numbers as assigned by the European Nucleotide Archive (https://www.ebi.ac.uk/ena). Accession numbers as assigned to the recA sequences are listed on the individual recA phylogenies. ^d^Nodulation test results as determined with the hosts siratro (Macroptilium atropurpureum) and cowpea (Vigna unguiculata). “nn” – indicates that no nodulation occurred, “nd” – indicates that successful nodulation by this isolate has not yet been proved, while “S” and “C” refers to the positive results (i.e., the formation of nodules) on either siratro or cowpea. ^e^Genus identity of the isolate according to 16S rRNA gene sequence comparisons with the GenBank database. Betaproteobacteria appear in bold. ^f^Delineation of the isolates into putative species was according to Nixon and Wheeler (1990) and based upon bootstrap-supported diagnosable groups formed in the recA phylogeny. ND = Not determined; Mesorhizobium isolate 7D does not have a recA sequence and is therefore not assigned to a lineage. ^g^The described species with which our putative species shares the closest evolutionary history and to which these V. karroo isolates might belong when more phylogenetic analyses are performed.*

The “trapping” experiments were performed in a glasshouse where the seedlings were planted in Leonard jars (Howieson and Dilworth, 2016). Three seedlings were planted in each Leonard jar. The sterile sand in the container was then covered with 20 g of the soil collected from a specific location, thereby filling up the holes in which the seedlings were planted (i.e., seedlings were thus only provided with the nitrogen potentially contained within the 20 g of soil). Five biological replicates were performed for each sampling location and there was also a control jar to which no sampled soil was added. The growth conditions in the glasshouse was set to a day/night cycle of 14 and 10 h with temperatures from 27 to 28 and 15°C, respectively. The plants were checked for nodule formation 4 months after planting.

*Vachellia karroo* nodules resulting from “trapping” in Western Cape soils were obtained from Dr. M. Maistry (Research leader Dr. S. Chimphango, Department of Biological Sciences, University of Cape Town). The following protocol was used: silica sand was chemically washed with 0.1% (v/v) hydrochloric acid and then rinsed six times with sH_2_O until a neutral pH was achieved. Scarified *V. karroo* seeds were then mixed with the treated silica sand and transferred into pots (10 cm in diameter) containing the collected Western Cape soils. Seedlings were watered until they emerged and then treated twice a week with nitrogen-free Hoagland solution (Howieson and Dilworth, 2016) until the plants were harvested after 4 months.

Root nodules that developed on *V. karroo* plants were surface sterilised (15 min in 3.5% (w/v) sodium hypochlorite), rinsed five times with sH_2_O and then crushed onto Yeast Mannitol Agar (YMA; Howieson and Dilworth, 2016) to obtain pure single colonies. To prove the nodulation ability of the pure isolates, glasshouse trials were performed with the promiscuous plants (i.e. able to interact with many rhizobial partners) siratro (*Macroptilium atropurpureum*) and cowpea (*Vigna unguiculata*). The siratro and cowpea seeds were germinated according to Beukes et al. (2013). Following this procedure, pure cultures of the original “trapped” rhizobia were used as inoculum for the seedlings under nitrogen-free conditions in sterile sand. The resulting nodules were treated as mentioned above and from the pure cultures, 16S rRNA amplification and sequencing were performed (as described below). These sequences were then compared with those of the original “trapping” isolates to confirm their identities. A nodule from each plant (where possible) was also cut open to view the interior, which if pink in colour is due to the presence of leghaemoglobin (an oxygen-carrying protein essential for nitrogen-fixation). The nodules were therefore assumed to be actively fixing nitrogen and were then regarded as effective nodules.

### DNA Extraction, PCR and Sequencing

To determine the identity and diversity of the rhizobial symbionts from the different locations, 16S rRNA and *recA* gene sequences were used. For this purpose, pure cultures of each isolate were grown in 5 ml Yeast Mannitol broth at 28°C with shaking (ca. 130 rpm) for 7 days. Centrifugation for 5 min at 3 824 *rcf* was used to concentrate the bacterial cells into a pellet. Thereafter DNA was extracted using the protocol of Aljanabi and Martinez (1997).

For amplification of a *ca*. 1490 bp fragment of the 16S rRNA gene, the primers 16S-F (5′ AGA GTT TGA TCC TGG CTC AG 3′) (Suau et al., 1999) and 16S-R (5′ TAC CTT GTT ACG ACT TCA CCC CA 3′) (Logan et al., 2000) were used. Every 50 μl PCR reaction consisted of 10 μM of each primer, 0.1 U/μL Super-Therm *Taq* DNA polymerase with reaction buffer (Southern Cross Biotechnology, Cape Town, SA), 25 mM MgCl_2_, 2.5 mM of each dNTP and 50 to 100 ng genomic DNA. The PCR cycling conditions were: initial denaturation of 94°C for 2 min, followed by 35 cycles of denaturation (94°C for 1 min), annealing (60°C for 1 min) and elongation (72°C for 1 min), with a final elongation step at 72°C for 7 min. The resulting amplicons were cleaned using Centri-Sep^TM^ Spin Columns (Life Technologies, CA, United States). The purified amplicons were then subjected to Sanger sequencing, with the same set of primers and the ABI PRISM BigDye Terminator v3.0 Cycle Sequencing Kit on a ABI 3100 Automated Capillary DNA sequencer (Applied Biosystems, CA, United States).

Individual chromatograms were analysed with Chromas Lite v2.01 (Technelysium, Queensland, Australia), before consensus sequences were generated with BioEdit v7.053 (Hall, 1999). By using *blastn* (Altschul et al., 1990), these nearly complete 16S rRNA sequences were compared to those in GenBank (National Centre for Biotechnology Information http://www.ncbi.nlm.nih.gov/; Benson et al., 2017). The latter allowed identification of the isolates to genus-level.

For all rhizobial isolates, a portion of the *recA* gene was then sequenced. For all the alpha-rhizobial isolates, a *ca*. 600 bp region of *recA* was amplified with the primer pair recA-41F (5′ TTC GGC AAG GGM TCG RTS ATG 3′) and recA-640R (5′ ACA TSA CRC CGA TCT TCA TCG 3′) (Vinuesa et al., 2005), while a *ca*. 850 bp region for the beta-rhizobia were amplified with the primers BUR1 (5′ GAT CGA RAA GCA GTT CGG CAA 3′) and BUR2 (5′ TTG TCC TTG CCC TGR CCG AT 3′) (Payne et al., 2005). Amplification and sequencing followed the same procedure as for the 16S rRNA gene, except that the annealing temperature of the PCR was 62°C. The resulting *recA* sequences were analysed with ChromasLite and BioEdit as described above.

### Sequence Alignments and Phylogenetic Analyses

After determining the genus to which each isolate belonged, *recA* nucleotide sequence datasets were constructed by adding the sequence for each described species in the specific genus (as listed on the List of Prokaryotic Names with Standing in Nomenclature (LPSN); www.bacterio.net; Euzéby, 1997; Parte, 2013). We also searched literature and added species which have not yet been included on LPSN (still awaiting validation), as well as any undescribed isolates (which do not yet have species names assigned), originating in Africa or which associates with hosts in *Acacia* sensu lato (see Supplementary Tables S1–S5 for accession numbers and associated references; also for the accessions of the focal isolates). The multiple sequence datasets were manually aligned in BioEdit by making use of the inferred amino acid sequences.

The aligned datasets were then used to create haplotype files with the software DnaSP v5 (Librado and Rozas, 2009). These files only contained unique taxa (i.e. isolates with identical sequences were collapsed into one haplotype and represented by a single sequence). Parameters for the best-fit evolutionary model of each dataset were estimated with jModelTest v2.1.7 (Guindon and Gascuel, 2003; Darriba et al., 2012). These datasets were subjected to maximum-likelihood phylogenetic analysis using the best-fit model in PhyML v3.1 (Guindon and Gascuel, 2003). Branch support was estimated using the same approach and 1000 pseudoreplicates (Felsenstein, 1985). The initial number of taxa included, the resulting number of haplotypes, the length of the alignment and best-fit evolutionary model for each dataset are summarised in Supplementary Table S6. For each dataset, a group of three isolates from different, but closely related genera, were included for outgroup purposes. The resulting phylogenies were edited in MEGA6 (Tamura et al., 2013), with the final trees showing all the isolates constituting a haplotype, as well as information regarding the country of origin and host for each isolate.

## Results

The “trapping” experiments resulted in the isolation of 88 bacterial strains (Table 1). Comparison of their 16S rRNA sequences to those in GenBank revealed that they represented seven genera (Table 1). According to these *blastn* results, 67 of the isolates represented Alphaproteobacteria and formed part of the genera *Ensifer* (32 isolates), *Mesorhizobium* (27 isolates), *Rhizobium* (six isolates), *Bradyrhizobium* (one isolate) and *Agrobacterium* (one isolate). The remaining 21 isolates represented Betaproteobacteria and formed part of the genera *Paraburkholderia* (19 isolates) and *Burkholderia* (two isolates).

Isolates identified as belonging to the genera *Agrobacterium*, *Bradyrhizobium* and *Burkholderia* did not form nodules (Table 1), during nodulation tests. Four isolates for *Mesorhizobium*, eight for *Paraburkholderia* and five each for *Ensifer* and *Rhizobium* did, however, induce effective nodules on both or one of these legumes. Those that induced nodules in both siratro and cowpea were as follows: *Paraburkholderia* spp. 3.1, 11.1, and 35.1; *Ensifer* sp. 6A and lastly *Rhizobium* spp. 20B, 20C, and 21B. Those that nodulated only cowpea were: *Ensifer* sp. 5B, *Mesorhizobium* spp. 3D, 22B, 24D; *Rhizobium* sp. 1B and *Paraburkholderia* spp. 1 and 29. Those that nodulated only siratro were: *Ensifer* spp. 1E, 15A, 18C; *Paraburkholderia* spp. 21, 22.2, 40; *Rhizobium* sp. 21A and *Mesorhizobium* sp. 21C. In all these cases, we confirmed that the 16S rRNA sequences generated for the isolates obtained from the sirato and cowpea nodules matched those of the isolates originally used in the individual nodulation tests. Although we could not prove nodulation for all 88 isolates, it could be possible that we have not been able to mimic the conditions required for successful nodulation in the glasshouse. A point also made by Elliott et al. (2007a), when they could not prove nodulation of *Aspalathus* species by *P. tuberum*. The hosts cowpea and siratro are also routinely used during nodulation tests as they are considered promiscuous, this is, however, a generalisation and we have encountered situations where it appears as if one of these hosts are less readily nodulated by certain rhizobial isolates (data not shown).

When investigating the recovery of specific rhizobial genera from the soil samples collected in the seven biomes (i.e., Albany Thicket, Desert, Fynbos, Grassland, Nama-Karoo, Savanna and Succulent Karoo), *Ensifer* isolates were obtained from six of the biomes but were not encountered in soils from the Savanna biome (Table 1 and Figure 1C). In this biome only isolates of *Mesorhizobium* and *Rhizobium* were recovered, where *Mesorhizobium* were commonly isolated (i.e., 17 of the 21 isolates recovered from this biome) (Table 1 and Figure 1C). *Mesorhizobium* isolates were not found in soils from of the Nama-Karoo or Fynbos biomes. Four of the *Rhizobium* isolates were recovered from Savanna soil, while one isolate originated from the Succulent Karoo in the Western Cape and one from the Grassland biome (Figure 1C and Table 1). Of the 19 *Paraburkholderia* isolates, 14 were recovered from soils of the Fynbos biome in the Western Cape, while five were found in soils from the Succulent Karoo and one was recovered from the Albany Thicket biome (Figure 1C and Table 1). The two *Burkholderia* isolates (9A and 9C) and single *Agrobacterium* isolate (11A) were all recovered from Grassland biome soil, while the only *Bradyrhizobium* isolate originated from soil collected in the Nama-Karoo biome. Based on our data, the Grassland biome soils apparently harboured a diverse assemblage of bacteria capable of interacting with *V. karroo* (either as nodule symbionts or perhaps nodule endophytes) as isolates from five genera were recovered in these samples.

In order to delineate the isolates examined in this study to species level, we utilised the *recA* sequences. Following Nixon and Wheeler’s (1990) approach, we regarded the smallest diagnosable and bootstrap-supported groups of isolates on our phylogenetic trees as putative species. In this way, a total of 26 putative species (designated VK-1 to VK-26) were identified among 85 isolates (Figure 1B). The two *Burkholderia* isolates and single *Agrobacterium* isolate were not included in these analyses, as the focus of our study is potential rhizobia and there is currently very little evidence that these isolates can form functional nodules. As far as the authors are aware there is only one study which has proven that an *Agrobacterium* isolate (IRBG74) can successfully nodulate *Sesbania* spp. and does contain a symbiotic plasmid (Cummings et al., 2009). Note also, that the overall species groupings recovered from these phylogenetic trees (Supplementary Figures S1–S5) generally matched those reported in other studies (Martens et al., 2008; Rivas et al., 2009; Degefu et al., 2011, 2013a; Estrada-de los Santos et al., 2013).

Although *Ensifer* species made up the bulk of the sample set, we only delineated three putative species (VK-1 to VK-3) among the 32 isolates (Supplementary Figure S1). VK-1 contained four of our isolates (i.e., *Ensifer* spp. 5A-D), as well as isolate AC11d obtained from *Vachellia seyal* in Ethiopia (Degefu et al., 2012). VK-3 was represented by a large group, containing isolates from a variety of *Vachellia* hosts in Ethiopia (Degefu et al., 2012), as well as our isolate 1E. The remainder of our *Ensifer* isolates formed part of VK-2, which also included Ethiopian isolates (from various *Vachellia* species), as well as an isolate from the root nodules of *Lessertia* from South Africa (Lemaire et al., 2015a). None of our isolates showed a close phylogenetic relationship with those from *Vachellia jacquemontii* or *Tephrosia* spp. from India (Gehlot et al., 2012; Sankhla et al., 2017), nor to isolates of *Vachellia tortilis* from Morocco (Sakrouhi et al., 2016). No known species appeared to be closely related to VK-3, while the closest phylogenetic neighbour of VK-1 and VK-2 was *E. alkalisoli* (98% bootstrap support) and *E. fredii* (87% bootstrap support), respectively.

The *Mesorhizobium* isolates from this study separated into nine putative species (VK-4 to VK-12) (Supplementary Figure S2). Of these, five were represented by single isolates (VK-5, VK-8, VK-9, VK-10, and VK-12), none of which were closely related to known *Mesorhizobium* species. Two of our putative species are likely conspecific with *M. abyssinicae* (i.e., VK-4 with 93% bootstrap support) and *M. shonense* (i.e., VK-6 with 89% bootstrap support), respectively. Both VK-4 and VK-6 also contained several other *Vachellia*- or *Senegalia*-associated isolates from Ethiopia (Degefu et al., 2011). The remaining two putative species (VK-7 and VK-11) included isolates from our study only, and neither received bootstrap-support for their relationships with known species of *Mesorhizobium*. The phylogenetic analysis included many South African isolates of *Mesorhizobium* (predominantly from papilionoid hosts) (Supplementary Figure S2), but they did not group closely with any of our isolates from *V. karroo*.

**FIGURE 2:**
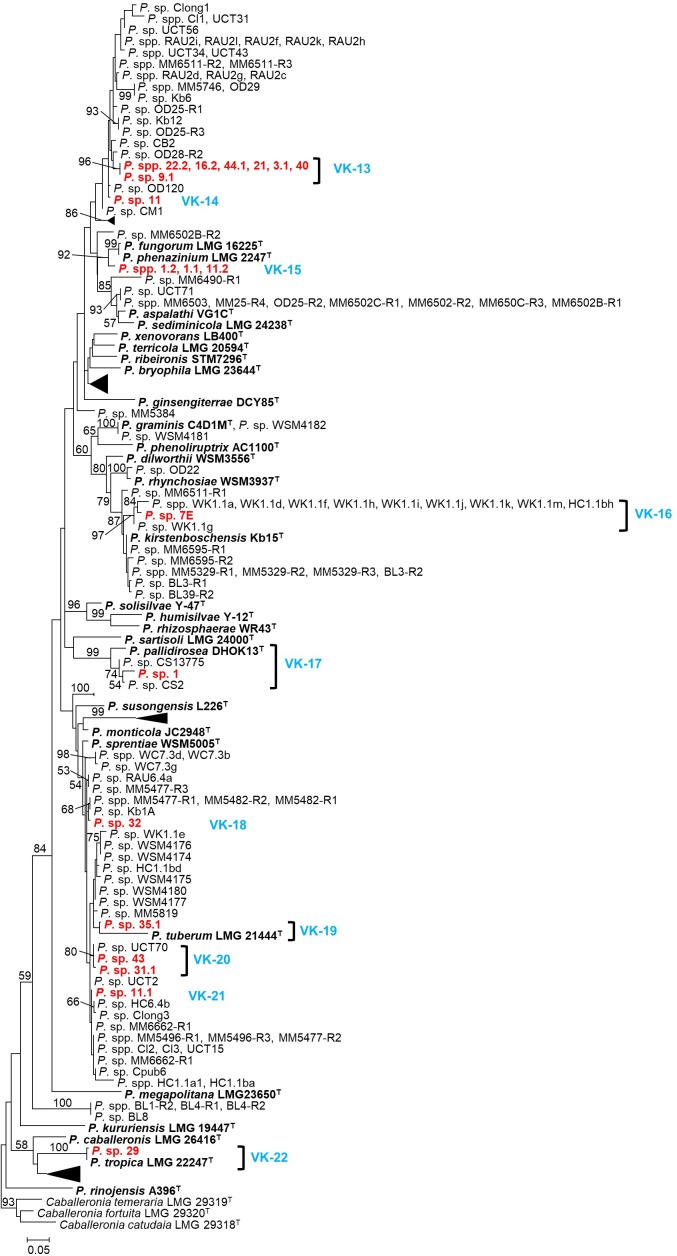
A maximum-likelihood phylogeny of the *recA* locus for the beta-rhizobial genus *Paraburkholderia*. Isolates from this study are listed in red, while type strains of species appear in bold. The delineated *Vachellia karroo*-associated lineages (VK-13 to VK-22) appear in blue. In order to minimize the phylogeny some lineages (which do not contain any isolates from this study), have been collapsed. The expanded tree, containing information for the host or source and geographic origin of each isolate, appears as Supplementary Figure S3. GenBank accession numbers for all the isolates included in this phylogeny are listed in Supplementary Table S3. Nodes with ≥50% are indicated, while the scale bar shows the number of nucleotide substitutions per site.

The 19 *Paraburkholderia* isolates represented ten putative species (VK-13 to VK-22) (Figure 2; Supplementary Figure S3). Of these, five (VK-14, VK-18, VK-19, VK-21, and VK-22) were represented by single isolates (Figure 2 and Supplementary Figure S3), while VK-15 consisted of three isolates representing one haplotype. The remaining four putative species all associated with South African papilionoid legumes only: VK-13 consisted of isolates from this study only (96% support), VK-16 included ten symbionts of *Hypocalyptus sophoroides* (Beukes et al., 2013), VK-17 included isolate CS13775 from *Indigofera ionii* (Lemaire et al., 2016a) and VK-20 included isolate UCT70 from *Cyclopia maculata* (Kock, 2003; Beukes et al., 2013). The putative species VK-13, VK-14, VK-18, VK-19, and VK-20 did not form supported groupings with known *Paraburkholderia* species. Of the remaining five, VK-15 was closely related to two species (i.e., *P. phenazinium* and *P. fungorum* with 92% bootstrap support), VK-16 was closely related to *P. kirstenboschensis* (87% bootstrap support), VK-17 grouped closely with *P. pallidirosa* (99% bootstrap support), VK-19 grouped with *P. tuberum* (80% bootstrap support), while VK-22 were very closely related and perhaps conspecific with *P. tropica* (100% bootstrap support) (Figure 2 and Supplementary Figure S3).

The six *Rhizobium* isolates separated into three putative species (VK-23 to VK-25) (Supplementary Figure S4). Of these, VK-23 was represented by a single isolate and was placed as a sister taxon to a group consisting of *R. sophorae* and *R. sophoriradicis* (51% bootstrap). VK-24, including four of our isolates, was closely related to *R. calliandrae* (57% bootstrap support). VK-25 was also represented by a single isolate (1B) and was closely related to *R. endophyticum* (93% bootstrap support). The single *Bradyrhizobium* isolate recovered in this study (15B) represented putative species VK-26, which appeared to be closely related to *B. liaoningense* (53% bootstrap support; Supplementary Figure S5).

## Discussion

This study investigated the root nodule bacteria of *V. karroo* sampled across a broad geographical area in South Africa, spanning sites in six provinces and seven biomes. Our results revealed that taxonomically diverse bacteria from both the Alpha- and Betaproteobacteria, occupy the root nodules of this legume. Among the 88 isolates collected, 66 belonged to various genera in the Alphaproteobacteria, while 19 were placed in the Betaproteobacteria. These bacteria separated into 26 putative species (VK-1 to VK-26) with representatives from the alpha-rhizobial genera *Ensifer, Mesorhizobium*, *Rhizobium*, and *Bradyrhizobium* and the beta-rhizobial genus *Paraburkholderia*. Three of the isolates examined were from genera (*Agrobacterium* and *Burkholderia*) with no known rhizobia (Mrabet et al., 2006; Vial et al., 2011; Mousavi et al., 2014) and most probably represent endophytes. These three isolates were also not successful in nodulation tests, which is congruent with the fact that members of *Burkholderia* sensu stricto do not nodulate legumes (Gyaneshwar et al., 2011). Therefore, apart from identifying for the first time the diverse rhizobial symbionts associating with South African *V. karroo*, this study also extends the known number of beta-rhizobial isolates able to associate with an indigenous South African member of the mimosoid clade.

Although some genera appeared to be common symbionts of *V. karroo*, they were not necessarily common in all the biomes examined. *Ensifer*, for instance, was found in all biomes sampled except for the Savanna biome, while *Mesorhizobium* was particularly common in this biome, but not encountered in the Fynbos or Nama-Karoo biomes. This trend is generally consistent with Mucina and Rutherford’s (2006) view of biomes as “subcontinental biotic super communities.” Accordingly, each community consists of a similar vegetation structure exposed to similar macroclimatic patterns. For example, the Succulent Karoo biome represents a semidesert and the Fynbos biome is a relatively moist winter-rainfall region. The Savanna and Grassland biomes are both summer rainfall regions, but because the Grassland biome is part of the elevated interior of South Africa, it is generally cooler than the Savanna biome (Mucina and Rutherford, 2006). The Albany Thicket biome is characterised by fragmented vegetation types due to the two climate systems present in the area (all-year rainfall in the southwest and summer rainfall in the northeast) (Mucina and Rutherford, 2006). The Desert biome experiences the lowest rainfall and highest temperatures of all South African biomes (Mucina and Rutherford, 2006). Different sets of environmental factors therefore impact upon the ecology of *V. karroo* in the various biomes. These factors likely also play key roles in shaping, not only the rhizosphere communities associated with this plant, but also its rhizobial symbionts.

As suggested previously in *Paraburkholderia*, the diversity and distribution of a particular rhizobial group may be determined by environmental factors, as well as life history traits. In the current study, the members of this genus were more prevalent in the Fynbos biome, as was also shown in previous studies (Lemaire et al., 2015b, 2016a,b). This is consistent with the notion that South Africa represents one of the two known centres of diversity for this genus, with the other centre being South America (Gyaneshwar et al., 2011; Beukes et al., 2013; Liu et al., 2014). In *Paraburkholderia* it has been shown that, instead of legume host range, various environmental factors shape species distribution (Garau et al., 2009; Bontemps et al., 2010; Beukes et al., 2013). These include soil pH (Garau et al., 2009; Bontemps et al., 2010; Gyaneshwar et al., 2011; Mishra et al., 2012; Beukes et al., 2013; de Castro Pires et al., 2018; Silva et al., 2018), and elevation (especially its influence on temperature and precipitation levels) (Bontemps et al., 2010; Beukes et al., 2013; Lemaire et al., 2015a). The tendency of papilionoid legumes in the Cape Floristic Region or Fynbos biome to be nodulated by *Paraburkholderia* (Elliott et al., 2007a; Beukes et al., 2013; de Meyer et al., 2013a,b; Howieson et al., 2013) may therefore be comparable to that of the *Mimosa* hosts growing in the Cerrado and Caatinga regions of Brazil (Bontemps et al., 2010; dos Reis et al., 2010), as both of these regions have acidic soils that are low in nutrients (Suárez-Moreno et al., 2012; Beukes et al., 2013; Ndlovu et al., 2013). However, *Mimosa* hosts in Brazil can associate with *Rhizobium* species when the soil pH is neutral to alkaline (Baraúna et al., 2016). *Dipogon lignosus*, an indigenous South African legume from the tribe Phaseoleae (Liu et al., 2014; Lemaire et al., 2016a) is also found as a weed in New Zealand (Liu et al., 2014) where it associates with *Paraburkholderia dipogonis* (Sheu et al., 2015; Dobritsa and Samadpour, 2016) in soils with an acidic pH but with *Bradyrhizobium* or *Rhizobium* in soils with a pH closer to neutral (Liu et al., 2014). These acidic soil conditions are apparently ideally suited to *Paraburkholderia* species which is why they can outcompete co-existing alpha-rhizobia (Elliott et al., 2009; Suárez-Moreno et al., 2012; Beukes et al., 2013). Similar factors likely also determine the ecological success of the various rhizobial groups in the other six biomes sampled here.

The first report of a rhizobial association between *V. karroo* and a beta-rhizobium, was a study by Moulin et al. (2014) which showed that *Paraburkholderia phymatum* STM815^T^ can effectively nodulate this host. The current study contributes another 19 *Paraburkholderia* isolates with a South African origin to the growing group of beta-rhizobia that can potentially associate with this mimosoid host. In other words, in addition to species of papilionoid genera (e.g., *Virgilia*, *Aspalathus*, *Amphithalea*, *Hypocalyptus*, *Cyclopia*, and *Indigofera*) (Beukes, 2012; Beukes et al., 2013; Lemaire et al., 2015a, 2016a), *Paraburkholderia* also nodulates *V. karroo*. This finding is important as symbiotic gene phylogenies have shown that nodulating *Paraburkholderia* species can be sub-divided into so-called mimosoid and papilionoid nodulating groups (Bontemps et al., 2010; Beukes et al., 2013; Estrada-de los Santos et al., 2018). Only two species, *P. tuberum* and *P. phymatum*, are apparently capable of nodulating both papilionoid and mimosoid hosts (Elliott et al., 2007b; Bontemps et al., 2010; Mishra et al., 2012; Suárez-Moreno et al., 2012). In the case of *P. tuberum*, the biovars mimosae and papilionoideae have been proposed for differentiating among isolates (Mishra et al., 2012). In *P. tuberum*, this split likely correlates with the distinct origin of the symbiotic loci of the mimosoid and papilionoid-nodulating species (Beukes et al., 2013; de Meyer et al., 2016; Lemaire et al., 2016a). Our future work will seek to obtain sequences for the symbiotic loci of the *V. karroo Paraburkholderia* isolates and to compare these with sequences from South African papilionoid and South American mimosoid isolates.

Most of the isolates obtained from *V. karroo* root nodules were members of the alpha-rhizobial genera *Ensifer* (32 isolates) and *Mesorhizobium* (27 isolates). Both of these genera include a number of species that have been described from *Acacia* sensu lato species, and includes *Ensifer kostiensis* (from *Senegalia senegal* in Sudan) (Nick et al., 1999), *Ensifer terangae* (from *S. laeta* in Senegal) (de Lajudie et al., 1994), *Ensifer mexicanus (*from *Acaciella angustissima* in Mexico) (Lloret et al., 2007), *Ensifer chiapanecum* (from *Ac. angustissima* in Mexico) (Rincón-Rosales et al., 2009), *Ensifer americanus* (from *Acacia acatlensis* in Mexico, now *Mariosousa acatlensis*) (Toledo et al., 2003; Seigler et al., 2006), *M. abyssinicae* (from *V. abyssinica* in Ethiopia) (Degefu et al., 2013b), *M*. *shonense* (from *V. abyssinicae* in Ethiopia) (Degefu et al., 2013b) and *M. plurifarium* (from *S. senegal* in Senegal) (de Lajudie et al., 1998). Some of the *Mesorhizobium* isolates obtained from *V. karroo* are likely conspecific with these previously identified bacteria, particularly *M. abyssinicae* and *M. shonense*. The three putative *Ensifer* species obtained from *V. karroo* in the current study are likely conspecific with a variety of rhizobia from *V. seyal*, *V. tortilis* and *V. abyssinica* originating from Ethiopia and that have not yet been formally described (Degefu et al., 2012).

Unlike the genera *Ensifer* and *Mesorhizobium*, the genus *Rhizobium* does not currently contain any named species originating from nodules of legumes in *Acacia* sensu lato. There are, however, examples where studies have encountered isolates with high similarity to *R. leucaenae* (McInroy et al., 1999; Romdhane et al., 2006; Ribeiro et al., 2012), *R. giardinii* (now *Pararhizobium giardinii*; Mousavi et al., 2015), *R. etli* and *R. leguminosarum* (Wolde-meskel et al., 2005). Our study followed a similar trend with none of the identified putative species obtained from *V. karroo* being conspecific to known species. Nevertheless, none of the *Rhizobium* isolates from *V. karroo* showed significant similarity to isolates previously identified from *Acacia* sensu lato.

The alpha-rhizobial data from this study is comparable to what has been found previously for other African Acacias (i.e., *Vachellia* and *Senegalia* species), which are predominantly nodulated by isolates of *Mesorhizobium*, *Rhizobium* and *Ensifer* (Leary et al., 2006). The *Senegalia* hosts previously examined and for which rhizobial sequence data is available, are *S. senegal* (Ethiopia, Kenya, Mauritania, Senegal, Sudan) that associates with *Mesorhizobium*, *Rhizobium* and *Ensifer* (de Lajudie et al., 1998; McInroy et al., 1999; Nick et al., 1999; Sarr et al., 2005; Wolde-meskel et al., 2005; Fall et al., 2008; Degefu et al., 2011) and *S. laeta* (Senegal) that associates with *Ensifer* (de Lajudie et al., 1994). The *Vachellia* species that were previously investigated included *V. abyssinica* (Ethiopia), *V. nilotica* (Algeria, Mauritania, Senegal, Zimbabwe), *V. seyal* (Algeria, Ethiopia, Senegal), *V. tortilis* (Algeria, Ethiopia, Kenya, Mauritania, Morocco, Senegal, Tunisia) that all have *Mesorhizobium*, *Rhizobium*, and *Ensifer* symbionts (de Lajudie et al., 1994, 1998; McInroy et al., 1999; Ba et al., 2002; Sarr et al., 2005; Wolde-meskel et al., 2005; Romdhane et al., 2006; Diouf et al., 2007; Degefu et al., 2011, 2013b; Abdi et al., 2012; Boukhatem et al., 2012), as well as *V. horrida* (Morocco) that associates with *Ensifer* (Khbaya et al., 1998). Although *V. karroo* in Algeria and Kenya have been reported to associate with *Rhizobium* and *Ensifer* (McInroy et al., 1999; Boukhatem et al., 2012), our study shows for the first time that this legume is nodulated by *Bradyrhizobium*, *Mesorhizobium* and *Paraburkholderia* in addition to *Rhizobium* and *Ensifer*, in its native range in South Africa.

*Vachellia karroo* and Australian *Acacia* (*Acacia* sensu stricto) appear to differ markedly in their symbiotic preference. Only one *Bradyrhizobium* isolate was recovered here, which is very low when compared to *Acacia* s. s. species which predominantly nodulates with this alpha-rhizobial genus (Marsudi et al., 1999; Lafay and Burdon, 2001; Rodríguez-Echeverría et al., 2011; Crisóstomo et al., 2013). Leary et al. (2006) attributed these differences in symbiont associations in Africa and Australia to an apparent correlation between host phylogeny and the rhizobial groups nodulating them. They reported that the majority of Australian acacias nodulated with *Bradyrhizobium* while African and American acacias nodulated with *Ensifer* and *Mesorhizobium* species. However, improved resolution of legume taxonomy and wider sampling of symbionts suggest that the distribution of these associations is more complex. For example, certain species in the tribe Ingeae, a close relative of *Acacia* s. s. (including the Australian *Acacia*) (LPWG, 2013) are also nodulated by *Bradyrhizobium* species (Robinson and Harris, 2000; Miller and Bayer, 2001, 2003; Leary et al., 2006), but several *Calliandra* species (tribe Ingeae) associate with *Paraburkholderia* (Silva et al., 2018). Additionally, African acacias (represented by *Vachellia* and *Senegalia)* are mostly nodulated by *Mesorhizobium* and *Ensifer* species (Zhang et al., 1991; Ba et al., 2002; Leary et al., 2006), but their close relatives in the Piptadenia group (including the genus Mimosa; LPWG, 2013) associate predominantly with *Paraburkholderia* and other beta-rhizobia (Suárez-Moreno et al., 2012; Taulé et al., 2012; Bournaud et al., 2013).

It is more likely that the observed legume-rhizobium distribution patterns reflect the symbiotic compatibilities between the partners. This is because the symbiosis rely on successful recognition of the rhizobial Nod factor by the host nodulin genes (Ferro et al., 2000). Ferro et al. (2000) demonstrated that the Nod factors of rhizobia in the genera *Rhizobium*, *Mesorhizobium* and *Ensifer*, which associate with *Vachellia* or *Senegalia* hosts, does not contain a glycosyl group and are instead predominantly sulfated (Ferro et al., 2000). By contrast, *Bradyrhizobium* isolates associating with *Faidherbia albida* (previously *Acacia albida*; phylogenetically closely related to *Acacia* sensu stricto) produce Nod factors that are sulfated at a different postion and that are also fucosylated (Ferro et al., 2000).

The single *Bradyrhizobium* *V. karroo*-associated isolate (15B) obtained in this study grouped with *B. liaoningense* (originally isolated from *Glycine max* nodules in China) (Xu et al., 1995). Although an isolate obtained from *Vachellia* *sieberiana* var. *woodii* (formerly *Acacia* *sieberiana* var. *woodii*; Kyalangalilwa et al., 2013) in a previous study was designated *B. liaoningense*, that identification should be viewed with caution as it was based on partial 16S rRNA gene sequence information (Jaftha, 2000) which is known to have limited resolution in *Bradyrhizobium* at the species level (Willems et al., 2001; Vinuesa et al., 2008). Also, *B. liaoningense* is believed to be scarce in southern African soils (Steenkamp et al., 2008). Increasing the sample size in follow-up studies would facilitate a better geographical representation and analysis of whether the low numbers and diversity of *Bradyrhizobium* strains associated with *V. karroo* in South Africa are indeed a true reflection of what is occurring in these environments.

Taken together, our findings confirmed that the symbionts of *V. karroo* were indeed members of the alpha-rhizobial genera *Mesorhizobium, Ensifer* and *Rhizobium*. We were also able to isolate 19 South African *Paraburkholderia* isolates from the root nodules of this mimosoid host. With regard to the distribution of the *V. karroo*-symbionts across the various biomes, our study is also the first to report the legume-*Paraburkholderia* association in regions outside the Fynbos biome (i.e., one and three putative species were recovered as symbionts of *V. karroo* in one and four locations in the Albany Thicket and Succulent Karoo biomes, respectively). We also showed that a high diversity of rhizobial symbionts associate with *V. karroo* in the various regions, even in the Grassland biome where we identified putative species from three genera. The latter is important because *V. karroo* contributes significantly to bush encroachment (i.e., the change of grasslands to ecosystems containing more shrubs and trees), which is particularly evident in the Grassland biome in the Eastern Cape, North West and Free State provinces of South Africa (Dingaan and du Preez, 2018). The promiscuity of *V. karroo* in these and other settings therefore suggest that finding a suitable rhizobial partner is not a hurdle to the establishment or spread of this legume and likely represents an important driver or facilitator of its ecological success.

## Author Contributions

CB and FB planned the study, obtained the samples and cultures, tested for nodulation ability, analysed datasets, submitted sequence data, interpreted the results, and wrote the manuscript. FP and AH assisted with testing the nodulation ability, proofread the manuscript. MlR and TS proofread the manuscript. SV and ES planned the study, interpreted the results, wrote and proofread the manuscript.

## Conflict of Interest Statement

The authors declare that the research was conducted in the absence of any commercial or financial relationships that could be construed as a potential conflict of interest.
