# Supplementary material for: Both Alpha- and Beta-Rhizobia Occupy the Root Nodules of Vachellia karroo in South Africa
Source: Front Microbiol. 2019 Jun 4;10:1195. doi: 10.3389/fmicb.2019.01195 (PMC6558075; doi:10.3389/fmicb.2019.01195)
Supplement: Supplementary file 1 [file Table_1.DOCX]

**Supplementary Table S1** Accession numbers, legume host or source, country of origin and references for the *Ensifer* strains included in this study

| **Isolate** | ***RecA*** | **Host/Niche** | **Country** | **Reference** |
| --- | --- | --- | --- | --- |
| *E. adhaerens* LMG20216^T^ | AJ505595 | Soil | USA | Casida, 1982 |
| *E. alkalisoli* YIC4027^T^ | A8M32_11435 | *Sesbania cannabina* | China | Li et al., 2017 |
| *E. americanus* CFNEI156^T^ | ATC00_RS01925 | *Acacia* sp. | Mexico | Toledo et al., 2003 |
| *E. arboris* HAMBI1552^T^ | AM182130 | *Prosopis chilensis* | Sudan | Nick et al., 1999 |
| *E. chiapanecum* ITTG S70^T^ | EF463926 | *Acaciella angustissima* | Mexico | Rincón-Rosales et al., 2009 |
| *E. fredii* LMG6217^T^ | AM182145 | *Glyxine max* | China | Willems and Collins, 1993 |
| *E. garamanticus* ORS1400^T^ | AM946573 | *Argyrolobium uniflorum* | Tunisia | Merabet et al., 2010 |
| *E. glycinis* CCBAU23380^T^ | AU381_RS03615 | *Astragalus mongholicus* | China | Yan et al., 2016 |
| *E. kostiensis* LMG19227^T^ | AM182142 | *Senegalia senegal* | Sudan | Nick et al., 1999 |
| *E. kummerowiae* CCBAU71714^T^ | DQ411945 | *Kummerowia stipulacea* | China | Wei et al., 2002 |
| *E. medicae* 11-3 21a^T^ | AM182135 | *Medicago truncatula* | France | Rome et al., 1996 |
| *E. meliloti* IAM12611^T^ | AM182133 | *Medicago sativa* | USA | de Lajudie et al., 1994 |
| *E. mexicanus* ITTG-R7^T^ | DQ411951 | *Acacia angustissima* | Mexico | Lloret et al., 2007 |
| *E. morelensis* Lc04^T^ | AJ505601 | *Leucaena leucocephala* | Mexico | Wang et al., 2002 |
| *E. numidicus* ORS1407^T^ | AM946576 | *Argyrolobium uniflorum* | Tunisia | Merabet et al., 2010 |
| *E. psoraleae* CCBAU65732^T^ | EU622106 | *Psoralea corylifolia* | China | Wang et al., 2013 |
| *E. saheli* LMG7837^T^ | ATB98_RS05685 | *Sesbania cannabina* | Senegal | de Lajudie et al., 1994 |
| *E. sesbaniae* CCBAU65729^T^ | JX028823 | *Sesbania cannabina* | China | Wang et al., 2013 |
| *E. shofinae* CCBAU251167^T^ | BB335_RS02205 | *Glycine max* | China | Chen et al., 2017 |
| *E. sojae* CCBAU05684^T^ | IC28_RS13770 | *Glycine max* | China | Li et al., 2011 |
| *E. terangae* LMG7834^T^ | AM182153 | *Senegalia laeta* | Senegal | de Lajudie et al., 1994 |
| *E.* sp. 1C | LN890754 | *Vachellia karroo* | South Africa | This study |
| *E.* sp. 1E | LN890784 | *Vachellia karroo* | South Africa | This study |
| *E.* sp. 5A | LN890782 | *Vachellia karroo* | South Africa | This study |
| *E.* sp. 5B | LN890780 | *Vachellia karroo* | South Africa | This study |
| *E.* sp. 5D | LN890783 | *Vachellia karroo* | South Africa | This study |
| *E.* sp. 5C | LN890781 | *Vachellia karroo* | South Africa | This study |
| *E.* sp. 6A | LN890779 | *Vachellia karroo* | South Africa | This study |
| *E.* sp. 6D | LN890753 | *Vachellia karroo* | South Africa | This study |
| *E.* sp. 7A | LN890755 | *Vachellia karroo* | South Africa | This study |
| *E.* sp. 7B | LN890756 | *Vachellia karroo* | South Africa | This study |
| *E.* sp. 8A | LN890757 | *Vachellia karroo* | South Africa | This study |
| *E.* sp. 8B | LN890758 | *Vachellia karroo* | South Africa | This study |
| *E.* sp. 10A | LN890759 | *Vachellia karroo* | South Africa | This study |
| *E.* sp. 10C | LN890760 | *Vachellia karroo* | South Africa | This study |
| *E.* sp. 10E | LN890761 | *Vachellia karroo* | South Africa | This study |
| *E.* sp. 11D | LN890762 | *Vachellia karroo* | South Africa | This study |
| *E.* sp. 12A | LN890763 | *Vachellia karroo* | South Africa | This study |
| *E.* sp. 13E | LN890764 | *Vachellia karroo* | South Africa | This study |
| *E.* sp. 14C | LN890765 | *Vachellia karroo* | South Africa | This study |
| *E.* sp. 15A | LN890766 | *Vachellia karroo* | South Africa | This study |
| *E.* sp. 15C | LN890767 | *Vachellia karroo* | South Africa | This study |
| *E.* sp. 15D | LN890768 | *Vachellia karroo* | South Africa | This study |
| *E.* sp. 15E | LN890769 | *Vachellia karroo* | South Africa | This study |
| *E.* sp. 16A | LN890770 | *Vachellia karroo* | South Africa | This study |
| *E.* sp. 16B | LN890771 | *Vachellia karroo* | South Africa | This study |
| *E.* sp. 16D | LN890772 | *Vachellia karroo* | South Africa | This study |
| *E.* sp. 16E | LN890773 | *Vachellia karroo* | South Africa | This study |
| *E.* sp. 18A | LN890774 | *Vachellia karroo* | South Africa | This study |
| *E.* sp. 18C | LN890775 | *Vachellia karroo* | South Africa | This study |
| *E.* sp. 18D | LN890776 | *Vachellia karroo* | South Africa | This study |
| *E.* sp. 18E | LN890777 | *Vachellia karroo* | South Africa | This study |
| *E.* sp. 19A | LN890778 | *Vachellia karroo* | South Africa | This study |
| *E.* sp. AC16c | JF450577 | *Vachellia seyal* | Ethiopia | Degefu et al., 2012 |
| *E.* sp. AC17c | JF450580 | *Vachellia seyal* | Ethiopia | Degefu et al., 2012 |
| *E.* sp. AC20b | JF450587 | *Vachellia tortilis* | Ethiopia | Degefu et al., 2012 |
| *E.* sp. AC24d1 | JF450592 | *Vachellia tortilis* | Ethiopia | Degefu et al., 2012 |
| *E.* sp. AC24d2 | JF450593 | *Vachellia tortilis* | Ethiopia | Degefu et al., 2012 |
| *E.* sp. AC10d | JF450570 | *Vachellia seyal* | Ethiopia | Degefu et al., 2012 |
| *E.* sp. AC38d1 | JF450610 | *Vachellia*  *abyssinica* | Ethiopia | Degefu et al., 2012 |
| *E.* sp. AC38d2 | JF450611 | *Vachellia abyssinica* | Ethiopia | Degefu et al., 2012 |
| *E.* sp. AC11d | JF450571 | *Vachellia seyal* | Ethiopia | Degefu et al., 2012 |
| *E.* sp. AC28d1 | JF450607 | *Vachellia tortilis* | Ethiopia | Degefu et al., 2012 |
| *E.* sp. AC28a | JF450606 | *Vachellia tortilis* | Ethiopia | Degefu et al., 2012 |
| *E.* sp. AC25e | JF450598 | *Vachellia tortilis* | Ethiopia | Degefu et al., 2012 |
| *E.* sp. AC25d | JF450597 | *Vachellia tortilis* | Ethiopia | Degefu et al., 2012 |
| *E.* sp. AC19e | JF450586 | *Vachellia tortilis* | Ethiopia | Degefu et al., 2012 |
| *E.* sp. AC17b | JF450579 | *Vachellia seyal* | Ethiopia | Degefu et al., 2012 |
| *E.* sp. AC07c | JF450563 | *Vachellia seyal* | Ethiopia | Degefu et al., 2012 |
| *E.* sp. AC07b2 | JF450562 | *Vachellia seyal* | Ethiopia | Degefu et al., 2012 |
| *E.* sp. AC07b1 | JF450561 | *Vachellia seyal* | Ethiopia | Degefu et al., 2012 |
| *E.* sp. AC17d | JF450581 | *Vachellia seyal* | Ethiopia | Degefu et al., 2012 |
| *E.* sp. AC07a | JF450560 | *Vachellia seyal* | Ethiopia | Degefu et al., 2012 |
| *E.* sp. AC14c | JF450572 | *Vachellia seyal* | Ethiopia | Degefu et al., 2012 |
| *E.* sp. AC18a | JF450584 | *Vachellia tortilis* | Ethiopia | Degefu et al., 2012 |
| *E.* sp. AC27d2 | JF450604 | *Vachellia tortilis* | Ethiopia | Degefu et al., 2012 |
| *E.* sp. AC16b1 | JF450575 | *Vachellia seyal* | Ethiopia | Degefu et al., 2012 |
| *E.* sp. AC21c1 | JF450588 | *Vachellia tortilis* | Ethiopia | Degefu et al., 2012 |
| *E.* sp. AC22d | JF450590 | *Vachellia tortilis* | Ethiopia | Degefu et al., 2012 |
| *E.* sp. AC25c | JF450596 | *Vachellia tortilis* | Ethiopia | Degefu et al., 2012 |
| *E.* sp. AC27e | JF450605 | *Vachellia tortilis* | Ethiopia | Degefu et al., 2012 |
| *E.* sp. AC40a | JF450612 | *Vachellia abyssinica* | Ethiopia | Degefu et al., 2012 |
| *E.* sp. AC27c | JF450602 | *Vachellia tortilis* | Ethiopia | Degefu et al., 2012 |
| *E.* sp. AC08a1 | JF450565 | *Vachellia seyal* | Ethiopia | Degefu et al., 2012 |
| *E.* sp. AC17a | JF450578 | *Vachellia seyal* | Ethiopia | Degefu et al., 2012 |
| *E.* sp. AC17e1 | JF450582 | *Vachellia seyal* | Ethiopia | Degefu et al., 2012 |
| *E.* sp. AC19a | JF450585 | *Vachellia tortilis* | Ethiopia | Degefu et al., 2012 |
| *E.* sp. AC08a2 | JF450566 | *Vachellia seyal* | Ethiopia | Degefu et al., 2012 |
| *E.* sp. AC08e | JF450567 | *Vachellia seyal* | Ethiopia | Degefu et al., 2012 |
| *E.* sp. AC25a | JF450594 | *Vachellia tortilis* | Ethiopia | Degefu et al., 2012 |
| *E.* sp. AC25b | JF450595 | *Vachellia tortilis* | Ethiopia | Degefu et al., 2012 |
| *E.* sp. AC17e2 | JF450583 | *Vachellia seyal* | Ethiopia | Degefu et al., 2012 |
| *E.* sp. AC07e | JF450564 | *Vachellia seyal* | Ethiopia | Degefu et al., 2012 |
| *E.* sp. AC16a | JF450574 | *Vachellia seyal* | Ethiopia | Degefu et al., 2012 |
| *E.* sp. AC27a1 | JF450599 | *Vachellia tortilis* | Ethiopia | Degefu et al., 2012 |
| *E.* sp. AC38b1 | JF450608 | *Vachellia abyssinica* | Ethiopia | Degefu et al., 2012 |
| *E.* sp. AC38b2 | JF450609 | *Vachellia abyssinica* | Ethiopia | Degefu et al., 2012 |
| *E.* sp. AC01e | JF450559 | *Vachellia seyal* | Ethiopia | Degefu et al., 2012 |
| *E.* sp. AC01c2 | JF450556 | *Vachellia seyal* | Ethiopia | Degefu et al., 2012 |
| *E.* sp. AC01b | JF450554 | *Vachellia seyal* | Ethiopia | Degefu et al., 2012 |
| *E.* sp. AC01c1 | JF450555 | *Vachellia seyal* | Ethiopia | Degefu et al., 2012 |
| *E.* sp. AC01d2 | JF450558 | *Vachellia seyal* | Ethiopia | Degefu et al., 2012 |
| *E.* sp. AC01d1 | JF450557 | *Vachellia seyal* | Ethiopia | Degefu et al., 2012 |
| *E.* sp. AC10a2 | JF450569 | *Vachellia seyal* | Ethiopia | Degefu et al., 2012 |
| *E.* sp. AC10a1 | JF450568 | *Vachellia seyal* | Ethiopia | Degefu et al., 2012 |
| *E.* sp. AC14d | JF450573 | *Vachellia seyal* | Ethiopia | Degefu et al., 2012 |
| *E.* sp. OD45 | KF802771 | *Indigofera* sp. | South Africa | Lemaire unpubl. |
| *E.* sp. OD46 | KF802773 | *Lessertia* sp. | South Africa | Lemaire unpubl. |
| *E.* sp. AN5 | JN089676 | *Vachellia tortilis* | Tunisia | Fterich et al., 2012 |
| *E.* sp. AW7 | JN089677 | *Vachellia tortilis* | Tunisia | Fterich et al., 2012 |
| *E.* sp. AW24 | JN089678 | *Vachellia tortilis* | Tunisia | Fterich et al., 2012 |
| *E.* sp. AG1 | JN089679 | *Vachellia tortilis* | Tunisia | Fterich et al., 2012 |
| *E.* sp. AG14 | JN089680 | *Vachellia tortilis* | Tunisia | Fterich et al., 2012 |
| *E.* sp. AG22 | JN089681 | *Vachellia tortilis* | Tunisia | Fterich et al., 2012 |
| *E.* sp. AB3 | JN089683 | *Vachellia tortilis* | Tunisia | Fterich et al., 2012 |
| *E.* sp. AB32 | JN089684 | *Vachellia tortilis* | Tunisia | Fterich et al., 2012 |
| *E.* sp. LMR001 | KP993231 | *Vachellia gummifera* | Morocco | Sakrouhi et al., 2016 |
| *E.* sp. LMR002 | KP993232 | *Vachellia tortilis* | Morocco | Sakrouhi et al., 2016 |
| *E.* sp. LMR003 | KT991374 | *Vachellia tortilis* | Morocco | Sakrouhi et al., 2016 |
| *E.* sp. LMR004 | KT991375 | *Vachellia tortilis* | Morocco | Sakrouhi et al., 2016 |
| *E.* sp. LMR005 | KT991376 | *Vachellia tortilis* | Morocco | Sakrouhi et al., 2016 |
| *E.* sp. LMR006 | KP993233 | *Vachellia tortilis* | Morocco | Sakrouhi et al., 2016 |
| *E.* sp. LMR007 | KT991377 | *Vachellia tortilis* | Morocco | Sakrouhi et al., 2016 |
| *E.* sp. LMR009 | KT991378 | *Vachellia tortilis* | Morocco | Sakrouhi et al., 2016 |
| *E.* sp. LMR010 | KT991379 | *Vachellia tortilis* | Morocco | Sakrouhi et al., 2016 |
| *E.* sp. LMR012 | KT991380 | *Vachellia tortilis* | Morocco | Sakrouhi et al., 2016 |
| *E.* sp. LMR013 | KP993235 | *Vachellia tortilis* | Morocco | Sakrouhi et al., 2016 |
| *E.* sp. LMR015 | KT991382 | *Vachellia tortilis* | Morocco | Sakrouhi et al., 2016 |
| *E.* sp. LMR016 | KT991383 | *Vachellia tortilis* | Morocco | Sakrouhi et al., 2016 |
| *E.* sp. LMR017 | KT991384 | *Vachellia tortilis* | Morocco | Sakrouhi et al., 2016 |
| *E.* sp. LMR018 | KT991385 | *Vachellia tortilis* | Morocco | Sakrouhi et al., 2016 |
| *E.* sp. LMR019 | KT991386 | *Vachellia tortilis* | Morocco | Sakrouhi et al., 2016 |
| *E.* sp. LMR020 | KT991387 | *Vachellia tortilis* | Morocco | Sakrouhi et al., 2016 |
| *E.* sp. LMR021 | KP993236 | *Vachellia tortilis* | Morocco | Sakrouhi et al., 2016 |
| *E.* sp. JNVU AJ32 | KU665602 | *Vachellia jacquemontii* | India | Sankhla et al., 2016 |
| *E.* sp. JNVU AJ14 | KU665607 | *Vachellia jacquemontii* | India | Sankhla et al., 2016 |
| *E.* sp. JNVU AJ10 | KU665608 | *Vachellia jacquemontii* | India | Sankhla et al., 2016 |
| *E.* sp. JNVU AJ18 | KU665606 | *Vachellia jacquemontii* | India | Sankhla et al., 2016 |
| *E.* sp. JNVU AJ23 | KU665605 | *Vachellia jacquemontii* | India | Sankhla et al., 2016 |
| *E.* sp. JNVU AJ24 | KU665604 | *Vachellia jacquemontii* | India | Sankhla et al., 2016 |
| *E.* sp. JNVU AJ31 | KU665603 | *Vachellia jacquemontii* | India | Sankhla et al., 2016 |
| *E.* sp. JNVU TF7 | KT803814 | *Tephrosia falciformis* | India | Gehlot et al., 2012 |
| *E.* sp. JNVU TW8 | KT803835 | *Tephrosia wallichii* | India | Gehlot et al., 2012 |
| *E.* sp. JNVU TW10 | KT803838 | *Tephrosia wallichii* | India | Gehlot et al., 2012 |
| *E.* sp. JNVU TP13 | KT803820 | *Tephrosia purpurea* | India | Gehlot et al., 2012 |
| *E.* sp. JNVU TV3 | KT803831 | *Tephrosia villosa* | India | Gehlot et al., 2012 |
| *E.* sp. JNVU TV1 | KT803824 | *Tephrosia villosa* | India | Gehlot et al., 2012 |
| *E.* sp. PC2 | B077_RS38300 | *Prosopis cineraria* | India | Gehlot et al., 2012 |
| *E.* sp. JNVU TP6 | KP993237 | *Tephrosia purpurea* | India | Tak et al., 2016 |
| *E.* sp. JNVU TL4 | KT803815 | *Tephrosia leptostachya* | India | Tak et al., 2016 |
| *E.* sp. JNVU RA9 | KP993239 | *Rhynchosia aurea* | India | Gehlot et al., 2012 |
| *E.* sp. Gs668 | KU041609 | *Genista saharae* | Algeria | Chaïch et al., 2016 |
| *E.* sp. CCNWSX1108 | JX524435 | *Glycine soja* | China | Zhao et al., 2014 |
| *E.* sp. X96C2 | KR232267 | *Vachellia xanthophloea* | Mozambique | Teixeira and Rodríguez-Echeverría, 2016 |
| *E.* sp. Gs6723 | KU042608 | *Genista saharae* | Algeria | Chaïch et al., 2016 |
| *E.* sp. X873B | KR232261 | *Vachellia xanthophloea* | Mozambique | Teixeira and Rodríguez-Echeverría, 2016 |

**References:**

Casida, L.E., Jr. (1982) *Ensifer adhaerens* gen. nov., sp. nov.: a bacterial predator of bacteria in soil. Int. J. Syst. Bacteriol. 32: 339-345

Chaïch, K., Bekki, A., Bouras, N., Holtz, M.D., Soussou, S., Mauré, L., Brunel, B., de Lajudie, P., Cleyet-Marel, J-C (2016) Rhizobial diversity associated with the spontaneous legume *Genista saharae* in the northeastern Algerian Sahara. Symbiosis doi: 10.1007/s13199-016-0414-y

Chen, W.H., Yang, S.H., Li, Z.H., Zhang, X.X., Sui, X.H., Wang, E.T., Chen, W.X., Chen, W.F. (2017) *Ensifer shofinae* sp. nov., a novel rhizobial species isolated from root nodules of soybean (*Glycine max*). Syst. Appl. Microbiol. Doi: http://dx.doi.org/10.1016/j.syapm.2017.01.002

Degefu, T., Wolde-Meskel, E., Frostegard, A. (2012) Phylogenetic multilocus sequence analysis identifies seven novel *Ensifer* genospecies isolated from a less-well-explored biogeographical region in East Africa. Int. J. Syst. Evol. Microbiol. 62: 2286-2295

De Lajudie, P., Willems, A., Pot, B., Dewettinck, D., Maestrojuan, G., Neyra, M., Collins, M.D., Dreyfus, B., Kersters, K., Gillis, M. (1994) Polyphasic taxonomy of rhizobia: emendation of the genus *Sinorhizobium* and description of *Sinorhizobium* *meliloti* comb. nov., *Sinorhizobium* *saheli* sp. nov., and *Sinorhizobium* *teranga* sp. nov. Int. J. Syst. Bacteriol. 44: 715-733

Fterich, A., Mahdhi, M., Lafuente, A., Pajuelo, E., Caviedes, M.A., Rodriguez-Llorente, I.D., Mars, M. (2012) Taxonomic and symbiotic diversity of bacteria isolated from nodules of *Acacia tortilis* subsp. *raddiana* in arid soils of Tunisia. Can. J. Microbiol. 58: 738-751

Gehlot, H.S., Panwar, D., Tak, N., Tak, A., Sankhla, I.S., Poonar, N., Parihar, R., Shekhawat, N.S., Kumar, M., Tiwari, R., Ardley, J., James, E.K., Sprent, J.I. (2012) Nodulation of legumes from the Thar desert of India and molecular characterization of their rhizobia. Plant Soil 357: 227-243

Li, Y., Yan, J., Yu, B., Wang, E.T., Li, X., Yan, H., Liu, W., Xie, Z. (2017) *Ensifer alkalisoli* sp. nov. isolated from root nodules of *Sesbania cannabina* grown in saline-alkaline soil. Int. J. Syst. Evol. Microbiol. 66: 5294-5300. doi: 10.1099/ijsem.0.001510

Li, Q.Q., Wang, E.T., Chang, Y.L., Zhang, Y.Z., Zhang, Y.M., Sui, X.H., Chen, W.F., Chen, W.X. (2011) *Ensifer sojae* sp. nov., isolated from root nodules of *Glycine* *max* grown in saline-alkaline soil. Int. J. Syst. Evol. Microbiol. 61: 1981-1988

Lloret, L., Ormeño-Orrillo, E., Rincón, R., Martínez-Romero, J., Rogel-Hernández, M.A., Martínez-Romero, E. (2007) *Ensifer mexicanus* sp. nov. a new species nodulating *Acacia angustissima* (Mill.) Kuntze in Mexico. Syst. Appl. Microbiol. 30: 280-290

Merabet, C., Martens, M., Mahdhi, M., Zakhia, F., Sy, A., Le Roux, C., Domergue, O., Coopman, R., Bekki, A., Mars, M., Willems, A., de Lajudie, P. (2010) Multilocus sequence analysis of root nodule isolates from *Lotus arabicus* (Senegal), *Lotus* *creticus*, *Argyrolobium uniflorum* and *Medicago sativa* (Tunisia) and description of *Ensifer numidicus* sp. nov. and *Ensifer garamanticus* sp. nov. Int. J. Syst. Evol. Microbiol. 60: 664-674

Nick, G., de Lajudie, P., Eardly, B.D., Suomalainen, S., Paulin, L., Zhang, X., Gillis, M., Lindström, K. (1999) *Sinorhizobium arboris* sp. nov. and *Sinorhizobium kostiense* sp. nov., isolated from leguminous trees in Sudan and Kenya. Int. J. Syst. Bacteriol. 49: 1359-1368

Rincón-Rosales, R., Lloret, L., Ponce, E., Martínez-Romero, E. (2009) Rhizobia with different symbiotic efficiensies nodulate *Acaciella angustissim*a in Mexico, including *Sinorhizobium chiapanecum* sp. nov. which has common symbiotic genes with *Sinorhizobium mexicanum*. FEMS Microbiol. Ecol. 67: 103-117

Rome, S., Fernandez, M.P., Brunel, B., Normand, P., Cleyet-Marel, J-C. (1996) *Sinorhizobium medicae* sp. nov., isolated from annual *Medicago* spp. Int. J., Syst. Bact. 46: 972-980

Sakrouhi, I., Belfquih, M., Sbabou, L., Moulin, P., Bena, G., Filali-Maltouf, A., Le Quéré, A. (2016) Recovery of symbiotic nitrogen fixing acacia rhizobia from Merzouga Desert sand dunes in South East Morocco – identification of a probable new species of *Ensifer* adapted to stressed environments. Syst. Appl. Microbiol. 39: 122-131

Tak, N., Awasthi, E., Bissa, G., Meghwal, R.R., James, E.K., Sprent, J.S., Gehlot, H.S. (2016) Multi locus sequence analysis and symbiotic characterization of novel *Ensifer* strains nodulating *Tephrosia* spp. in the Indian Thar Desert. Syst. Appl. Microbiol. doi: http://dx.doi.org/10.1016/j.syapm. 2016.08.002

Teixeira, H., Rodríguez-Echeverría, S. (2016) Identification of symbiotic nitrogen-fixing bacteria from three African leguminous trees in Gorongosa National Park. Syst. Appl. Microbiol. doi: http://dx.doi.org/10.1016/j.syapm.2016.05.004

Toledo, I., Lloret, L., Martínez-Romero, E. (2003) *Sinorhizobium americanus* sp. nov., a new *Sinorhizobium* species nodulating native *Acacia* spp. in Mexico. Syst. Appl. Microbiol. 26: 54-64

Wang, E.T., Tan, Z.Y., Willems, A., Fernández-López, M., Reinhold-Hurek, B., Martínez-Romero, E. (2002) *Sinorhizobium morelense* sp. nov., a *Leucaena* *leucocephala*-associated bacterium that is highly resistant to multiple antibiotics. Int. J. Syst. Evol. Microbiol. 52: 1687-1693

Wang, Y.C., Wang, F., Hou, B.C., Wang, E.T., Chen, W.F., Sui, X.H., Chen, W.X., Li, Y., Zhang, Y.B. (2013) Proposal of *Ensifer psoraleae* sp. nov., *Ensifer* *sesbaniae* sp. nov., *Ensifer morelense* comb. nov. and *Ensifer americanum* comb. nov. Syst. Appl. Microbiol. 36: 467-473

Wei, G.H., Wang, E.T., Tan, Z.Y., Zhu, M.E., Chen, W.X. (2002) *Rhizobium indigoferae* sp. nov. and *Sinorhizobium kummerowiae* sp. nov., respectively isolated from *Indigofera* spp. and *Kummerowia stipulacea*. Int. J. Syst. Evol. Microbiol. 52: 2231-2239

Willems, A., Collins, M.D. (1993) Phylogenetic analysis of rhizobia and agrobacteria based on 16S rRNA gene sequences. Int. J. Syst. Bacteriol. 43: 305-313

Yan, H., Yan, J., Sui, X.H., Wang, E.T., Chen, W.X., Zhang, X.X., Chen, W.F. (2016) *Ensifer glycinis* sp. nov., a rhizobial species associated with species of the genus *Glycine*. Int. J. Syst. Evol. Microbiol. 66: 2910-2916

Zhao, L., Fan, M., Zhang, D., Yang, R., Zhang, F., Xu, L., Wei, X., Shen, Y., Wei, G. (2014) Distribution and diversity of rhizobia associated with wild soybean (Glycine soja Sien. & Zucc.) in Northwest China. Syst. Appl. Microbiol. 37: 449-456
