# Supplementary material for: Both Alpha- and Beta-Rhizobia Occupy the Root Nodules of Vachellia karroo in South Africa
Source: Front Microbiol. 2019 Jun 4;10:1195. doi: 10.3389/fmicb.2019.01195 (PMC6558075; doi:10.3389/fmicb.2019.01195)
Supplement: Supplementary file 2 [file Table_2.DOCX]

**Supplementary Table S2** Isolate names, accession numbers, host/niche, country of origin and reference for the *Mesorhizobium* isolates used in this study

| **Isolate** | ***recA*** | **Host/Niche** | **Country** | **Reference** |
| --- | --- | --- | --- | --- |
| *M. abyssinicae* AC98c^T^ | GQ848011 | *Vachellia abyssinica* | Ethiopia | Degefu et al., 2013 |
| *M. albiziae* CCBAU61158^T^ | EU249396 | *Albizia kalkora* | China | Wang et al., 2007 |
| *M. alhagi* CCNWXJ12-2^T^ | FJ481878 | *Alhagi sparsifolia* | China | Chen et al., 2010 |
| *M. amorphae* ACCC19665^T^ | AM076369 | *Amorpha fruticosa* | China | Wang et al., 1999 |
| *M. australicum* WSM2073^T^ | Mesau_04438 | *Biserrula pelecinus* | Australia | Nandasena et al., 2009 |
| *M. calcicola* ICMP19560^T^ | KC237686 | *Sophora longicarinata* | New Zealand | De Meyer et al., 2016 |
| *M. camelthorni* CCNWXJ40-4^T^ | GU220798 | *Alhagi sparsifolia* | China | Chen et al., 2011 |
| *M. cantuariense* ICMP19515^T^ | KC237677 | *Sophora microphylla* | New Zealand | De Meyer et al., 2015 |
| *M. caraganae* CCBAU11299^T^ | EU249394 | *Caragana microphylla* | China | Guan et al., 2008 |
| *M. chacoense* PR5^T^ | AM076370 | *Prosopis alba* | Argentina | Velázquez et al., 2001 |
| *M. ciceri* UPM-Ca7^T^ | AJ294367 | *Cicer arietinum* | Spain | Jarvis et al., 1997 |
| *M. delmotii* STM4623^T^ | KP150570 | *Anthyllis vulneraria* | France | Mohamad et al., 2017 |
| *M. erdmanii* USDA3471^T^ | AJ294371 | *Lotus corniculatus* | New Zealand | Martínez-Hidalgo et al., 2015 |
| *M. gobiense* CCBAU83330^T^ | EF549481 | *Oxytropis glabra* | China | Han et al., 2008 |
| *M. hawassense* AC99b^T^ | GQ848025 | *Sesbania sesban* | Ethiopia | Degefu et al., 2013 |
| *M. huakuii* IFO15243^T^ | EU249391 | *Astragalus sinicus* | China | Jarvis et al., 1997 |
| *M. hungaricum* UASWS1009^T^ | QV_RS19105 | Sewage sludge from coke plant | Hungary | Crovadore et al., 2016 |
| *M. japonicum* MAFF303099^T^ | mlr0030 | *Lotus japonicum* | Japan | Martínez-Hidalgo et al., 2016 |
| *M. jarvisii* ATCC33669^T^ | KM192345 | *Lotus corniculatus* | New Zealand | Martínez-Hidalgo et al., 2015 |
| *M. kowhaii* ICMP19512^T^ | KC237674 | *Sophora microphylla* | New Zealand | De Meyer et al., 2016 |
| *M. loti* LMG6125^T^ | KM192346 | *Lotus corniculatus* | New Zealand | Jarvis et al., 1997 |
| *M. mediterraneum* LMG17148^T^ | AM182157 | *Cicer arietinum* | Spain | Jarvis et al., 1997 |
| *M. metallidurans* STM2683^T^ | AM930382 | *Anthyllis vulneraria* | France | Vidal et al., 2009 |
| *M. muleiense* CCBAU83963^T^ | HQ316782 | *Cicer arietinum* | China | Zhang et al., 2012a |
| *M. newzealandense* ICMP19545^T^ | KC237690 | *Sophora prostrata* | New Zealand | De Meyer et al., 2016 |
| *M. olivaresii* CPS13^T^ | FN556460 | *Lotus corniculatus* | Spain | Lorite et al., 2016 |
| *M. opportunistum* WSM2075^T^ | Mesop_4822 | *Biserrula pelecinus* | Australia | Nandasena et al., 2009 |
| *M. plurifarium* LMG11892^T^ | MPL1032_270146 | *Senegalia senegal* | Senegal | De Lajudie et al., 1998 |
| *M. prunaredense* STM4891^T^ | KP150569 | *Anthyllis vulneraria* | France | Mohamad et al., 2017 |
| *M. qingshengii* CCBAU33460^T^ | JQ339757 | *Astragalus sinicus* | China | Zheng et al., 2013 |
| *M. robiniae* CCNWYC115^T^ | GQ856501 | *Robinia pseudoacacia* | China | Zhou et al., 2010 |
| *M. sangaii* SCAU7^T^ | JN129442 | *Astragalus luteolus* | China | Zhou et al., 2013 |
| *M. sediminum* YIM M12096^T^ | KX151666 | Deep-sea sediment | Indian Ocean | Yuan et al., 2016 |
| *M. septentrionale* SDW014^T^ | EF639843 | *Astragalus adsurgens* | China | Gao et al., 2004 |
| *M. shangrilense* CCBAU65327^T^ | EU672501 | *Caragana bicolor* | China | Lu et al., 2009 |
| *M. shonense* AC39a^T^ | GQ848018 | *Vachellia abyssinica* | Ethiopia | Degefu et al., 2013 |
| *M. silamurunense* CCBAU01550^T^ | EU518358 | *Astragalus membranaceus* | China | Zhao et al., 2012 |
| *M. soli* NHI-8^T^ | KM188061 | *Robinia pseudoacacia* | South Korea | Nguyen et al., 2015 |
| *M. sophorae* ICMP19535^T^ | KC237704 | *Sophora microphylla* | New Zealand | De Meyer et al., 2016 |
| *M. tamadayense* Ala-3^T^ | HE608972 | *Anagyris latifolia* | Canary Islands | Ramírez-Bahena et al., 2012 |
| *M. tarimense* CCBAU83306^T^ | EF549482 | *Lotus frondosus* | China | Han et al., 2008 |
| *M. temperatum* SDW018^T^ | EF639844 | *Astragalus adsurgens* | China | Gao et al., 2004 |
| *M. thiogangeticum* SJT^T^ | AM040610 | *Clitoria ternatea* | India | Ghosh and Roy, 2006 |
| *M. tianshanense* A-1BS^T^ | EU249392 | *Glycyrrhiza pallidiflora* | China | Jarvis et al., 1997 |
| *M. waimense* ICMP19557^T^ | KC237667 | *Sophora longicarinata* | New Zealand | De Meyer et al., 2015 |
| *M. waitakense* ICMP19523^T^ | KC237693 | *Sophora microphylla* | New Zealand | De Meyer et al., 2016 |
| *M.* sp. WSM2561 | M727_RS0120015 | *Lessertia diffusa* | South Africa | Gerding et al., 2012 |
| *M.* sp. WSM3626 | M653_RS0112505 | *Lessertia diffusa* | South Africa | Gerding et al., 2012 |
| *M.* sp. ORS3356 | KJ609605 | *Vachellia seyal* | Senegal | Diouf et al., 2007 |
| *M.* sp. ORS3359 | KJ609646 | *Vachellia seyal* | Senegal | Diouf et al., 2007 |
| *M.* sp. ORS3324 | MPLB_1130024 | *Vachellia seyal* | Senegal | Diouf et al., 2007 |
| *M.* sp. ORS3369 | KJ609608 | *Vachellia seyal* | Senegal | Diouf et al., 2007 |
| *M.* sp. ORS3357 | KJ609606 | *Vachellia seyal* | Senegal | Diouf et al., 2007 |
| *M.* sp. ORS3365 | KJ609607 | *Vachellia seyal* | Senegal | Diouf et al., 2007 |
| *M.* sp. ORS3397 | KJ609609 | *Vachellia seyal* | Senegal | Diouf et al., 2007 |
| *M.* sp. MM5333 | KF802780 | *Otholobium virgatum* | South Africa | Lemaire et al., 2015 |
| *M.* sp. MM5361 | KF802756 | *Aspalathus ciliaris* | South Africa | Lemaire et al., 2015 |
| *M.* sp. MM5343 | KF802791 | *Psoralea rigidula* | South Africa | Lemaire et al., 2015 |
| *M.* sp. OD42 | KF802774 | *Otholobium bracteolatum* | South Africa | Lemaire et al., 2015 |
| *M.* sp. OD47 | KF802751 | *Argyrolobium velutinum* | South Africa | Lemaire et al., 2015 |
| *M.* sp. OD52 | KF802792 | *Psoralea brilliantissima* | South Africa | Lemaire et al., 2015 |
| *M.* sp. MM5369 | KF802778 | *Argyrolobium lunare* | South Africa | Lemaire et al., 2015 |
| *M.* sp. OD14 | KF802749 | *Argyrolobium* sp. | South Africa | Lemaire et al., 2015 |
| *M.* sp. MM5382 | KR154529 | *Otholobium hirtum* | South Africa | Lemaire et al., 2015 |
| *M.* sp. OD119 | KF802787 | *Psoralea laxa* | South Africa | Lemaire et al., 2015 |
| *M.* sp. OD26 | KF802767 | *Aspalathus uniflora* | South Africa | Lemaire et al., 2015 |
| *M.* sp. OD15 | KF802783 | *Psoralea asarina* | South Africa | Lemaire et al., 2015 |
| *M.* sp. OD18 | KF802752 | *Aspalathus astroites* | South Africa | Lemaire et al., 2015 |
| *M.* sp. MM5397 | KF802753 | *Aspalathus aurantiaca* | South Africa | Lemaire et al., 2015 |
| *M. sp*. MM5372 | KF802761 | *Aspalathus laricifolia* | South Africa | Lemaire et al., 2015 |
| *M.* sp. MM5757 | KF802762 | *Aspalathus perfoliata* | South Africa | Lemaire et al., 2015 |
| *M.* sp. MM5364 | KF802793 | *Psoralea usitata* | South Africa | Lemaire et al., 2015 |
| *M.* sp. MM5675 | KF802782 | *Otholobium zeyheri* | South Africa | Lemaire et al., 2015 |
| *M.* sp. MM5370 | KF802779 | *Otholobium* sp. | South Africa | Lemaire et al., 2015 |
| *M.* sp. MM5440 | KF802766 | *Aspalathus spicata* | South Africa | Lemaire et al., 2015 |
| *M.* sp. MM5734 | KF802768 | *Aspalathus uniflora* | South Africa | Lemaire et al., 2015 |
| *M.* sp. MM5360 | KF802784 | *Psoralea asarina* | South Africa | Lemaire et al., 2015 |
| *M.* sp. MM5336 | KF802789 | *Psoralea pinnata* | South Africa | Lemaire et al., 2015 |
| *M.* sp. MM5357 | KF802781 | *Otholobium virgatum* | South Africa | Lemaire et al., 2015 |
| *M.* sp. OD13 | KF802758 | *Aspalathus cordata* | South Africa | Lemaire et al., 2015 |
| *M.* sp. OD31 | KF802759 | *Aspalathus ericifolia* | South Africa | Lemaire et al., 2015 |
| *M.* sp. OD32 | KF802775 | *Otholobium hirtum* | South Africa | Lemaire et al., 2015 |
| *M.* sp. MM5618 | KF802754 | *Aspalathus bracteata* | South Africa | Lemaire et al., 2015 |
| *M.* sp. MM5376 | KF802777 | *Otholobium hirtum* | South Africa | Lemaire et al., 2015 |
| *M.* sp. CS13166 | KF802757 | *Aspalathus ciliaris* | South Africa | Lemaire et al., 2015 |
| *M.* sp. OD118 | KF802788 | *Psoralea oligophylla* | South Africa | Lemaire et al., 2015 |
| *M.* sp. MM5462 | KF802785 | *Psoralea congesta* | South Africa | Lemaire et al., 2015 |
| *M.* sp. MM5334 | KF802776 | *Otholobium hirtum* | South Africa | Lemaire et al., 2015 |
| *M.* sp. MM5398 | KF802765 | *Aspalathus spicata* | South Africa | Lemaire et al., 2015 |
| *M.* sp. AC100c | GQ848013 | *Senegalia senegal* | Ethiopia | Degefu et al., 2011 |
| *M.* sp. AC100e | GQ848014 | *Senegalia senegal* | Ethiopia | Degefu et al., 2011 |
| *M.* sp. AC98b | GQ848010 | *Vachellia abyssinica* | Ethiopia | Degefu et al., 2011 |
| *M.* sp. AC98a | GQ848023 | *Vachellia abyssinica* | Ethiopia | Degefu et al., 2011 |
| *M.* sp. AC98e | GQ848012 | *Vachellia abyssinica* | Ethiopia | Degefu et al., 2011 |
| *M.* sp. ORS3428 | KJ609648 | *Senegalia senegal* | Senegal | Fall et al., 2008 |
| *M.* sp. AC28c2 | GQ848016 | *Vachellia tortilis* | Ethiopia | Degefu et al., 2011 |
| *M.* sp. AC39c1 | GQ848019 | *Vachellia abyssinica* | Ethiopia | Degefu et al., 2011 |
| *M.* sp. AC39e1 | GQ848021 | *Vachellia abyssinica* | Ethiopia | Degefu et al., 2011 |
| *M.* sp. AC39e2 | GQ848022 | *Vachellia abyssinica* | Ethiopia | Degefu et al., 2011 |
| *M.* sp. AC21c2 | GQ848017 | *Vachellia tortilis* | Ethiopia | Degefu et al., 2011 |
| *M.* sp. AC21a2 | GQ848015 | *Vachellia tortilis* | Ethiopia | Degefu et al., 2011 |
| *M.* sp. ORS3573 | KJ609640 | *Senegalia senegal* | Senegal | Bakhoum et al., 2012 |
| *M.* sp. ORS3628 | KJ609649 | *Senegalia senegal* | Senegal | Bakhoum et al., 2012 |
| *M.* sp. ORS3578 | KJ609641 | *Senegalia senegal* | Senegal | Bakhoum et al., unpubl. |
| *M.* sp. ORS3416 | KJ609632 | *Senegalia senegal* | Senegal | Fall et al., 2008 |
| *M. sp*. ORS3600 | KJ609617 | *Senegalia senegal* | Senegal | Bakhoum et al., 2012 |
| *M.* sp. ORS3610 | KJ609618 | *Senegalia senegal* | Senegal | Bakhoum et al., 2012 |
| *M.* sp. ORS3598 | KJ609616 | *Senegalia senegal* | Senegal | Bakhoum et al., unpubl. |
| *M.* sp. ORS3596 | KJ609615 | *Senegalia senegal* | Senegal | Bakhoum et al., unpubl. |
| *M.* sp. ORS3593 | KJ609614 | *Senegalia senegal* | Senegal | Bakhoum et al., 2012 |
| *M.* sp. ORS3588 | KJ609613 | *Senegalia senegal* | Senegal | Bakhoum et al., unpubl. |
| *M.* sp. 3C | LN890785 | *Vachellia karroo* | South Africa | This study |
| *M.* sp. 4C | LN890786 | *Vachellia karroo* | South Africa | This study |
| *M.* sp. 21C | LN890787 | *Vachellia karroo* | South Africa | This study |
| *M.* sp. 21E | LN890788 | *Vachellia karroo* | South Africa | This study |
| *M.* sp. 22B | LN890789 | *Vachellia karroo* | South Africa | This study |
| *M.* sp. 22C | LN890790 | *Vachellia karroo* | South Africa | This study |
| *M.* sp. 22E | LN890791 | *Vachellia karroo* | South Africa | This study |
| *M.* sp. 23E | LN890792 | *Vachellia karroo* | South Africa | This study |
| *M.* sp. 25D | LN890793 | *Vachellia karroo* | South Africa | This study |
| *M.* sp. 25B | LN890794 | *Vachellia karroo* | South Africa | This study |
| *M.* sp. 25A | LN890795 | *Vachellia karroo* | South Africa | This study |
| *M.* sp. 4A | LN890796 | *Vachellia karroo* | South Africa | This study |
| *M.* sp. 1D | LN890797 | *Vachellia karroo* | South Africa | This study |
| *M.* sp. 4B | LN890798 | *Vachellia karroo* | South Africa | This study |
| *M.* sp. 23D | LN890799 | *Vachellia karroo* | South Africa | This study |
| *M.* sp. 23A | LN890800 | *Vachellia karroo* | South Africa | This study |
| *M.* sp. 24D | LN890801 | *Vachellia karroo* | South Africa | This study |
| *M.* sp. 24A | LN890802 | *Vachellia karroo* | South Africa | This study |
| *M.* sp. 23B | LN890803 | *Vachellia karroo* | South Africa | This study |
| *M.* sp. 2C | LN890804 | *Vachellia karroo* | South Africa | This study |
| *M.* sp. 2B | LN890805 | *Vachellia karroo* | South Africa | This study |
| *M.* sp. 2D | LN890806 | *Vachellia karroo* | South Africa | This study |
| *M.* sp. 9D | LN890807 | *Vachellia karroo* | South Africa | This study |
| *M.* sp. 9B | LN890808 | *Vachellia karroo* | South Africa | This study |
| *M.* sp. 3E | LN890809 | *Vachellia karroo* | South Africa | This study |
| *M.* sp. 3D | LN890810 | *Vachellia karroo* | South Africa | This study |
| *M.* sp. DJ16 | KJ609621 | *Senegalia senegal* | Senegal | Diouf et al., 2015 |
| *M.* sp. ORS3400 | KJ609611 | *Vachellia seyal* | Senegal | Diouf et al., 2007 |
| *M.* sp. DJ17 | KJ609622 | *Senegalia senegal* | Senegal | Diouf et al., 2015 |
| *M.* sp. DJ20 | KJ609623 | *Senegalia senegal* | Senegal | Diouf et al., 2015 |
| *M.* sp. SD11 | KJ609625 | *Senegalia senegal* | Senegal | Diouf et al., 2015 |
| *M.* sp. SD4 | KJ609624 | *Senegalia senegal* | Senegal | Diouf et al., 2015 |
| *M.* sp. ORS3399 | KJ609610 | *Vachellia seyal* | Senegal | Diouf et al., 2007 |
| *M.* sp. DA8 | KJ609628 | *Senegalia senegal* | Senegal | Diouf et al., 2015 |
| *M.* sp. ORS3302 | KJ609604 | *Vachellia seyal* | Senegal | Diouf et al., 2007 |
| *M.* sp. TCH9 | KJ609630 | *Senegalia senegal* | Senegal | Diouf et al., 2015 |
| *M.* sp. TCH17 | KJ609631 | *Senegalia senegal* | Senegal | Diouf et al., 2015 |
| *M.* sp. K16 | KJ609619 | *Senegalia senegal* | Senegal | Diouf et al., 2015 |
| *M.* sp. KA2 | KJ609629 | *Senegalia senegal* | Senegal | Diouf et al., 2015 |
| *M.* sp. SOD14 | KJ609626 | *Senegalia senegal* | Senegal | Diouf et al., 2015 |
| *M.* sp. ND20 | KJ609627 | *Senegalia senegal* | Senegal | Diouf et al., 2015 |
| *M.* sp. B17 | KJ609643 | *Senegalia senegal* | Senegal | Diouf et al., 2015 |
| *M.* sp. ORS3448 | KJ609637 | *Senegalia senegal* | Senegal | Fall et al., 2008 |
| *M.* sp. ORS3450 | KJ609638 | *Senegalia senegal* | Senegal | Fall et al., 2008 |
| *M.* sp. DJ14 | KJ609642 | *Senegalia senegal* | Senegal | Diouf et al., 2015 |
| *M.* sp. ORS3437 | KJ609634 | *Senegalia senegal* | Senegal | Fall et al., 2008 |
| *M.* sp. ORS3452 | KJ609639 | *Senegalia senegal* | Senegal | Fall et al., 2008 |
| *M.* sp. ORS3447 | KJ609636 | *Senegalia senegal* | Senegal | Fall et al., 2008 |
| *M.* sp. SOD15 | KJ609645 | *Senegalia senegal* | Senegal | Diouf et al., 2015 |
| *M.* sp. ORS3423 | KJ609633 | *Senegalia senegal* | Senegal | Fall et al., 2008 |
| *M.* sp. ORS3443 | KJ609635 | *Senegalia senegal* | Senegal | Fall et al., 2008 |
| *M.* sp. SOD10 | KJ609644 | *Senegalia senegal* | Senegal | Diouf et al., 2015 |
| *M.* sp. MM5413 | KF802790 | *Psoralea pullata* | South Africa | Lemaire et al., 2015 |
| *M.* sp. MM5352 | KF802760 | *Aspalathus ericifolia* | South Africa | Lemaire et al., 2015 |
| *M.* sp. OD48 | KF802750 | *Argyrolobium lunare* | South Africa | Lemaire et al., 2015 |
| *M.* sp. Gs663 | KU041615 | *Genista saharae* | Algeria | Chaich, unpubl. |
| *M.* sp. AN11 | JN089682 | *Vachellia tortilis* | Tunisia | Fterich et al., 2012 |
| *M.* sp. X874D | KR232261 | *Vachellia xanthoploea* | Mozambique | Teixeira and Rodríguez-Echeverría, 2016 |
| *M.* sp. X96B1 | KR232266 | *Vachellia xanthoploea* | Mozambique | Teixeira and Rodríguez-Echeverría, 2016 |

**References**

Bakhoum, N., Ndoye, F., Kane, A., Assigbetse, K., Fall, D.,Sylla, S.N., Noba, K., Diouf, D. (2012) Impact of rhizobial inoculation on *Acacia senegal* (L.) Willd. growth in greenhouse and soil functioning in relation to seed provenance and soil origin. World J. Microbiol. Biotechnol. 28: 2567-2579

Chen, W-M., Zhu, W-F., Bontemps, C., Young, J.P.W., Wei, G.H. (2010) *Mesorhizobium alhagi* sp. nov., isolated from wild *Alhagi sparsifolia* in north-western China. Int. J. Syst. Evol. Microbiol. 60: 958-962

Chen, W-M., Zhu, W-F., Bontemps, C., Young, J.P.W., Wei, G-H. (2011) *Mesorhizobium camelthorni* sp. nov., isolated from *Alhagi sparsifolia*. Int. J. Syst. Evol. Microbiol. 61: 574-579

Crovadore, J., Cochard, B., Calmin, G., Chablais, R., Schulz, T., Lefort, F. (2016) Whole-genome sequence of *Mesorhizobium hungaricum* sp. nov. strain UASWS1009, a potential resource for agricultural and environmental uses. Genome Announcements 4(5): e01158-16 doi: 10.1128/genomeA.01158-16

Diouf, D., Samba-Mbaye, R., Lesueur, D., Ba, A.T., Dreyfus, B., de Lajudie, P., Neyra, M. (2007) Genetic diversity of *Acacia syeal* Del. rhizobial populations indigenous to Senegalese soils in relation to salinity and pH of the sampling sites. Microbial Ecology 54: 553-566

Degefu, T., Wolde-Meskel, E., Liu, B., Cleenwerck, I., Willems, A., Frostegård, Å. (2013) *Mesorhizobium shonense* sp. nov., *Mesorhizobium hawassense* sp. nov. and *Mesorhizobium abyssinicae* sp. nov., isolated from root nodules of different agroforestry legume trees. Int. J. Syst. Evol. Microbiol. 63: 1746-1753.

Degefu, T., Wolde-Meskel, E., Frostegård, Å. (2011) Multilocus sequence analyses reveal several unnamed *Mesorhizobium* genospecies nodulating *Acacia* species and *Sesbania sesban* trees in Southern regions of Ethiopia. Syst. Appl. Microbiol. 34: 216-226

De Lajudie, P., Willems, A., Nick, G., Moreira, F., Molouba, F., Hoste, B., Torck, U. Neyra, M., Collins, M.D., Lindström, K., Dreyfus, B., Gillis, M. (1998) Characterization of tropical tree rhizobia and description of *Mesorhizobium plurifarium* sp. nov. Int. J. Syst. Bacteriol. 48: 369-382

De Meyer, S.E., Tan, H.W., Andrews, M., Heenan, P.B., Willems, A. (2016) *Mesorhizobium calcicola* sp. nov., *Mesorhizobium waitakense* sp. nov., *Mesorhizobium sophorae* sp. nov., *Mesorhizobium newzealandense* sp. nov. and *Mesorhizobium kowhaii* sp. nov. isolated from *Sophora* root nodules. Int. J. Syst. Evol. Microbiol. 66: 786-795

De Meyer, S.E., Tan, H.W., Heenan, P.B., Andrews, M., Willems, A. (2015) *Mesorhizobium waimense* sp. nov. isolated from *Sophora longicarinata* root nodules and *Mesorhizobium cantuariense* sp. nov. isolated from *Sophora microphylla* root nodules. Int. J. Syst. Evol. Microbiol. 65: 3419-3426

Diouf, F., Diouf, D., Klonowska, A., Le Queré, A., Bakhoum, N., Fall, D., Neyra, M., Parrinello, H., Diouf, M., Ndoye, I., Moulin, L. (2015) Genetic and genomic diversity studies of *Acacia* symbionts in Senegal reveal new species of *Mesorhizobium* with a putative geographical pattern. PLoS ONE 10(2): e0117667 doi: 10.1371/journal.pone.0117667

Fall, D., Diouf, D., Ourarhi, M., Faye, A., Abdelmounen, H., Neyra, M., Sylla, S.N., Missbah El Idrissi, M. (2008) Phenotypic and genotypic characteristics of *Acacia senegal* (L.) Willd. root-nodulating bacteria isolated from soils in the dryland part of Senegal. Letters in Applied Microbiology 47: 85-97

Fterich, A., Mahdhi, M., Lafuente, A., Pajuelo, E., Caviedes, M.A., Rodriguez-Llorente, I.D., Mars, M. (2012) Taxonomic and symbiotic diversity of bacteria isolated from nodules of *Acacia tortilis* subsp. *raddiana* in arid soils of Tunisia. Can. J. Microbiol. 58: 738-751

Gao, J-L., Turner, S.L., Kan, F.L., Wang, E.T., Tan, Z.Y., Qiu, Y.H., Gu, J., Terefework, Z., Young, J.P.W., Lindström, K., Chen, W.X. (2004) *Mesorhizobium septentrionale* sp. nov. and *Mesorhizobium temperatum* sp. nov., isolated from *Astragalus adsurgens* growing in the northern regions of China. Int. J. Syst. Evol. Microbiol. 54: 2003-2012

Gerding, M., O’Hara, G.W., Bräu, L., Nandasena, K., Howieson, J.G. (2012) Diverse *Mesorhizobium* spp. with unique *nodA* nodulating the South African legume species of the genus *Lessertia*. Plant Soil 358: 385-401

Ghosh, W., Roy, P. (2006) *Mesorhizobium thiogangeticum* sp. nov., a novel sulfur-oxidizing chemolithoautotrophy from rhizosphere soil of an Indian tropical leguminous plant. Int. J. Syst. Evol. Microbiol. 56: 91-97

Guan, S.H., Chen, W.F., Wang, E.T., Lu, Y.L., Yan, X.R., Zhang, X.X., Chen, W.X. (2008) *Mesorhizobium caraganae* sp. nov., a novel rhizobial species nodulated with *Caragana* spp. in China. Int. J. Syst. Evol. Microbiol. 58: 2646-2653

Han, T.X., Han, L.L., Wu, L.J., Chen, W.F., Sui, X.H., Gu, J.G., Wang, E.T., Chen, W.X. (2008) *Mesorhizobium gobiense* sp. nov. and *Mesorhizobium tarimense* sp. nov., isolated from wild legumes growing in desert soils of Xinjiang, China. Int. J. Syst. Evol. Microbiol. 58: 2610-2618

Jarvis, B.D.W., van Berkum, P., Chen, W.X., Nour, S.M., Fernandez, M.P., Cleyet-Marel, J.C., Gillis, M. (1997) Transfer of *Rhizobium loti*, *Rhizobium huakuii*, *Rhizobium ciceri*, *Rhizobium mediterraneum*, and *Rhizobium tianshanense* to *Mesorhizobium* gen. nov. Int. J. Syst. Bacteriol. 47: 895-898

Lemaire, B., Dlodlo, O., Chimphango, S., Stirton, C., Schrire, B., Boatwright, J.S., Honnay, O., Smets, E., Sprent, J., James, E.K., Muasya, A.M. (2015a) Symbiotic diversity, specificity and distribution of rhizobia in native legumes of the Core Cape Subregion (South Africa). FEMS Microbiology Ecology 91. Doi: 10.1093/femsec/fiu024

Lorite, M.J., Flores-Félix, J.D., Peix, Á., Sanjuán, J., Velázquez, E. (2016) *Mesorhizobium olivaresii* sp. nov., isolated from *Lotus corniculatus* nodules. Syst. Appl. Microbiol. 39: 557-561

Lu, Y.L., Chen, W.F., Wang, E.T., Han, L.L., Zhang, X.X., Chen, W.X., Han, S.Z. (2009) *Mesorhizobium shangrilense* sp. nov., isolated from root nodules of *Caragana* species. Int. J. Syst. Evol. Microbiol. 59: 3012-3018

Martínez-Hidalgo, P., Ramírez-Bahena, M.H., Flores-Félix, J.D., Rivas, R., Igual, J.M., Mateos, P.F., Martínez-Molina, E., León-Barrios, M., Peix, Á., Velázquez, E. (2015) Revision of the taxonomic status of type strains of *Mesorhizobium loti* and reclassification of strain USDA 3471^T^ as the type strain of *Mesorhizobium erdmanii* sp. nov. and ATCC 33669^T^ as the type strain of *Mesorhizobium jarvisii* sp. nov. Int. J. Syst. Evol. Microbiol. 65: 1703-1708

Mohamad, R., Willems, A., Le Quéré, A., Maynaud, G., Pervent, M., Bonabaud, M., Dubois, E., Cleyet-Marel, J-C., Brunel, B. (2017) *Mesorhizobium delmotii* and *Mesorhizobium prunaredense* are two new species containing rhizobial strains within the symbiovar anthyllidis. Syst. Appl. Microbiol. 40: 135-143

Nandasena, K.G., O’Hara, G.W., Tiwari, R.P., Willems, A., Howieson, J.G. (2009) *Mesorhizobium australicum* sp. nov. and *Mesorhizobium opportunistum* sp. nov., isolated from *Biserrula pelecinus* L. in Australia. Int. J. Syst. Evol. Microbiol. 59: 2140-2147

Nguyen, T.M., Pham, V.H.T., Kim, J. (2015) *Mesorhizobium soli* sp. nov., a novel species isolated from the rhizosphere of *Robinia pseudoacacia* L. in South Korea by using a modified culture method. Antonie van Leeuwenhoek 108: 301-310

Ramírez-Bahena, M.H., Hernández, M., Peix, Á., Velázquez, E., León-Barrios, M. (2012) Mesorhizobial strains nodulating *Anagyris latifolia* and *Lotus berthelotii* in Tamadaya ravine (Tenerife, Canary Islands) are two symbiovars of the same species, *Mesorhizobium tamadayense* sp. nov. Syst. Appl. Microbiol. 35: 334-341

Velázquez, E., Igual, J.M., Willems, A., Fernández, M.P., Muñoz, E., Mateos, P.F., Abril, A., Toro, N., Normand, P., Cervantes, E., Gillis, M., Martínez-Molina, E. (2001) *Mesorhizobium chacoense* sp. nov., a novel species that nodulates *Prosopis alba* in the Chaco Arido region (Argentina). Int. J. Syst. Evol. Microbiol. 51: 1011-1021

Vidal, C., Chantreuil, C., Berge, O., Mauré, L., Escarré, J., Béna, G., Brunel, B., Cleyet-Marel, J-C. (2009) *Mesorhizobium metallidurans* sp. nov., a metal-resistant symbiont of *Anthyllis vulneraria* growing on metallicolous soil in Languedoc, France. Int. J. Syst. Evol. Microbiol. 59: 850-855

Wang, E.T., van Berkum, P., Sui, X.H., Beyene, D., Chen, W.X., Martínez-Romero, E. (1999) Diversity of rhizobia associated with *Amorpha fruticosa* isolated from Chinese soils and description of *Mesorhizobium amorphae* sp. nov. Int. J. Syst. Bacteriol. 49: 51-65

Wang, F.Q., Wang, E.T., Liu, J., Chen, Q., Sui, X.H., Chen, W.F., Chen, W.X. (2007) *Mesorhizobium albiziae* sp. nov., a novel bacterium that nodulates *Albizia kalkora* in a subtropical region of China. Int. J. Syst. Evol. Microbiol. 57: 1192-1199

Yuan, C-G., Jiang, Z., Xiao, M., Zhou, E-M., Kim, C-J., Hozzein, W.N., Park, D-J., Zhi, X-Y., Li, W-J. (2016) *Mesorhizobium sediminum* sp. nov., isolated from deep-sea sediment. Int. J. Syst. Evol. Microbiol. 66: 4797-4802

Zhang, J.J., Liu, T.Y., Chen, W.F., Wang, E.T., Sui, X.H., Zhang, X.X., Li, Y., Li, Y., Chen, W.X. (2012a) *Mesorhizobium muleiense* sp. nov., nodulating with *Cicer* *arietinum* L. Int. J. Syst. Evol. Microbiol. 62: 2737-2742

Zhao, C.T., Wang, E.T., Zhang, Y.M., Chen, W.F., Sui, X.H., Chen, W.X., Liu, H.C., Zhang, X.X. (2012) *Mesorhizobium silamurunense* sp. nov., isolated from root nodules of *Astragalus* species. Int. J. Syst. Evol. Microbiol. 62: 2180-2186

Zheng, W.T., Li, Jr. Y., Wang, R., Sui, X.H., Zhang, X.X., Zhang, J.J., Wang, E.T., Chen, W.X. (2013) *Mesorhizobium qingshengii* sp. nov., isolated from effective nodules of Astragalus sinicus. Int. J. Syst. Evol. Microbiol. 63: 2002-2007

Zhou, P.F., Chen, W.M., Wei, G.H. (2010) *Mesorhizobium robiniae* sp. nov., isolated from root nodules of *Robinia pseudoacacia*. Int. J. Syst. Evol. Microbiol. 60: 2552-2556

Zhou, S., Li, Q., Jiang, H., Lindström, K., Zhang, X. (2013) *Mesorhizobium* *sangaii* sp. nov., isolated from the root nodules of *Astragalus luteolus* and *Astragalus* *ernestii*. Int. J. Syst. Evol. Microbiol. 63: 2794-2799
