# Supplementary material for: Both Alpha- and Beta-Rhizobia Occupy the Root Nodules of Vachellia karroo in South Africa
Source: Front Microbiol. 2019 Jun 4;10:1195. doi: 10.3389/fmicb.2019.01195 (PMC6558075; doi:10.3389/fmicb.2019.01195)
Supplement: Supplementary file 3 [file Table_3.DOCX]

**Supplementary Table S3** Isolate names, accession numbers, host/niche, country of origin and references for the *Paraburkholderia* isolates used in this study

| **Isolate** | ***recA*** | **Host/Niche** | **Country** | **Reference** |
| --- | --- | --- | --- | --- |
| *P. acidipaludis* SA33^T^/NBRC101816 | BAC01S_RS07915 | *Eleocharis dulcis* | Vietnam | Aizawa et al., 2010a |
| *P. aspalathi* VG1C^T^ | KF356197 | *Aspalathus abietina* | South Africa | Mavengere et al., 2014 |
| *P. bannensis* E25^T^/NBRC103871^T^ | BBA01S_RS19170 | *Panicum repens* | Thailand | Aizawa et al., 2011 |
| *P. bryophila* LMG 23644^T^ | HQ398574 | *Sphagnum rubellum* | Germany | Vandamme et al., 2007 |
| *P. caballeronis* TNe-841^T^/LMG26416^T^ | LT708247 | *Lycopersicon esculentum* | Mexico | Martínez-Aguilar et al., 2013 |
| *P. caffeinilytica* CF1^T^ | KU723583 | Tea plantation soil | China | Gao et al., 2016a |
| *P. caffeinitolerans* CF3^T^ | KU180360 | Tea plantation soil | China | Gao et al., 2016b |
| *P. caledonica* LMG 19076^T^ | HQ398575 | Rhizosphere | UK | Coenye et al., 2001 |
| *P. caribensis* MWAP64^T^ | HQ398576 | Vertisol microaggregates | Martinique | Achouak et al., 1999 |
| *P. caryophylli* ATCC25418^T^ | HQ398562 | *Dianthus caryophyllus* | USA | Yabuuchi et al., 1992 |
| *P. diazotrophica* JPY461^T^ | FN543898 | *Mimosa candollei* | Brazil | Sheu et al., 2013 |
| *P. dilworthii* WSM3556^T^ | F759_RS0112740 | *Lebeckia ambigua* | South Africa | De Meyer et al., 2014 |
| *P. dipogonis* DL7^T^ | JX009159 | *Dipogon lignosus* | New Zealand | Sheu et al., 2015a |
| *P. ferrariae* FeGl01^T^ | HQ398577 | Iron Ore | Brazil | Valverde et al., 2006 |
| *P. fungorum* LMG 16225^T^ | AY619664 | *Phanerochaete chrysosporium* | France | Coenye et al., 2001 |
| *P. ginsengisoli* KMY03^T^/NBRC100965^T^ | BGI01S_RS00920 | Ginseng field soil | South Korea | Kim et al., 2006 |
| *P. ginsengiterrae* DCY85^T^ | KM495734 | Ginseng rhizosphere | Republic of Korea | El-Agamy Farh et al., 2015 |
| *P. graminis* C4D1M^T^ | AY619653 | Maize root system | France | Viallard et al., 1998 |
| *P. heleia* SA41^T^/NBRC101817^T^ | BHE01S_RS18150 | *Eleocharis dulcis* | Vietnam | Aizawa et al., 2010b |
| *P. hiiakae* I2^T^ | KY305131 | Soil from Kilauea Volcano | Hawai’i, USA | Weber and King, 2017 |
| *P. hospita* LMG 20598^T^ | FJ958192 | Agricultural soil | Belgium | Goris et al., 2002 |
| *P. humisilvae* Y-12^T^ | LC036348 | Forest soil | Korea | Lee and Whang, 2015 |
| *P. kirstenboschensis* Kb15^T^ | HF544403 | *Virgilia oroboides* | South Africa | Steenkamp et al., 2015 |
| *P. kururiensis* KP23^T^/LMG19447T | AY619654 | Aquifer polluted with TCE | Japan | Zhang et al., 2000 |
| *P. megapolitana* LMG 23650^T^ | HQ398583 | *Aulacomnium palustre* | Germany | Vandamme et al., 2007 |
| *P. metrosideri* DNBP6-1^T^ | KY305132 | Soil from Kilauea Volcano | Hawai’i, USA | Weber and King, 2017 |
| *P. mimosarum* PAS44^T^/LMG23256^T^ | A19U_RS0115950 | *Mimosa pigra* | Taiwan | Chen et al., 2006 |
| *P. monticola* JC2948^T^ | CI15_03980 | Gwanak Mountain soil | Republic of Korea | Baek et al., 2015 |
| *P. nodosa* Br3437^T^ | EU294398 | *Mimosa scabrella* | Brazil | Chen et al., 2007 |
| *P. oxyphila* OX-01^T^/NBRC105797^T^ | BO1_RS12465 | Acidic forest soil | Japan | Otsuka et al., 2011 |
| *P. pallidirosea* DHOK13^T^ | KR092136 | Monsoon evergreen broad-leaved forest soil | China | Lv et al., 2016 |
| *P. paradisi* WA^T^ | KY305130 | Soil from Kilauea Volcano | Hawai’i, USA | Weber and King, 2017 |
| *P. peleae* PP52-1^T^ | KY305133 | Soil from Kilauea Volcano | Hawai’i, USA | Weber and King, 2017 |
| *P. piptadeniae* STM7183^T^ | LN875227 | *Piptadenia gonoacantha* | Brazil | Bournaud et al., 2017 |
| *P. phenazinium* LMG 2247^T^ | HQ398588 | Threonine enriched soil | - | Viallard et al., 1998 |
| *P. phenoliruptrix* AC1100^T^ | HQ398589 | Chemostat | - | Coenye et al., 2004 |
| *P. phymatum* STM815^T^/LMG21445^T^ | AY644640 | *Machaerium lunatum* | French Guiana | Vandamme et al., 2002 |
| *P. phytofirmans* PsJN^T^ | BPHYT_RS16360 | Onion roots | Canada | Sessitsch et al., 2005 |
| *P. ribeironis* STM7296^T^ | BN2475_470030 | *Piptadenia gonoacantha* | Brazil | Bournaud et al., 2017 |
| *P. rinojensis* A396^T^ | KF650992 | Soil | Japan | Cardova-Kreylos et al., 2013 |
| *P. rhizosphaerae* WR43^T^ | LC036350 | Rhizosphere soil | Korea | Lee and Whang, 2015 |
| *P. rhizoxinica* HKI454^T^ | RBRH_01977 | *Rhizopus microsporus* | Germany | Partida-Martinez et al., 2007 |
| *P. rhynchosiae* WSM3937^T^ | HE994064 | *Rhynchosia ferulifolia* | South Africa | De Meyer et al., 2013b |
| *P. sabiae* Br3407^T^/LMG24235^T^ | HQ398591 | *Mimosa caesalpiniifolia* | Brazil | Chen et al., 2008 |
| *P. sacchari* IPT101^T^/LMG19450^T^ | AY644641 | Sugarcane plantation soil | Brazil | Brämer et al., 2001 |
| *P. sartisoli* RP007^T^/LMG24000^T^ | HQ398593 | PAH-contaminated soil | New Zealand | Vanlaere et al., 2008 |
| *P. sediminicola*  HU2-65W^T^/LMG24238^T^ | HQ398594 | Freshwater sediment | Republic of Korea | Lim et al., 2008 |
| *P. silvatlantica* SRMrh-20^T^/LMG23149^T^ | HQ849157 | Maize rhizosphere | Brazil | Perin et al., 2006 |
| *P. soli* GP25-8^T^/LMG24076^T^ | HQ398597 | Soil cultivated with ginseng | Korea | Yoo et al., 2007 |
| *P. solisilvae* Y-47^T^ | LC036349 | Forest soil | Korea | Lee and Whang, 2015 |
| *P. sprentiae* WSM5005^T^ | HE994077 | *Lebeckia ambigua* | South Africa | De Meyer et al., 2013a |
| *P. susongensis* L226^T^ | KM246785 | Weathered rock surface | China | Gu et al., 2015 |
| *P. symbiotica* JPY345^T^ | FN543850 | *Mimosa cordistipula* | Brazil | Sheu et al., 2012 |
| *P. terrae* KMY02^T^/NBRC100964^T^ | BTE01S_RS25325 | Forest soil | South Korea | Yang et al., 2006 |
| *P. terricola* LMG20594^T^ | HQ398599 | Agricultural soil | Belgium | Goris et al., 2002 |
| *P. tropica* Ppe8^T^/LMG22274^T^ | HQ849161 | Endophyte of sugarcane | Brazil | Reis et al., 2004 |
| *P. tuberum* STM678^T^/LMG21444^T^ | AY619674 | *Aspalathus carnosa* | South Africa | Vandamme et al., 2002 |
| *P. ultramafica* STM10279^T^ | LC001803 | *Costularia* (*Cyperaceae*) roots | New Caledonia | Guentas et al., 2016 |
| *P. unamae* MTl-641^T^ | DQ514539 | Maize rhizosphere | Mexico | Caballero-Mellado et al., 2004 |
| *P. xenovorans* LB400^T^ | DR64_RS13995 | PCB-contaminated soil | USA | Goris et al., 2004 |
| *P.* sp. UCT34 | HF544418 | *Cyclopia glabra* | South Africa | Beukes et al., 2013 |
| *P.* sp. UCT43 | HF544427 | *Cyclopia meyeriana* | South Africa | Beukes et al., 2013 |
| *P.* sp. UCT56 | HF544428 | *Cyclopia meyeriana* | South Africa | Beukes et al., 2013 |
| *P.* sp. CB2 | HF544415 | *Cyclopia buxifolia* | South Africa | Beukes et al., 2013 |
| *P.* sp. RAU2l | HF544379 | *Hypocalyptus coluteoides* | South Africa | Beukes et al., 2013 |
| *P.* sp. RAU2f | HF544373 | *Hypocalyptus coluteoides* | South Africa | Beukes et al., 2013 |
| *P.* sp. RAU2k | HF544378 | *Hypocalyptus coluteoides* | South Africa | Beukes et al., 2013 |
| *P.* sp. RAU2h | HF544375 | *Hypocalyptus coluteoides* | South Africa | Beukes et al., 2013 |
| *P.* sp. Kb12 | HF544400 | *Virgilia oroboides* | South Africa | Beukes et al., 2013 |
| *P.* sp. CM1 | HF544425 | *Cyclopia maculata* | South Africa | Beukes et al., 2013 |
| P. sp. OD120 | KF791814 | *Crotalaria* sp. | South Africa | Lemaire et al., 2016 |
| *P.* sp. RAU2c | HF544370 | *Hypocalyptus coluteoides* | South Africa | Beukes et al., 2013 |
| *P.* sp. RAU2g | HF544374 | *Hypocalyptus coluteoides* | South Africa | Beukes et al., 2013 |
| *P.* sp. RAU2d | HF544371 | *Hypocalyptus coluteoides* | South Africa | Beukes et al., 2013 |
| *P.* sp. WC7.3c | HF544393 | *Podalyria calyptrata* | South Africa | Beukes et al., 2013 |
| *P. sp*. WC7.3f | HF544395 | *Podalyria calyptrata* | South Africa | Beukes et al., 2013 |
| *P.* sp. Cses4 | HF544430 | *Cyclopia sessiliflora* | South Africa | Beukes et al., 2013 |
| *P.* sp. CI1 | HF544420 | *Cyclopia intermedia* | South Africa | Beukes et al., 2013 |
| *P.* sp. Clong1 | HF544423 | *Cyclopia longifolia* | South Africa | Beukes et al., 2013 |
| *P.* sp. Clong3 | HF544424 | *Cyclopia longifolia* | South Africa | Beukes et al., 2013 |
| *P.* sp. OD25-R1 | KF791830 | *Podalyria calyptrata* | South Africa | Lemaire et al., 2016 |
| *P.* sp. RAU2i | HF544376 | *Hypocalyptus coluteoides* | South Africa | Beukes et al., 2013 |
| *P.* sp. MM5746 | KF791821 | *Indigofera* sp. | South Africa | Lemaire, unpubl. |
| *P.* sp. OD29 | KF791804 | *Bolusafra bituminosa* | South Africa | Lemaire et al., 2016 |
| *P.* sp. Kb6 | HF544399 | *Virgilia oroboides* | South Africa | Beukes et al., 2013 |
| *P.* sp. OD22 | KF791851 | *Rafnia acuminata* | South Africa | Lemaire et al., 2016 |
| *P.* MM5366-R1 | KF791862 | *Virgilia oroboides* | South Africa | Lemaire et al., 2016 |
| *P.* sp. OD28-R1 | KF791855 | *Rafnia* sp. | South Africa | Lemaire et al., 2016 |
| *P.* sp. MM5337-R1 | KF791834 | *Podalyria calyptrata* | South Africa | Lemaire et al., 2016 |
| *P.* sp. WC7.3a | HF544391 | *Podalyria calyptrata* | South Africa | Beukes et al., 2013 |
| *P.* sp. UCT71 | HF544419 | *Cyclopia glabra* | South Africa | Beukes et al., 2013 |
| *P.* sp. CS2 | HF544433 | *Cyclopia subternata* | South Africa | Beukes et al., 2013 |
| *P.* sp. WK1.1h | HF544386 | *Hypocalyptus sophoroides* | South Africa | Beukes et al., 2013 |
| *P.* sp. WK1.1i | HF544387 | *Hypocalyptus sophoroides* | South Africa | Beukes et al., 2013 |
| *P.* sp. WK1.1d | HF544382 | *Hypocalyptus sophoroides* | South Africa | Beukes et al., 2013 |
| *P.* sp. WK1.1f | HF544384 | *Hypocalyptus sophoroides* | South Africa | Beukes et al., 2013 |
| *P.* sp. WK1.1g | HF544385 | *Hypocalyptus sophoroides* | South Africa | Beukes et al., 2013 |
| *P.* sp. WK1.1a | HF544380 | *Hypocalyptus sophoroides* | South Africa | Beukes et al., 2013 |
| *P. s*p. WK1.1j | HF544388 | *Hypocalyptus sophoroides* | South Africa | Beukes et al., 2013 |
| *P.* sp. WK1.1k | HF544389 | *Hypocalyptus sophoroides* | South Africa | Beukes et al., 2013 |
| *P. sp*. WK1.1m | HF544390 | *Hypocalyptus sophoroides* | South Africa | Beukes et al., 2013 |
| *P.* sp. HC1.1bh | HF544413 | *Hypocalyptus sophoroides* | South Africa | Beukes et al., 2013 |
| *P.* sp. WSM4182 | HE994072 | *Lebeckia ambigua* | South Africa | Howieson et al., 2013 |
| *P.* sp. WSM4181 | HE994071 | *Lebeckia ambigua* | South Africa | Howieson et al., 2013 |
| *P.* sp. WSM4178 | HE994069 | *Lebeckia ambigua* | South Africa | Howieson et al., 2013 |
| *P.* sp. RAU6.4b | HF544368 | *Hypocalyptus oxalidifolius* | South Africa | Beukes et al., 2013 |
| *P.* sp. MM5875 | KF791845 | *Podalyria burchellii* | South Africa | Lemaire et al., 2015a |
| *P.* sp. MM5878 | KF791819 | *Indigofera angustifolius* | South Africa | Lemaire et al., 2015a |
| *P.* sp. RAU2j | HF544377 | *Hypocalyptus coluteoides* | South Africa | Beukes et al., 2013 |
| *P.* sp. UCT30 | HF544431 | *Cyclopia sessiliflora* | South Africa | Beukes et al., 2013 |
| *P.* sp. HC1.1a1 | HF544405 | *Hypocalyptus sophoroides* | South Africa | Beukes et al., 2013 |
| *P.* sp. CI2 | HF544421 | *Cyclopia intermedia* | South Africa | Beukes et al., 2013 |
| *P.* sp. CI3 | HF544422 | *Cyclopia intermedia* | South Africa | Beukes et al., 2013 |
| *P.* sp. UCT15 | HF544417 | *Cyclopia genistoides* | South Africa | Beukes et al., 2013 |
| *P.* sp. Cpub6 | HF544429 | *Cyclopia pubescens* | South Africa | Beukes et al., 2013 |
| *P.* sp. Kb1A | HF544397 | *Virgilia oroboides* | South Africa | Beukes et al., 2013 |
| *P.* sp. WK1.1e | HF544383 | *Hypocalyptus sophoroides* | South Africa | Beukes et al., 2013 |
| *P. sp*. UCT2 | HF544416 | *Cyclopia genistoides* | South Africa | Beukes et al., 2013 |
| *P.* sp. UCT31 | HF544432 | *Cyclopia sessiliflora* | South Africa | Beukes et al., 2013 |
| *P.* sp. HC6.4b | HF544414 | *Hypocalyptus oxalidifolius* | South Africa | Beukes et al., 2013 |
| *P.* sp. MM5482-R1 | KF791796 | *Amphithalea ericifolia* | South Africa | Lemaire et al., 2016 |
| *P.* sp. MM5482-R2 | KF791797 | *Amphithalea ericifolia* | South Africa | Lemaire et al., 2016 |
| *P.* sp. WSM4174 | HE994065 | *Lebeckia ambigua* | South Africa | Howieson et al., 2013 |
| *P.* sp. WSM4180 | HE994070 | *Lebeckia ambigua* | South Africa | Howieson et al., 2013 |
| *P.* sp. WSM4177 | HE994068 | *Lebeckia ambigua* | South Africa | Howieson et al., 2013 |
| *P.* sp. WSM4176 | HE994067 | *Lebeckia ambigua* | South Africa | Howieson et al., 2013 |
| *P.* sp. MM5384 | KF791844 | *Podalyria sericea* | South Africa | Lemaire et al., 2016 |
| *P.* sp. WSM4175 | HE994066 | *Lebeckia ambigua* | South Africa | Howieson et al., 2013 |
| *P.* sp. UCT70 | HF544426 | *Cyclopia maculata* | South Africa | Beukes et al., 2013 |
| *P.* sp. RAU6.4a | HF544367 | *Hypocalyptus oxalidifolius* | South Africa | Beukes et al., 2013 |
| *P.* sp. HC1.1bd | HF544411 | *Hypocalyptus sophoroides* | South Africa | Beukes et al., 2013 |
| *P.* sp. WC7.3b | HF544392 | *Podalyria calyptrata* | South Africa | Beukes et al., 2013 |
| *P.* sp. WC7.3d | HF544394 | *Podalyria calyptrata* | South Africa | Beukes et al., 2013 |
| *P.* sp. WC7.3g | HF544396 | *Podalyria calyptrata* | South Africa | Beukes et al., 2013 |
| P. sp. 7E | LN890818 | *Vachellia karroo* | South Africa | This study |
| *P.* sp. 22.2 | LN890819 | *Vachellia karroo* | South Africa | This study |
| *P.* sp. 9.1 | LN890820 | *Vachellia karroo* | South Africa | This study |
| *P.* sp. 43 | LN890821 | *Vachellia karroo* | South Africa | This study |
| *P.* sp. 16.2 | LN890822 | *Vachellia karroo* | South Africa | This study |
| *P.* sp. 44.1 | LN890823 | *Vachellia karroo* | South Africa | This study |
| *P.* sp. 21 | LN890824 | *Vachellia karroo* | South Africa | This study |
| *P.* sp. 3.1 | LN890825 | *Vachellia karroo* | South Africa | This study |
| *P.* sp. 11 | LN890826 | *Vachellia karroo* | South Africa | This study |
| *P.* sp. 1.2 | LN890827 | *Vachellia karroo* | South Africa | This study |
| *P.* sp. 1.1 | LN890828 | *Vachellia karroo* | South Africa | This study |
| *P.* sp. 11.2 | LN890829 | *Vachellia karroo* | South Africa | This study |
| *P.* sp. 1 | LN890830 | *Vachellia karroo* | South Africa | This study |
| *P.* sp. 29 | LN890831 | *Vachellia karroo* | South Africa | This study |
| *P.* sp. 40 | LN890832 | *Vachellia karroo* | South Africa | This study |
| *P.* sp. 11.1 | LN890833 | *Vachellia karroo* | South Africa | This study |
| *P.* sp. 32 | LN890834 | *Vachellia karroo* | South Africa | This study |
| *P.* sp. 35.1 | LN890835 | *Vachellia karroo* | South Africa | This study |
| *P.* sp. 31.1 | LN890836 | *Vachellia karroo* | South Africa | This study |
| *P*. sp. 9C | LN890837 | *Vachellia karroo* | South Africa | This study |
| *P.* sp. 9A | LN890838 | *Vachellia karroo* | South Africa | This study |
| *P.* sp. MM6595-R1 | KF824750 | *Bolusafra bituminosa* | South Africa | Lemaire et al., 2016 |
| *P.* sp. MM6595-R2 | KF824751 | *Bolusafra bituminosa* | South Africa | Lemaire et al., 2016 |
| *P.* sp. MM6662-R1 | KF824752 | *Rhynchosia capensis* | South Africa | Lemaire et al., 2016 |
| *P.* sp. MM6662-R2 | KF824753 | *Rhynchosia capensis* | South Africa | Lemaire et al., 2016 |
| *P.* sp. MM6592 | KF824748 | *Virgilia divaricata* | South Africa | Lemaire et al., 2016 |
| *P.* sp. BL55 | KF824747 | *Podalyria sericea* | South Africa | Lemaire et al., 2016 |
| *P. s*p. MM5366-R3 | KF791864 | *Virgilia oroboides* | South Africa | Lemaire et al., 2016 |
| *P.* sp. MM5366-R2 | KF791863 | *Virgilia oroboides* | South Africa | Lemaire et al., 2016 |
| *P.* sp. MM5812-R1 | KF791859 | *Dipogon lignosus* | South Africa | Lemaire et al., 2016 |
| *P.* sp. MM5812-R2 | KF791860 | *Dipogon lignosus* | South Africa | Lemaire et al., 2016 |
| *P.* sp. OD28-R2 | KF791856 | *Rafnia* sp. | South Africa | Lemaire et al., 2016 |
| *P.* sp. OD28-R3 | KF791857 | *Rafnia* sp. | South Africa | Lemaire et al., 2016 |
| *P.* sp. OD28-R4 | KF791858 | *Rafnia* sp. | South Africa | Lemaire et al., 2016 |
| *P.* sp. MM5486-R1 | KF791852 | *Rafnia angulata* | South Africa | Lemaire et al., 2016 |
| *P.* sp. MM5486-R2 | KF791853 | *Rafnia angulata* | South Africa | Lemaire et al., 2016 |
| *P.* sp. MM5486-R3 | KF791854 | *Rafnia angulata* | South Africa | Lemaire et al., 2016 |
| *P.* sp. MM6503 | KF791850 | *Podalyria calyptrata* | South Africa | Lemaire, unpubl. |
| *P.* sp. MM6463A-R1 | KF791846 | *Podalyria* sp. | South Africa | Lemaire et al., 2016 |
| *P.* sp. MM6463A-R2 | KF791847 | *Podalyria* sp. | South Africa | Lemaire et al., 2016 |
| *P.* sp. MM6463B | KF791848 | *Podalyria* sp. | South Africa | Lemaire et al., 2016 |
| *P.* sp. MM6463C | KF791849 | *Podalyria* sp. | South Africa | Lemaire et al., 2016 |
| *P.* sp. BL39-R1 | KF791842 | *Podalyria myrtillifolia* | South Africa | Lemaire, unpubl. |
| *P.* sp. BL39-R2 | KF791843 | *Podalyria myrtillifolia* | South Africa | Lemaire, unpubl. |
| *P.* sp. MM6490-R1 | KF791838 | *Podalyria calyptrata* | South Africa | Lemaire et al., 2016 |
| *P.* sp. MM6490-R2 | KF791839 | *Podalyria calyptrata* | South Africa | Lemaire et al., 2016 |
| *P.* sp. MM6490-R3 | KF791840 | *Podalyria calyptrata* | South Africa | Lemaire et al., 2016 |
| *P.* sp. MM6490-R4 | KF791841 | *Podalyria calyptrata* | South Africa | Lemaire et al., 2016 |
| *P.* sp. MM5337-R2 | KF791835 | *Podalyria calyptrata* | South Africa | Lemaire et al., 2016 |
| *P.* sp. MM5337-R3 | KF791836 | *Podalyria calyptrata* | South Africa | Lemaire et al., 2016 |
| *P.* sp. MM25-R4 | KF791833 | *Podalyria calyptrata* | South Africa | Lemaire, unpubl. |
| *P.* sp. OD25-R2 | KF791831 | *Podalyria calyptrata* | South Africa | Lemaire et al., 2016 |
| *P.* sp. OD25-R3 | KF791832 | *Podalyria calyptrata* | South Africa | Lemaire et al., 2016 |
| *P.* sp. BL1-R2 | KF791829 | *Indigofera* sp. | South Africa | Lemaire, unpubl. |
| *P.* sp. MM6502C-R1 | KF791824 | *Indigofera filifolia* | South Africa | Lemaire, unpubl. |
| *P.* sp. MM6502C-R2 | KF791825 | *Indigofera filifolia* | South Africa | Lemaire, unpubl. |
| *P.* sp. MM6502C-R3 | KF791826 | *Indigofera filifolia* | South Africa | Lemaire, unpubl. |
| *P.* sp. MM6502B-R2 | KF791823 | *Indigofera filifolia* | South Africa | Lemaire, unpubl. |
| *P. s*p. MM6502B-R1 | KF791822 | *Indigofera filifolia* | South Africa | Lemaire, unpubl. |
| *P.* sp. MM5819 | KF791820 | *Indigofera cytisoides* | South Africa | Lemaire, unpubl. |
| *P.* sp. CS13775 | KF791818 | *Indigofera ionii* | South Africa | Lemaire, unpubl. |
| *P.* sp. MM6511-R1* | KF791815 | *Hypocalyptus oxalidifolius* | South Africa | Lemaire et al., 2016 |
| *P.* sp. MM6511-R2 | KF791816 | *Hypocalyptus oxalidifolius* | South Africa | Lemaire et al., 2016 |
| *P.* sp. MM6511-R3 | KF791817 | *Hypocalyptus oxalidifolius* | South Africa | Lemaire et al., 2016 |
| *P.* sp. MM5329-R1 | KF791811 | *Bolusafra bituminosa* | South Africa | Lemaire et al., 2016 |
| *P.* sp. MM5329-R2 | KF791812 | *Bolusafra bituminosa* | South Africa | Lemaire et al., 2016 |
| *P.* sp. MM5329-R3 | KF791813 | *Bolusafra bituminosa* | South Africa | Lemaire et al., 2016 |
| *P.* sp. BL8 | KF791810 | *Bolusafra bituminosa* | South Africa | Lemaire et al., 2016 |
| *P.* sp. BL4-R1 | KF791807 | *Bolusafra bituminosa* | South Africa | Lemaire et al., 2016 |
| P. sp. BL4-R2 | KF791808 | *Bolusafra bituminosa* | South Africa | Lemaire et al., 2016 |
| *P.* sp. BL3-R1 | KF791805 | *Bolusafra bituminosa* | South Africa | Lemaire et al., 2016 |
| *P.* sp. BL3-R2 | KF791806 | *Bolusafra bituminosa* | South Africa | Lemaire et al., 2016 |
| *P.* sp. MM5496-R1 | KF791801 | *Aspalathus carnosa* | South Africa | Lemaire et al., 2016 |
| *P.* sp. MM5496-R2 | KF791802 | *Aspalathus carnosa* | South Africa | Lemaire et al., 2016 |
| *P.* sp. MM5496-R3 | KF791803 | *Aspalathus carnosa* | South Africa | Lemaire et al., 2016 |
| *P.* sp. MM5477-R1 | KF791798 | *Aspalathus callosa* | South Africa | Lemaire et al., 2016 |
| *P.* sp. MM5477-R2 | KF791799 | *Aspalathus callosa* | South Africa | Lemaire et al., 2016 |
| *P.* sp. MM5477-R3 | KF791800 | *Aspalathus callosa* | South Africa | Lemaire et al., 2016 |
| *P.* sp. WK1.1c | HF544381 | *Hypocalyptus sophoroides* | South Africa | Beukes et al., 2013 |
| *P.* sp. HC1.1a3 | HF544407 | *Hypocalyptus sophoroides* | South Africa | Beukes et al., 2013 |
| *P.* sp. HC1.1ba | HF544408 | *Hypocalyptus sophoroides* | South Africa | Beukes et al., 2013 |
| *P.* sp. HC1.1bb | HF544409 | *Hypocalyptus sophoroides* | South Africa | Beukes et al., 2013 |

**References:**

Achouak, W., Christen, R., Barakat, M., Martel, M-H., Heulin, T. (1999) *Burkholderia caribensis* sp. nov., an exopolysaccharide-producing bacterium isolated from vertisol microaggregates in Martinique. Int. J. Syst. Bact. 49: 787-794

Aizawa, T., Bao Ve, N., Vijarnsorn, P., Makajima, M., Sunairi, M. (2010a) *Burkholderia acidipaludis* sp. nov., aluminium-tolerant bacteria isolated from Chinese water chestnut (*Eleocharis dulcis*) growing in highly acidic swamps in South-East Asia. Int. J. Syst. Evol. Microbiol. 60: 2036-2041

Aizawa, T., Ve, N.B., Nakajima, M., Sunairi, M. (2010b) *Burkholderia heleia* sp. nov., a nitrogen-fixing bacterium isolated from an aquatic plant, *Eleocharis dulcis*, that grows in highly acidic swamps in actual acid sulfate soil areas of Vietnam. Int. J. Syst. Evol. Microbiol. 60: 1152-1157

Aizawa, T., Vijarnsorn, P., Nakajima, M., Sunairi, M. (2011) *Burkholderia bannensis* sp. nov., an acid-neutralizing bacterium isolated from torpedo grass (*Panicum repens*) growing in highly acidic swamps. Int. J. Syst. Evol. Microbiol. 61: 1645-1650

Baek, I., Seo, B., Lee, I., Yi, H., Chun, J. (2015) *Burkholderia monticola* sp. nov., isolated from mountain soil. Int. J. Syst. Evol. Microbiol. 65: 504-509

Beukes, C.W., Venter, S.N., Law, I.J., Phalane, F.L., Steenkamp, E.T. (2013) South African papilionoid legumes are nodulated by diverse *Burkholderia* with unique nodulation and nitrogen-fixation loci. PLoS ONE 8(7): e68406. doi: 10.1371/journal.pone.0068406

Bournaud, C., Moulin, L., Cnockaert, M., de Faria, S., Prin, Y., Severac, D., Vandamme, P. (2017) *Paraburkholderia piptadeniae* sp. nov. and *Paraburkholderia ribeironis* sp. nov., two root-nodulating symbiotic species of *Piptadenia gonoacantha* in Brazil. Int. J. Syst. Evol. Microbiol. 67: 432-440

Brämer, C.O., Vandamme, P., da Silva, L.F., Gomez, J.G.C., Steinbüchel, A. (2001) *Burkholderia sacchari* sp. nov., a polyhydroxyalkanoate-accumulating bacterium isolated from soil of a sugar-cane plantation in Brazil. Int. J. Syst. Evol. Microbiol. 51:1709-1713

Caballero-Mellado, J., Martínez-Aguilar, L., Paredes-Valdez, G., Estrada-de los Santos, P. (2004) *Burkholderia unamae* sp. nov., an N_2_-fixing rhizospheric and endophytic species. Int. J. Syst. Evol. Microbiol. 54: 1165-1172

Cardova-Kreylos, A.L., Fernandez, L.E., Koivunen, M., Yang, A., Flor-Weiler, l., Marrone, P.G. (2013) Isolation and characterization of *Burkholderia rinojensis* sp. nov., a non-*Burkholderia* *cepacia* complex soil bacterium with insecticidal and miticidal activities. Appl. Environ. Microbiol. 79: 7669-7678

Chen, W-M., de Faria, S.M., Chou, J-H., James, E.K., Elliott, G.N., Sprent, J.I., Bontemps, C., Young, J.P.W., Vandamme, P. (2008) *Burkholderia sabiae* sp. nov., isolated from root nodules of *Mimosa caesalpiniifolia*. Int. J. Syst. Evol. Microbiol. 58: 2174-2179

Chen, W-M., de Faria, S.M., James, E.K., Elliott, G.N., Lin, K-Y., Chou, J-H., Sheu, S-Y., Cnockaert, M., Sprent, J.I., Vandamme, P. (2007) *Burkholderia nodosa* sp. nov., isolated from root nodules of the woody Brazilian legumes *Mimosa bimucronata* and *Mimosa* *scabrella*. Int. J. Syst. Evol. Microbiol. 57: 1055-1059

Chen, W-M., James, E.K., Coenye, T., Chou, J-H., Barrios, E., de Faria, S.M., Elliott, G.N., Sheu, S-Y., Sprent, J.I., Vandamme, P. (2006) *Burkholderia mimosarum* sp. nov., isolated from root nodules of *Mimosa* spp. from Taiwan and South America. Int. J. Syst. Evol. Microbiol. 56: 1847-1851

Coenye, T., Henry, D., Speert, D.P., Vandamme, P. (2004) *Burkholderia phenoliruptrix* sp. nov., to accommodate the 2,4,5-trichlorophenoxyacetic acid and halophenol-degrading strain AC1100. Syst. Appl. Microbiol. 27: 623-627

Coenye, T., Laevens, S., Willems, A., Ohlén, M., Hannant, W., Govan, J.R.W., Gillis M., Falsen, E., Vandamme, P. (2001) *Burkholderia fungorum* sp. nov. and *Burkholderia caledonica* sp. nov., two new species isolated from the environment, animals and human clinical samples. Int. J. Syst. Evol. Microbiol. 51: 1099-1107

De Meyer, S.E., Cnockaert, M., Adrley, J.K., Maker, G., Yates, R., Howieson, J.G., Vandamme, P. (2013a) *Burkholderia sprentiae* sp. nov., isolated from *Lebeckia* *ambigua* root nodules. Int. J. Syst. Evol. Microbiol. 63: 3950-3957

De Meyer, S.E., Cnockaert, M., Ardley, J.K., Trengove, R.D., Garau, G., Howieson, J.G., Vandamme, P. (2013b) *Burkholderia rhynchosiae* sp. nov., isolated from *Rhynchosia ferulifolia* root nodules. Int. J. Syst. Evol. Microbiol. 63: 3944-3949

De Meyer, S.E., Cnockaert, M., Ardley, J.K., Van Wyk, B-E., Vandamme, P.A., Howieson, J.G. (2014) *Burkholderia dilworthii* sp. nov., isolated from *Lebeckia ambigua* root nodules. Int. J. Syst. Evol. Microbiol. 64:1090-1095

El-Agamy Farh, M., Kim, Y-J., Van An, H., Sukweenadhi, J., Singh, P., Huq, M.A., Yang, D-C. (2015) *Burkholderia ginsengiterrae* sp. nov. and *Burkholderia panaciterrae* sp. nov., antagonistic bacteria against root rot pathogen *Cylindrocarpon destructans*, isolated from ginseng soil. Arch. Microbiol. 197: 439-447

Gao, Z., Yuan, Y., Xu, L., Liu, R., Chen, M., Zhang, C. (2016a) *Paraburkholderia caffeinilytica* sp. nov., isolated from the soil of a tea plantation. Int. J. Syst. Evol. Microbiol. Doi: 10.1099/ijsem.0.001333

Gao, Z-Q., Zhao, D-Y., Xu, L., Zhao, R-T., Chen, M., Zhang, C-Z. (2016b) *Paraburkholderia caffeinitolerans* sp. nov., a caffeine degrading species isolated from a tea plantation soil sample. Antonie van Leeuwenhoek 109: 1475-1482

Goris, J., Dejonghe, W., Falsen, E., De Clerck, E., Geeraerts, B., Willems, A., Top, E.M., Vandamme, P., De Vos, P. (2002) Diversity of transconjugants that acquired plasmid pJP4 or pEMT1 after inoculation of a donor strain in the A- and B-horizon of an agricultural soil and description of *Burkholderia hospita* sp. nov. and *Burkholderia terricola* sp. nov. Syst. Appl. Microbiol. 25: 340-352

Goris, J., De Vos, P., Caballero-Mellado, J., Park, J., Falsen, E., Quensen, J.F. III, Tiedje, J.M., Vandamme, P. (2004) Classification of the biphenyl- and polychlorinated biphenyl-degrading strain LB400^T^ and relatives as *Burkholderia xenovorans* sp. nov. Int. J. Syst. Evol. Microbiol. 54: 1677-1681

Guentas, L., Gensous, S., Cavaloc, Y., Ducousso, M., Amir, H., De Georges de Ledenon, B., Moulin, L., Jourand, P. (2016) *Burkholderia novacaledonica* sp. nov. and *B. ultramafica* sp. nov. isolated from roots of *Costularia* spp. pioneer plants of ultramafic soils in New Caledonia. Syst. Appl. Microbiol. 39: 151-159

Gu, J-Y., Zang, S-G., Sheng, X-F., He, L-Y., Huang, Z., Wang, Q. (2015) *Burkholderia susongensis* sp. nov., a mineral-weathering bacterium isolated from weathered rock surface. Int. J. Syst. Evol. Microbiol. 65: 1031-1037

Howieson, J.G., De Meyer, S.E., Vivas-Marfisi, A., Ratnayake, S., Ardley, J.K., Yates, R.J. (2013) Novel *Burkholderia* bacteria isolated from *Lebeckia ambigua* – A perennial suffrutescent legume of the fynbos. Soil Biology & Biochemistry 60: 55-64

Kim, H-B., Park, M-J., Yang, H-C., An, D-S., Jin H-Z., Yang, D-C. (2006) *Burkholderia ginsengisoli* sp. nov., a β-glucosidase-producing bacterium isolated from soil of a ginseng field. Int. J. Syst. Evol. Microbiol. 56:2529-2533

Lee, J-C., Whang, K-S. (2015) *Burkholderia humisilvae* sp. nov., *Burkholderia solisilvae* sp. nov. and *Burkholderia rhizosphaerae* sp. nov., isolated from forest soil and rhizosphere soil. Int. J. Syst. Evol. Microbiol. 65; 2986-2992

Lemaire, B., Chimphango, S.B.M., Stirton, C., Rafudeen, S., Honnay, O., Smets, E., Chen, W-M., Sprent, J.I., James, E.K., Muasya, A.M. (2016) Biogeographical patterns of legume-nodulating *Burkholderia* spp.: from African fynbos to continental scales. Appl. Environ. Microbiol. 82: 5099-5115

Lemaire, B., Dlodlo, O., Chimphango, S., Stirton, C., Schrire, B., Boatwright, J.S., Honnay, O., Smets, E., Sprent, J., James, E.K., Muasya, A.M. (2015a) Symbiotic diversity, specificity and distribution of rhizobia in native legumes of the Core Cape Subregion (South Africa). FEMS Microbiology Ecology 91. doi: 10.1093/femsec/fiu024

Lim, J.H., Baek, S-H., Lee, S-T. (2008) *Burkholderia sediminicola* sp. nov., isolated from freshwater sediment. Int. J. Syst. Evol. Microbiol. 58: 565-569

Lv, Y-Y., Chen, M-H., Xia, F., Wang, J., Qiu, L-H. (2016) *Paraburkholderia pallidirosea* sp. nov., isolated from a monsoon evergreen broad-leaved forest soil. Int. J. Syst. Evol. Microbiol. 66: 4537-4542

Martínez-Aguilar, L., Salazar-Salazar, C., Díaz Méndez, R., Caballero-Mellado, J., Hirsch, A.M., Vásquez-Murrieta, M.S., Estrada-de los Santos, P. (2013) *Burkholderia caballeronis* sp. nov., a nitrogen fixing species isolated from tomato (*Lycopersicon esculentum*) with the ability to effectively nodulate *Phaseolus vulgaris*. Antonie van Leeuwenhoek 104: 1063-1071

Mavengere, N.R., Ellis, A.G., Le Roux, J.J. (2014) *Burkholderia aspalathi* sp. nov., isolated from root nodules of the South African legume *Aspalathus abietina* Thunb. Int. J. Syst. Evol. Microbiol. 64: 1906-1912

Otsuka, Y., Muramatsu, Y., Nakagawa, Y., Matsuda, M., Nakamura, M., Murata, H. (2011) *Burkholderia oxyphila* sp. nov., a bacterium isolated from acidic forest soil that catabolizes (+)-catechin and its putative aromatic derivatives. Int. J. Syst. Evol. Microbiol. 61: 249-254

Partida-Martinez, L.P., Groth, I., Schmitt, I., Richter, W., Roth, M., Hertweck, C. (2007) *Burkholderia rhizoxinica* sp. nov, and *Burkholderia endofungorum* sp. nov., bacterial endosymbionts of the plant-pathogenic fungus *Rhizopus microsporus*. Int. J. Syst. Evol. Microbiol. 57: 2583-2590

Perin, L., Martínez-Aguilar, L., Paredes-Valdez, G., Baldani, J.I., Estrada-de los Santos, P., Reis, V.M., Caballero-Mellado, J. (2006) *Burkholderia silvatlantica* sp. nov., a diazotrophic bacterium associated with sugar cane and maize. Int. J. Syst. Evol. Microbiol. 56: 1931-1937

Reis, V.M., Estrada-de los Santos, P., Tenorio-Salgado, S., Vogel, J., Stoffels, M., Guyon, S., Mavingui, P., Baldani, V.L.D., Schmid, M., Baldani, J.I., Balandreau, J., Hartmann. A., Caballero-Mellado, J. (2004) *Burkholderia tropica* sp. nov., a novel nitrogen-fixing, plant-associated bacterium. Int. J. Syst. Evol. Microbiol. 54: 2155-2162

Sessitsch, A., Coenye, T., Sturz, A.V., Vandamme, P., Barka, E.A., Salles, J.F., Van Elsas, J.D., Faure, D., Reiter, B., Glick, B.R., Wang-Pruski, G., Nowak, J. (2005) *Burkholderia phytofirmans* sp. nov., a novel plant-associated bacterium with plant-beneficial properties. Int. J. Syst. Evol. Microbiol. 55: 1187-1192

Sheu, S-Y., Chen, M-H., Liu, W.Y.Y., Andrews, M., James, E.K., Ardley, J.K., De Meyer, S.E., James, T.K., Howieson, J.G., Coutinho, B.G., Chen, W-M. (2015a) *Burkholderia dipogonis* sp. nov., isolated from root nodules of *Dipogon lignosus* in New Zeland and Western Australia. Int. J. Syst. Evol. Microbiol. 65: 4716-4723 doi: 10.1099/ijsem.0.000639

Sheu, S-Y., Chou, J-H., Bontemps, C., Elliott, G.N., Gross, E., dos Reis Junior, F.B., Melkonian, R., Moulin, L., James, E.K., Sprent, J.I., Young, J.P.W., Chen, W-M. (2013) *Burkholderia diazotrophica* sp. nov., isolated from root nocules of *Mimosa* spp. Int. J. Syst. Evol. Microbiol. 63: 435-441

Sheu, S-Y., Chou, J-H., Bontemps, C., Elliott, G.N., Gross, E., James, E.K., Sprent, J.I., Young, J.P.W., Chen, W-M. (2012) *Burkholderia symbiotica* sp. nov., isolated from root nodules of *Mimosa* spp. native to north-east Brazil. Int. J. Syst. Evol. Microbiol. 62: 2272-2278

Steenkamp, E.T., van Zyl, E., Beukes, C.W., Avontuur, J.R., Chan, W.Y., Palmer, M., Mthombeni, L.S., Phalane, F.L., Sereme, T.K., Venter, S.N. (2015) *Burkholderia kirstenboschensis* sp. nov. nodulates papilionoid legumes indigenous to South Africa. Syst. Appl. Microbiol. 38: 545-554

Valverde, A., Delvasto, P., Peix, A., Velázquez, E., Santa-Regina, I., Ballester, A., Rodríguez-Barrueco, C., García-Balboa, C., Igual, J.M. (2006) *Burkholderia ferrariae* sp. nov., isolated from an iron ore in Brazil. Int. J. Syst. Evol. Microbiol. 56: 2421-2425

Vandamme, P., Goris, J., Chen, W-M., de Vos, P., Willems, A. (2002) *Burkholderia tuberum* sp. nov. and *Burkholderia phymatum* sp. nov., nodulate the roots of tropical legumes. Syst. Appl. Microbiol. 25: 507-512

Vandamme, P., Opelt, K., Knöchel, N., Berg, C., Schönmann, S., De Brandt, E., Eberl, L., Falsen, E., Berg, G. (2007) *Burkholderia bryophila* sp. nov. and *Burkholderia megapolitana* sp. nov., moss-associated species with antifungal and plant-growth-promoting properties. Int. J. Syst. Evol. Microbiol. 57: 2228-2235

Vanlaere, E., van der Meer, J.R., Falsen, E., Salles, J.F., de Brandt, E., Vandamme, P. (2008) *Burkholderia sartisoli* sp. nov., isolated from a polycyclic aromatic hydrocarbon-contaminated soil. Int. J. Syst. Evol. Microbiol. 58: 420-423

Viallard, V., Poirier, I. Cournoyer, B., Haurat, J., Wiebkin, S., Ophel-Keller, K., Balandreau, J. (1998) *Burkholderia graminis* sp. nov., a rhizospheric *Burkholderia* species, and reassessment of [*Pseudomonas*] *phenazinium*, [*Pseudomonas*] *pyrrocinia* and [*Pseudomonas*] *glathei* as *Burkholderia*. Int. J. Syst. Bacteriol. 48: 549-563

Weber, C.F., King, G.M. (2017) Volcanic soils as sources of novel CO-oxidizing *Paraburkholderia* and *Burkholderia*: *Paraburkholderia hiiakae* sp. nov., *Paraburkholderia metrosideri* sp. nov., *Paraburkholderia paradise* sp. nov., *Paraburkholderia peleae* sp. nov., and *Burkholderia* *alpina* sp. nov. a member of the *Burkholderia* *cepacia* complex. Frontiers in Microbiology. doi: 10.3389/fmicb.2017.00207

Yabuuchi, E., Kosako, Y., Oyaizu, H., Yano, I., Hotta, H., Hashimoto, Y., Ezaki, T., Arakawa, M. (1992) Proposal of *Burkholderia* gen. nov. and transfer of seven species of the genus *Pseudomonas* homology group II to the new genus, with the type species *Burkholderia cepacia* (Palleroni and Holmes 1981) comb. nov. Microbiol. Immunol. 36: 1251-1275

Yang, H-C., Im, W-T., Kim, K.K., An, D-S., Lee, S-T. (2006) *Burkholderia terrae* sp. nov., isolated from a forest soil. Int. J. Syst. Evol. Microbiol. 56: 453-457

Yoo, S-H., Kim, B-Y., Weon, H-Y., Kwon, S-W., Go, S-J., Stackebrandt, E. (2007) *Burkholderia soli* sp. nov., isolated from soil cultivated with Korean ginseng. Int. J. Syst. Evol. Microbiol. 57: 122-125

Zhang, H., Hanada, S., Shigematsu, T., Shibuya, K., Kamagata, Y., Kanagawa, T., Kurane, R. (2000) *Burkholderia kururiensis* sp. nov., a trichloroethylene (TCE)-degrading bacterium isolated from an aquifer polluted with TCE. Int. J. Syst. Evol. Microbiol. 50: 743-749
