# Supplementary material for: Both Alpha- and Beta-Rhizobia Occupy the Root Nodules of Vachellia karroo in South Africa
Source: Front Microbiol. 2019 Jun 4;10:1195. doi: 10.3389/fmicb.2019.01195 (PMC6558075; doi:10.3389/fmicb.2019.01195)
Supplement: Supplementary file 4 [file Table_4.DOCX]

**Supplementary Table S4** Isolate names, accession numbers, host/niche, country of origin and references for isolates of the genus *Rhizobium* included in this study

| **Isolate** | ***recA*** | **Host/Niche** | **Country** | **Reference** |
| --- | --- | --- | --- | --- |
| *R. acidisoli* FH13^T^ | KJ921098 | *Phaseolus vulgaris* | Mexico | Román-Ponce et al., 2016 |
| *R. aegyptiacum* 1010^T^ | KU664569 | *Trifolium alexandrinum* | Egypt | Shamseldin et al., 2016 |
| *R. aethiopicum* HBR26^T^ | GA0061105_101306 | *Phaseolus vulgaris* | Ethiopia | Aserse et al., 2017 |
| *R. alamii* GBV016^T^ | GU552971 | *Arabidopsis thaliana* rhizosphere | France | Berge et al., 2009 |
| *R. altiplani* BR10423^T^ | KX022644 | *Mimosa pudica* | Brazil | Baraúna et al., 2016 |
| *R. alvei* TNR-22^T^/LMG26895^T^ | KX938338 | Freshwater river | Taiwan | Sheu et al., 2015b |
| *R. anhuiense* CCBAU23252^T^ | KF111980 | *Vicia faba* | China | Zhang et al., 2015 |
| *R. arenae* MIM27^T^ | BLX89_RS02000 | Desert sand | China | Zhang et al., 2017 |
| *R. azibense* 23C2^T^ | JN624700 | *Phaseolus vulgaris* | Tunisia | Mnasri et al., 2014 |
| *R. azooxidifex* Po20/26^T^ | LN868516 | Soil core of a long-term experiment | Germany | Behrendt et al., 2016 |
| *R. bangladeshense* BLR175^T^ | JN649057 | *Lens culinaris* | Bangladesh | Rashid et al., 2015 |
| *R. binae* BLR195^T^ | JN649058 | *Lens culinaris* | Bangladesh | Rashid et al., 2015 |
| *R. calliandrae* CCGE524^T^ | JX855189 | *Calliandra grandiflora* | Mexico | Rincón-Rosales et al., 2013 |
| *R. capsici* CC-SKC2^T^ | KJ863428 | *Capsicum annuum* var. grossum | Taiwan | Lin et al., 2015 |
| *R. cauense* CCBAU101002^T^ | JQ308335 | *Kummerowia stipulacea* | China | Liu et al., 2012 |
| *R. cellulosilyticum* ALA10B2^T^/LMG23642^T^ | AM286427 | *Populus alba* sawdust | Spain | García-Fraile et al., 2007 |
| *R. ecuadorense* CNPSo671^T^ | AF337_RS09880 | *Phaseolus vulgaris* | Ecuador | Ribeiro et al., 2015 |
| *R. endophyticum* CCGE2052^T^ | HM142767 | *Phaseolus vulgaris* | Mexico | López-López et al., 2010 |
| *R. endolithicum* JC140^T^ | NA | Endolithic beach sand sample | India | Parag et al., 2013 |
| *R. esperanzae* CNPSo668^T^ | B5E41_RS24975 | *Phaseolus vulgaris* | Mexico | Cordeiro et al., 2017 |
| *R. etli* CFN42^T^ | RHE_RS11870 | *Phaseolus vulgaris* | Mexico | Segovia et al., 1993 |
| *R. fabae* CCBAU33202^T^ | EF579941 | *Vicia faba* | China | Tian et al., 2008 |
| *R. favelukesii* LPU83^T^ | LPU83_RS11345 | *Medicago sativa* | Argentina | Tejerizo et al., 2016 |
| *R. flavum* YW14^T^ | B0X76_RS03400 | Soil | China | Gu et al., 2014 |
| *R. freirei* PRF81^T^ | RHSP_RS03330 | *Phaseolus vulgaris* | Brazil | Dall’Agnol et al., 2013 |
| *R. gallicum* R602sp^T^ | B028_RS0104370 | *Phaseolus vulgaris* | France | Amarger et al., 1997 |
| *R. gei* ZFJT-2^T^ | KF751875 | *Geum aleppicum* stem | China | Shi et al., 2016 |
| *R. grahamii* CCGE502^T^ | RGCCGE502_10616 | *Dalea leporina* | Mexico | López-López et al., 2012 |
| *R. hainanense* I66^T^/CCBAU57015^T^ | GA0061100_10185 | *Desmodium sinautum* | China | Chen et al., 1997 |
| *R. halophytocola* YC6881^T^ | HQ174465 | *Rosa rugosa* | Korea | Bibi et al., 2012 |
| *R. hedysari* 5-1-2^T^ | KX095241 | *Hedysarum multijugum* | China | Xu et al., 2017 |
| *R. helianthi* Xi19^T^ | JX094430 | *Helianthus annuus* rhizosphere | Mongolia | Wei et al., 2015 |
| *R. hidalgonense* FH14^T^ | KJ921099 | *Phaseolus vulgaris* | Mexico | Yan et al., 2017 |
| *R. indigoferae* CCBAU71042^T^ | EF027965 | *Indigofera amblyantha* | China | Wei et al., 2002 |
| *R. ipomoeae* shin9-1^T^ | LN890297 | Freshwater sample | Taiwan | Sheu et al., 2016 |
| *R. jaguaris* CCGE525^T^ | JX855192 | *Calliandra grandiflora* | Mexico | Rincón-Rosales et al., 2013 |
| *R. laguerreae* FB206^T^ | BS630_RS16725 | *Vicia faba* | Tunisia | Saïdi et al., 2014 |
| *R. larrymoorei* AF3.10^T^ | FN432355 | *Ficus benjamina* | USA | Bouzar and Jones, 2001 |
| *R. leguminosarum* USDA2370^T^ | AJ294376 | *Pisum sativum* | Unknown |  |
| *R. lemnae* L6-16^T^ | AB746183 | *Lemna aequinoctialis* | Thailand | Kittiwongwattana and Thawai, 2014 |
| *R. lentis* BLR27^T^ | JN649031 | *Lens culinaris* | Bangladesh | Rashid et al., 2015 |
| *R. leucaenae* LMG9517^T^/USDA9039^T^ | K290_RS0116985 | *Phaseolus vulgaris* | Brazil | Ribeiro et al., 2012 |
| *R. loessense* CCBAU7190B^T^/LMG21975^T^ | HQ735076 | *Astragalus complanatus* | China | Wei et al., 2003 |
| *R. lusitanum* P1-7^T^ | GA0061101_105463 | *Phaseolus vulgaris* | Portugal | Valverde et al., 2006 |
| *R. marinum* MGL06^T^ | EO99_RS0108850 | Surface seawater sample | South China Sea | Liu et al., 2015 |
| *R. mayense* CCGE526^T^ | JX855195 | *Calliandra grandiflora* | Mexico | Rincón-Rosales et al., 2013 |
| *R. mesoamericanum* CCGE501^T^ | JF424620 | *Phaseolus vulgaris* | Mexico | López- López et al., 2012 |
| *R. mesosinicum* CCBAU25010^T^ | EU120732 | *Albizia julibrissin* | China | Lin et al., 2009 |
| *R. metallidurans* ChimEc512^T^ | KF863912 | *Anthyllis vulneraria* | France | Grison et al., 2015 |
| *R. miluonense* CCBAU41251^T^ | HN047131 | *Lespedeza chinensis* | China | Gu et al., 2008 |
| *R. mongolense* USDA1844^T^ | A3C3_RS0113875 | *Medicago ruthenica* | Mongolia | Van Berkum et al., 1998 |
| *R. multihospitium* CCBAU83401^T^/HAMBI2975^T^ | GA0061103_6319 | *Halimodendron halodendron* | China | Han et al., 2008 |
| *R. naphthalenivorans* TSY03b^T^ | AB684355 | Polychlorinated-dioxin-transforming microcosm | Unknown | Kaiya et al., 2012 |
| *R.* *oryzae* Alt505^T^ | FJ712274 | *Oryza alta* | China | Peng et al., 2008 |
| *R. oryzicola* ZYY136^T^ | JX443617 | Rice roots | China | Zhang et al., 2015 |
| *R. oryziradicis* N19^T^ | BJF95_RS17670 | *Oryza sativa* roots | China | Zhao et al., 2017a |
| *R. pakistanense* BN-19^T^ | AB855792 | *Arachis hypogaea* | Pakistan | Khalid et al., 2015 |
| *R. paranaense* PRF35^T^ | EU488826 | *Phaseolus vulgaris* | Brazil | Dall’Agnol et al., 2014 |
| *R. petrolearium* SL-1^T^ | EU556970 | Petroleum-contaminated sludge | China | Zhang et al., 2012 |
| *R. phaseoli* ATCC14482^T^ | EF113136 | *Phaseolus vulgaris* | Unknown | Ramírez-Bahena et al., 2008 |
| *R. pisi* DSM30132^T^ | EF113134 | *Pisum sativum* | Unknown | Ramírez-Bahena et al., 2008 |
| *R. populi* K-38^T^ | KF951420 | *Populus euphratica* | China | Rozahon et al., 2014 |
| *R. puerariae* PC004^T^ | LC014932 | *Pueraria candollei* | Thailand | Boonsnongcheep et al., 2016 |
| *R. rhizogenes* IFO/NBRC13257^T^ | RRH01S_01_06590 | Apple | Unknown | Young et al., 2001 |
| *R. rhizoryzae* J3-AN59^T^ | KF384476 | Rice roots | China | Zhang et al., 2014a |
| *R. rhizosphaerae* MH17^T^ | BJF92_RS11330 | Rice rhizosphere | China | Zhao et al., 2017b |
| *R. rosettiformans* W3^T^ | GU562963 | HCH dump site | India | Kaur et al., 2011 |
| *R. rubi* NBRC13261^T^ | RRU01S_13-00900 | *Rubus ursinus* var. loganobaccus | USA | Young et al., 2001 |
| *R. selenitireducens* B1^T^/ATCCBAA-1503^T^ | L867_RS0102245 | Bioreactor | USA | Hunter et al., 2007 |
| *R. smilacinae* PTYR-5^T^ | KF738708 | *Smilacina japonica* leaf | China | Zhang et al., 2014b |
| *R. soli* DS-42^T^ | KF206885 | Soil | South Korea | Yoon et al., 2010 |
| *R. sophorae* CCBAU03386^T^ | BTE55_RS02810 | *Sophora flavescens* | China | Jiao et al., 2015 |
| *R. sophoriradicis* CCBAU03470^T^ | KJ831248 | *Sophora flavescens* | China | Jiao et al., 2015 |
| *R. straminoryzae* CC-LY845^T^ | KJ863426 | Rice straw | Taiwan | Lin et al., 2014 |
| *R. subbaraonis* JC85^T^ | HE572579 | Beach sand | India | Ramana et al., 2013 |
| *R. sullae* IS123^T^ | FJ816279 | *Hedysarum coronarium* | Spain | Squartini et al., 2002 |
| *R. tarimense* PL-41^T^ | JF508523 | *Populus euphratica* forest soil | China | Turdahon et al., 2013 |
| *R. tibeticum* CCBAU85039^T^ | RTCCBAU85039_3393 | *Trigonella archiducis-nicolai* | Tibet | Hou et al., 2009 |
| *R. tropici* CIAT899^T^ | RTCIAT899_CH09935 | *Phaseolus vulgaris* | Colombia | Martínez-Romero et al., 1991 |
| *R. tubonense* CCBAU85046^T^ | EU288696 | *Oxytropis glabra* | Tibet | Zhang et al., 2011 |
| *R. vallis* CCBAU65647^T^ | GU211770 | *Phaseolus vulgaris* | China | Wang et al., 2011 |
| *R. vignae* CCBAU05176^T^ | GQ59_RS13520 | *Astragalus dahuricus* | China | Ren et al., 2011 |
| *R. viscosum* LMG16473^T^ | KX938339 | Soil | Guatemala | Flores-Félix et al., 2017 |
| *R. wenxiniae* 166^T^ | KY630509 | Maize root endophyte | China | Gao et al., 2017 |
| *R. yanglingense* SH22623^T^ | AY907359 | *Gueldenstaedtia multiflora* | China | Tan et al., 2001 |
| *R. yantingense* H66^T^ | KM029983 | Surface of weathered rock | China | Chen et al., 2015 |
| *Rhizobium zeae* CRZM18R^T^ | KX938341 | Maize roots | Spain | Celador-Lera et al., 2017 |
| *Rhizobium* sp. 63-7 | BGN83_15445 | Thiocyanate ramp bioreactor | South Africa | Unpubl. |
| *Rhizobium* sp. 60-20 | BGP09_22215 | Ammonium sulfate bioreactor | South Africa | Unpubl. |
| *R.* sp. AC100a | JF970573 | *Senegalia senegal* | Ethiopia | Degefu et al., 2013 |
| *R.* sp. AC100b | JF970574 | *Senegalia senegal* | Ethiopia | Degefu et al., 2013 |
| *R.* sp. AC93d | JF970571 | *Gliricidia sepium* | Ethiopia | Degefu et al., 2013 |
| *R.* sp. AC56b | JF970539 | *Vicia unguiculata* | Ethiopia | Degefu et al., 2013 |
| *R.* sp. AC82b | JF970545 | *Vachellia gummifera* | Ethiopia | Degefu et al., 2013 |
| *R.* sp. AC85b | JF970546 | *Vicia faba* | Ethiopia | Degefu et al., 2013 |
| *R.* sp. AC85d | JF970547 | *Vicia faba* | Ethiopia | Degefu et al., 2013 |
| *R.* sp. AC85e | JF970548 | *Vicia faba* | Ethiopia | Degefu et al., 2013 |
| *R.* sp. AC86a2 | JF970549 | *Cajanus cajan* | Ethiopia | Degefu et al., 2013 |
| *R.* sp. AC86c1 | JF970550 | *Cajanus cajan* | Ethiopia | Degefu et al., 2013 |
| *R.* sp. AC86c2 | JF970551 | *Cajanus cajan* | Ethiopia | Degefu et al., 2013 |
| *R.* sp. AC89a | JF970555 | *Leucaena leucocephala* | Ethiopia | Degefu et al., 2013 |
| *R.* sp. AC89d | JF970558 | *Leucaena leucocephala* | Ethiopia | Degefu et al., 2013 |
| *R.* sp. AC90b | JF970561 | *Gliricidia sepium* | Ethiopia | Degefu et al., 2013 |
| *R.* sp. AC91a | JF970565 | *Calliandra calothyrsus* | Ethiopia | Degefu et al., 2013 |
| *R.* sp. AC91c | JF970566 | *Calliandra calothyrsus* | Ethiopia | Degefu et al., 2013 |
| *R.* sp. AC87k1 | JF970552 | *Millettia ferruginea* | Ethiopia | Degefu et al., 2013 |
| *R.* sp. AC87k3 | JF970553 | *Millettia ferruginea* | Ethiopia | Degefu et al., 2013 |
| *R.* sp. AC87k4 | JF970554 | *Millettia ferruginea* | Ethiopia | Degefu et al., 2013 |
| *R.* sp. OD53 | KF802764 | *Aspalathus* sp. | South Africa | Lemaire et al., 2015 |
| *R.* sp. OD49 | KF802763 | *Aspalathus* sp. | South Africa | Lemaire et al., 2015 |
| *R.* sp. OD24 | KF802786 | *Psoralea* sp. | South Africa | Lemaire et al., 2015 |
| *R.* sp. 11A | LN890817 | *Vachellia karroo* | South Africa | This study |
| *R.* sp. 6B | LN890816 | *Vachellia karroo* | South Africa | This study |
| *R.* sp. 20B | LN890815 | *Vachellia karroo* | South Africa | This study |
| *R.* sp. 20C | LN890814 | *Vachellia karroo* | South Africa | This study |
| *R.* sp. 21A | LN890813 | *Vachellia karroo* | South Africa | This study |
| *R.* sp. 21B | LN890812 | *Vachellia karroo* | South Africa | This study |
| *R.* sp. 1B | LN890811 | *Vachellia karroo* | South Africa | This study |
| *R.* sp. AC26e | JF970538 | *Vachellia tortilis* | Ethiopia | Degefu et al., 2013 |
| *R.* sp. AC4d | JF970537 | *Vachellia seyal* | Ethiopia | Degefu et al., 2013 |
| *R.* sp. RITF1463 | JX277160 | *Acacia melanoxylon* rhizosphere | China | Dou, unpubl. |
| *R.* sp. RITF1461 | JX277159 | *Acacia melanoxylon* rhizosphere | China | Dou, unpubl. |
| *R.* sp. RITF1412 | JX277156 | *Acacia melanoxylon* rhizosphere | China | Dou, unpubl. |
| *R.* sp. TW | HM064008 | *Acacia confusa* | China | Lu, unpubl. |
| *R.* sp. 62-1-1 | KM378416 | *Vigna unguiculata* | Namibia | Grönemeyer et al., 2014 |
| *R.* sp. 53-1-1 | KM378370 | *Phaseolus vulgaris* | Angola | Grönemeyer et al., 2014 |
| *R.* sp. 53-2-1 | KM378371 | *Phaseolus vulgaris* | Angola | Grönemeyer et al., 2014 |
| *R.* sp. 52-3-1 | KM378369 | *Phaseolus vulgaris* | Angola | Grönemeyer et al., 2014 |
| *R.* sp. 52-2-2 | KM378368 | *Phaseolus vulgaris* | Angola | Grönemeyer et al., 2014 |
| *R.* sp. 43-3-1 | KM378367 | *Phaseolus vulgaris* | Angola | Grönemeyer et al., 2014 |
| *R.* sp. 43-2-1 | KM378425 | *Phaseolus vulgaris* | Angola | Grönemeyer et al., 2014 |
| *R.* sp. 54-3-1 | KM378432 | *Vigna subterranea* | Namibia | Grönemeyer et al., 2014 |

**References:**

Amarger, N., Macheret, V., Laguerre, G. (1997) *Rhizobium gallicum* sp. nov. and *Rhizobium giardinii* sp. nov., from *Phaseolus vulgaris* nodules. Int. J. Syst. Bacteriol. 47: 996-1006

Aserse, A.A., Woyke, T., Kyrpides, N.C., Whitman, W.B., Lindström, K. (2017) Draft genome sequence of type strain HBR26^T^ and description of *Rhizobium* *aethiopicum* sp. nov. Standards in Genomic Sciences 12:14 doi: 10.1186/s40793-017-0220-z

Baraúna, A.C., Rouws, L.F.M., Simoes-Araujo, J.L., dos Reis Junior, F.B., Iannetta, P.P., Maluk, M., Goi, S.R., Reis, V.M., James, E.K., Zilli, J.E. (2016) *Rhizobium altiplani* sp. nov., isolated from effective nodules on *Mimosa pudica* growing in untypically alkaline soil in central Brazil. Int. J. Syst. Evol. Microbiol. 66: 4118-4124

Behrendt, U., Kämpfer, P., Gleaser, S.P., Augustin, J., Ulrich, A. (2016) Characterization of the N_2_O-producing soil bacterium *Rhizobium azooxidifex* sp. nov. Int. J. Syst. Evol. Microbiol. 66: 2354-2361.Bibi, F., Chung, E.J., Khan, A., Jeon, C.O., Chung, Y.R. (2012) *Rhizobium halophytocola* sp. nov., isolated from the root of a coastal dune plant. Int. J. Syst. Evol. Microbiol. 62: 1997-2003

Berge, O. Lodhi, A., Brandelet, G., Santaella, C., Roncato, M-A., Christen, R., Heulin, T., Achouak, W. (2009) *Rhizobium alamii* sp. nov., an exopolysaccharide-producing species isolated from legume and non-legume rhizospheres. Int. J. Syst. Evol. Microbiol. 59: 367-372

Boonsnongcheep, P., Prathanturarug, S., Takahashi, Y., Matsumoto, A. (2016) *Rhizobium puerariae* sp. nov., an endophytic bacterium from the root nodules of the medicinal plant *Pueraria candollei* var. *candollei*. Int. J. Syst. Evol. Microbiol. 66: 1236-1241

Bouzar, H., Jones, J.B. (2001) *Agrobacterium larrymoorei* sp. nov., a pathogen isolated from aerial tumours of *Ficus benjamina*. Int. J. Syst. Evol. Microbiol. 51: 1023-1026

Celador-Lera, L., Menéndez, E., Peix, A., Igual, J.M., Velázquez, E., Rivas, R. (2017) *Rhizobium zeae* sp. nov., isolated from maize (*Zea mays* L.) roots. Int. J. Syst. Evol. Microbiol. 67: 2306-2311

Chen, W., Sheng, X-F., He, L-Y., Huang, Z. (2015) *Rhizobium yantingense* sp. nov., a mineral-weathering bacterium. Int. J. Syst. Evol. Microbiol. 65: 412-417

Chen, W-X., Tan, Z-Y., Gao, J-L., Li, Y., Wang, E-T. (1997) *Rhizobium hainanense* sp. nov., isolated from tropical legumes. Int. J. Syst. Bacteriol. 47: 870-873

Cordeiro, A.B., Ribeiro, R.A., Ferraz Helene, L.C., Hungria., M. (2017) *Rhizobium esperanzae* sp. nov., a N_2_-fixing root symbiont of *Phaseolus vulgaris* from Mexican soils. Int. J. Syst. Evol. Microbiol. 67: 3937-3945

Dall’Agnol, R.F., Ribeiro, R.A., Delamuta, J.R.M., Ormeño-Orrillo, E., Rogel, M.A., Andrade, D.S., Martínez-Romero, E., Hungria, M. (2014) *Rhizobium paranaense* sp. nov., an effective N_2_-fixing symbiont of common bean (*Phaseolus vulgaris* L.) with broad geographical distribution in Brazil. Int. J. Syst. Evol. Microbiol. 64: 3222-3229

Dall’Agnol, R.F., Ribeiro, R.A., Ormeño-Orrillo, E., Rogel, M.A., Delamuta, J.R.M., Andrade, D.S., Martínez-Romero, E., Hungria, M. (2013) *Rhizobium freirei* sp. nov., a symbiont of *Phaseolus vulgaris* that is very effective at fixing nitrogen. Int. J. Syst. Evol. Microbiol. 63: 4167-4173

Degefu, T., Wolde-Meskel, E., Frostegård, Å. (2013) Phylogenetic diversity of *Rhizobium* strains nodulating diverse legume species growing in Ethiopia. Syst. Appl. Microbiol. 36: 272-280

Flores-Félix, J.D., Ramírez-Bahena, M.H., Salazar, S., Peix, A., Velázquez, E. (2017) Reclassification of *Arthrobacter viscosus* as *Rhizobium viscosum* comb. nov. Int. J. Syst. Evol. Microbiol. doi: 10.1099/ijsem.0.001864

Gao, J-L., Sun, P., Wang, X-M., Lv, F-Y., Mao, X-J., Sun, J-G. (2017) *Rhizobium wenxiniae* sp. nov., an endophytic bacterium isolated from maize root. Int. J. Syst. Evol. Microbiol. 67: 2798-2803

García-Fraile, P., Rivas, R., Willems, A., Peix, A., Martens, M., Martínez-Molina, E., Mateos, P.F., Velázquez, E. (2007) *Rhizobium cellulosilyticum* sp. nov., isolated from sawdust of *Populus alba*. Int. J. Syst. Evol. Microbiol. 57: 844-848

Grison, C.M., Jackson, S., Merlot, S., Dobson, A., Grison, C. (2015) *Rhizobium metallidurans* sp. nov., a symbiotic heavy metal resistant bacterium isolated from the *Anthyllis vulneraria* Zn-hyperaccumulator. Int. J. Syst. Evol. Microbiol. 65: 1525-1530

Grönemeyer, J.L., Kulkarni, A., Berkelmann, D., Hurek, T., Reinhold-Hurek, B. (2014) Rhizobia indigenous to the Okavango region in sub-Saharan Africa: diversity, adaptations, and host specificity. Appl. Environ. Microbiol. 80: 7244-7257

Gu, C.T., Wang, E.T., Tian, C.F., Han, T.X., Chen, W.F., Sui, X.H., Chen, W.X. (2008) *Rhizobium miluonense* sp. nov., a symbiotic bacterium isolated from *Lespedeza* root nodules. Int. J. Syst. Evol. Microbiol. 58: 1364-1368

Gu, T., Sun, L.N., Zhang, J., Sui, X.H., Li, S.P. (2014) *Rhizobium flavum* sp. nov., a triazophos-degrading bacterium isolated from soil under the long-term application of triazophos. Int. J. Syst. Evol. Microbiol. 64: 2017-2022

Han, T.X., Han, L.L., Wu, L.J., Chen, W.F., Sui, X.H., Gu, J.G., Wang, E.T., Chen, W.X. (2008) *Mesorhizobium gobiense* sp. nov. and *Mesorhizobium tarimense* sp. nov., isolated from wild legumes growing in desert soils of Xinjiang, China. Int. J. Syst. Evol. Microbiol. 58: 2610-2618

Hou, B.C., Wang, E.T., Li, Jr. Y., Jia, R.Z., Chen, W.F., Gao, Y., Dong, R.J., Chen, W.X. (2009) *Rhizobium tibeticum* sp. nov., a symbiotic bacterium isolated from *Trigonella archiducis-nicolai* (Širj.) Vassilcz. Int. J. Syst. Evol. Microbiol. 59: 3051-3057

Hunter, W.J., Kuykendall, L.D., Manter, D.K. (2007) *Rhizobium selenireducens* sp. nov.: a selenite-reducing *α-Proteobacteria* isolated from a bioreactor. Curr. Microbiol. 55: 455-460

Jiao, Y.S., Yan, H., Ji, Z.J., Liu, Y.H., Sui, X.H., Wang, E.T., Guo, B.L., Chen, W.X., Chen, W.F. (2015) *Rhizobium sophorae* sp. nov. and *Rhizobium sophoriradicis* sp. nov., nitrogen-fixing rhizobial symbionts of the medicinal legume *Sophora flavescens*. Int. J. Syst. Evol. Microbiol. 65, 497-503

Kaiya, S., Rubaba, O., Yoshida, N., Yamada, T., Hiraishi, A. (2012) Characterization of *Rhizobium naphthalenivorans* sp. nov. with special emphasis on aromatic compound degradation and multilocus sequence analysis of housekeeping genes. J. Gen. Appl. Microbiol. 58: 211-224

Kaur, J., Verma, M., Lal, R. (2011). *Rhizobium rosettiformans* sp. nov., isolated from a hexachlorocyclohexane dump site, and reclassificiation of *Blastobacter* *aggregatus* Hirsch and Müller 1986 as *Rhizobium aggregatum* comb. nov. Int. J. Syst. Evol. Microbiol. 61: 1218-1225

Khalid, R., Zhang, Y.J., Ali, S., Sui, X.H., Zhang, X.X., Amara, U., Chen, W.X., Hayat, R. (2015) *Rhizobium pakistanensis* sp. nov., isolated from groundnut (*Arachis hypogaea*) nodules grown in rainfed Pothwar, Pakistan. Antonie van Leeuwenhoek 107: 281-290

Kittiwongwattana, C., Thawai, C. (2014) *Rhizobium lemnae* sp. nov., a bacterial endophyte of *Lemna aequinoctialis*. Int. J. Syst. Evol. Microbiol. 64: 2455-2460

Lemaire, B., Dlodlo, O., Chimphango, S., Stirton, C., Schrire, B., Boatwright, J.S., Honnay, O., Smets, E., Sprent, J., James, E.K., Muasya, A.M. (2015) Symbiotic diversity, specificity and distribution of rhizobia in native legumes of the Core Cape Subregion (South Africa). FEMS Microbiology Ecology 91. Doi: 10.1093/femsec/fiu024

Lin, D.X., Chen, W.F., Wang, F.Q., Hu, D., Wang, E.T., Sui, X.H., Chen, W.X. (2009) *Rhizobium mesosinicum* sp. nov., isolated from root nodules of trhee different legumes. Int. J. Syst. Evol. Microbiol. 59: 1919-1923

Lin, S-Y., Hsu, Y-H., Liu, Y-C., Hung, M-H., Hameed, A., Lai, W-A., Yen, W-S., Young, C-C. (2014) *Rhizobium straminoryzae* sp. nov., isolated from the surface of rice straw. Int. J. Syst. Evol. Microbiol. 64: 2962-2968

Lin, S-Y., Hung, M-H., Hameed, A., Liu, Y-C., Hsu, Y-H., Wen, C-Z., Arun, A.B., Busse, H-J., Glaeser, S.P., Kämpfer, P., Young, C-C. (2015) *Rhizobium capsici* sp. nov., isolated from root tumor of a green bell pepper (*Capsicum annuum* var. *grossum*) plant. Antonie van Leeuwenhoek 107: 773-784.

Liu, T.Y., Jr. Li, Y., Liu, X.X., Sui, X.H., Zhang, X.X., Wang, E.T., Chen, W.X., Chen, W.F., Puławska, J. (2012) *Rhizobium cauense* sp. nov., isolated from root nodules of the herbaceous legume *Kummerowia stipulacea* grown in campus lawn soil. Syst. Appl. Microbiol. 35: 415-420

Liu, Y., Wang, R-P., Ren, C., Lai, Q-L., Zeng, R-Y. (2015) *Rhizobium marinum* sp. nov., a malachite-green-tolerant bacterium isolated from seawater. Int. J. Syst. Evol. Microbiol. 65: 4449-4454

López-López, A., Rogel, M.A., Ormeño-Orrillo, E., Martínez-Romero, E. (2010) *Phaseolus vulgaris* seed-borne endophytic community with novel bacterial species such as *Rhizobium endophyticum* sp. nov. Syst. Appl. Microbiol. 33: 322-327

Martínez-Romero, E., Segovia, L., Mercante, M., Franco, A.A., Graham, P., Pardo, M.A. (1991) *Rhizobium tropici*, a novel species nodulating *Phaseolus vulgaris* L. beans and *Leucaena* sp. trees. Int. J. Syst. Bacteriol. 41: 417-426

Mnasri, B., Liu, T.Y., Saidi, S., Chen, W.F., Chen, W.X., Zhang, X.X., Mhamdi, R. (2014) *Rhizobium azibense* sp. nov., a nitrogen fixing bacterium isolated from root-nodules of *Phaseolus vulgaris*. Int. J. Syst. Evol. Microbiol. 64: 1501-1506

Parag, B., Sasikala, Ch., Ramana, Ch. V. (2013) Molecular and culture dependent characterization of endolithic bacteria in two beach sand samples and description of *Rhizobium endolithicum* sp. nov. *Antonie van Leeuwenhoek* 104: 1235-1244

Peng, G., Yuan, Q., Li, H., Zhang, W., Tan, Z. (2008) *Rhizobium oryzae* sp. nov., isolated from the wild rice *Oryza alta*. Int. J. Syst. Evol. Microbiol. 58: 2158-2163

Ramana, Ch.V., Parag, B., Girija, K.R., Ram, B.R., Ramana, V.V., Sasikala, Ch. (2013) *Rhizobium subbaraonis* sp. nov., an endolithic bacterium isolated from beach sand. Int. J. Syst. Evol. Microbiol. 63: 581-585

Ramírez-Bahena, M.H., García-Fraile, P., Peix, A., Valverde, A., Rivas, R., Igual, J.M., Mateos, P.F., Martínez-Molina, E., Velázquez, E. (2008) Revision of the taxonomic status of the species *Rhizobium leguminosarum* (Frank 1879) Frank 1889^AL^, *Rhizobium phaseoli* Dangeard 1926^AL^ and *Rhizobium trifolii* Dangeard 1926^AL^. *R. trifolii* is a later synonym of *R. leguminosarum*. Reclassification of the strain *R. leguminosarum* DSM 30132 (=NCIMB 11478) as *Rhizobium pisi* sp. nov. Int. J. Syst. Evol. Microbiol. 58: 2484-2490

Rashid, M.H., Young, J.P.W., Everall, I., Clercx, P., Willems, A., Braun, M.S., Wink, M. (2015) Average nucleotide identity of genome sequences supports the description of *Rhizobium lentis* sp. nov., *Rhizobium bangladeshense* sp. nov. and *Rhizobium binae* sp. nov. from lentil (Lens culinaris) nodules. Int. J. Syst. Evol. Microbiol. 65: 3037-3045

Ren, D.W., Chen, W.F., Sui, X.H., Wang, E.T., Chen, W.X. (2011) *Rhizobium vignae* sp. nov., a symbiotic bacterium isolated from multiple legume species. Int. J. Syst. Evol. Microbiol. 61: 580-586

Ribeiro, R.A., Martins, T.B., Ormeño-Orrillo, E., Delamuta, J.R.M., Rogel, M.A., Martínez-Romero, E., Hungria, M. (2015) *Rhizobium ecuadorense* sp. nov., an indigenous N_2_-fixing symbiont of the Ecuadorian common bean (*Phaseolus vulgaris* L.) genetic pool. Int. J. Syst. Evol. Microbiol. 65: 3162-3169

Ribeiro, R.A., Rogel, M.A., López-López, A., Ormeño-Orrillo, E., Barcellos, F.G., Martínez, J., Thompson, F.L., Martínez-Romero, E., Hungria, M. (2012) Reclassification of *Rhizobium tropici* type A strains as *Rhizobium leucaenae* sp. nov. 62: 1179-1184

Rincón-Rosales, R., Villalobos-Escobedo, J.M., Rogel, M.A., Martinez, J., Ormeño-Orrillo, E., Martínez-Romero, E. (2013) *Rhizobium calliandrae* sp. nov., *Rhizobium mayense* sp. nov. and *Rhizobium jaguaris* sp. nov., rhizobial species nodulating the medicinal legume *Calliandra grandiflora*. Int. J. Syst. Evol. Microbiol. 63: 3423-3429

Román-Ponce, B., Zhang, Y.J., Vásquez-Murrieta, M.S., Sui, X.H., Chen, W.F., Padilla, J.C.A., Guo, X.W., Gao, J.L., Wei, G.H., Wang, E.T. (2016) *Rhizobium acidisoli* sp. nov., isolated from root nodules of *Phaseolus vulgaris* in acid soils. Int. J. Syst. Evol. Microbiol. 66: 398-406

Rozahon, M., Ismayil, N., Hamood, B., Erkin, R., Abdurahman, M., Mamtimin, H., Abdukerim, M., Lal, R., Rahman, E. (2014) *Rhizobium populi* sp. nov., an endophytic bacterium isolated from *Populus euphratica*. Int. J. Syst. Evol. Microbiol. 64: 3215-3221

Saïdi, S., Ramírez-Bahena, M-H., Santillana, N., Zúñiga, D., Álvarez-Martínez, E., Peix, A., Mhamdi, R., Velázquez, E. (2014) *Rhizobium laguerreae* sp. nov. nodulates *Vicia faba* on several continents. Int. J. Syst. Evol. Microbiol. 64: 242-247

Segovia, L., Young, J.P.W., Martínez-Romero, E. (1993) Reclassification of American *Rhizobium leguminosarum* biovar phaseoli type I strains as *Rhizobium etli* sp. nov. Int. J. Syst. Bacteriol. 43: 374-377

Shamseldin, A., Carro, L., Peix, A., Velázquez, E., Moawad, H., Sadowsky, M.J. (2016) The symbiovar trifolii of *Rhizobium bangladeshense* and *Rhizobium* *aegyptiacum* sp. nov. nodulate *Trifolium alexandrinum* in Egypt. Syst. Appl. Microbiol. 39: 275-279

Sheu, S-Y., Chen, Z-H., Young, C-C., Chen, W-M. (2016) *Rhizobium ipomoeae* sp. nov., isolated from a water convolvulus field. Int. J. Syst. Evol. Microbiol. 66: 1633-1640

Sheu, S-Y., Huang, H-W., Young, C-C., Chen, W-M. (2015) *Rhizobium alvei* sp. nov., isolated from a freshwater river. Int. J. Syst. Evol. Microbiol. 65: 472-478

Shi, X., Li, C., Zhao, L., Si, M., Zhu, L., Xin, K., Chen, C., Wang, Y., Shen, X., Zhang, L. (2016) *Rhizobium gei* sp. nov., a bacterial endophyte of *Geum aleppicum*. Int. J. Syst. Evol. Microbiol. 66: 4282-4288

Squartini, A., Struffi, P., Döring, H., Selenska-Pobell, S., Tola, E., Giacomini, A., Vendramin, E., Velázquez, E., Mateos, P.F., Martínez-Molina, E., Dazzo, F.B., Casella, S., Nuti, M.P. (2002) *Rhizobium sullae* sp. nov. (formerly ‘*Rhizobium hedysari*’), the root-nodule microsymbiont of *Hedysarum coronarium* L. Int. J. Syst. Evol. Microbiol. 52: 1267-1276

Tan, Z.Y., Kan, F.L., Peng, G.X., Wang, E.T., Reinhold-Hurek, B., Chen, W.X. (2001) *Rhizobium yanglingense* sp. nov., isolated from arid and semi-arid regions in China. Int. J. Syst. Evol. Microbiol. 51: 909-914

Tejerizo, G.T., Rogel, M.A., Ormeño-Orrillo, E., Althabegoiti, M.J., Nilsson, J.F., Niehaus, K., Schlüter, A., Pühler, A., Del Papa, M.F., Lagares, A., Martínez-Romero, E., Pistorio, M. (2016) *Rhizobium favelukesii* sp. nov., isolated from the root nodules of alfalfa (*Medicago sativa* L). Int. J. Syst. Evol. Microbiol. 66: 4451-4457

Tian, C.F., Wang, E.T., Wu, L.J., Han, T.X., Chen, W.F., Gu, C.T., Gu, J.G., Chen, W.X. (2008) *Rhizobium fabae* sp. nov., a bacterium that nodulates *Vicia faba*. Int. J. Syst. Evol. Microbiol. 58: 2871-2875

Turdahon, M., Osman, G., Hamdun, M., Yusuf, K., Abdurehim, Z., Abaydulla, G., Abdukerim, M., Fang, C., Rahman, E. (2013) *Rhizobium tarimense* sp. nov., isolated from soil in the ancient Khiyik River. Int. J. Syst. Evol. Microbiol. 63: 2424-2429

Valverde, A., Igual, J.M., Peix, A., Cervantes, E., Velázquez, E. (2006) *Rhizobium lusitanum* sp. nov. a bacterium that nodulates *Phaseolus vulgaris*. Int. J. Syst. Evol. Microbiol. 56: 2631-2637

Van Berkum, P., Beyene, D., Bao, G., Campbell, A., Eardly, B.D. (1998) *Rhizobium mongolense* sp. nov. is one of three rhizobial genotypes identified which nodulate and form nitrogen-fixing symbioses with *Medicago ruthenica* [(L.) Ledebour]. Int. J. Syst. Bacteriol. 48: 13-22

Wang, F., Wang, E.T., Wu, L.J., Sui, X.H., Li, Jr, Y., Chen, W.X. (2011) *Rhizobium vallis* sp. nov., isolated from nodules of three leguminous species. Int. J. Syst. Evol. Microbiol. 61: 2582-2588

Wei, G.H., Tan, Z.Y., Zhu, M.E., Wang, E.T., Han, S.Z., Chen, W.X. (2003) Characterization of rhizobia isolated from legume species within the genera *Astragalus* and *Lespedeza* grown in the Loess Plateau of China and description of *Rhizobium loessense* sp. nov. Int. J. Syst. Evol. Microbiol. 53: 1575-1583

Wei, G.H., Wang, E.T., Tan, Z.Y., Zhu, M.E., Chen, W.X. (2002) *Rhizobium indigoferae* sp. nov. and *Sinorhizobium kummerowiae* sp. nov., respectively isolated from *Indigofera* spp. and *Kummerowia stipulacea*. Int. J. Syst. Evol. Microbiol. 52: 2231-2239

Wei, X., Yan, S., Li, D., Pang, H., Li, Y., Zhang, J. (2015) *Rhizobium helianthi* sp. nov., isolated from the rhizosphere of sunflower. Int. J. Syst. Evol. Microbiol. 65: 4455-4460

Xu, L., Shi, J., Li, C., Zhu, S., Li, B. (2017) *Rhizobium hedysari* sp. nov., a novel species isolated from a root nodule of *Hedysarum multijugum* in China. Antonie van Leeuwenhoek doi: 10.1007/s10482-016-0817-z

Yan, J., Yan, H., Liu, L.X., Chen, W.F., Zhang, X.X., Verástegui-Valdés, M.M., Wang, E.T., Han, X.Z. (2017) *Rhizobium hidalgonense* sp. nov., a nodule endophytic bacterium of Phaseolus vulgaris in acid soil. Arch. Microbiol. 199: 97-104

Yoon, J-H., Kang, S-J., Yi, H-S., Oh, T-K., Ryu, C-M. (2010) *Rhizobium soli* sp. nov., isolated from soil. Int. J. Syst. Evol. Microbiol. 60: 1387-1393

Young, J.M., Kuykendall, L.D., Martínez-Romero, E., Kerr, A., Sawada, H. (2001) A revision of *Rhizobium* Frank 1889, with an emended description of the genus, and the inclusion of all species of *Agrobacterium* Conn 1942 and *Allorhizobium undicola* de Lajudie et al. 1998 as new combinations: *Rhizobium radiobacter*, *R. rhizogenes*, *R. rubi*, *R. undicola* and *R. vitis*. Int. J. Syst. Evol. Microbiol. 51: 89-103

Zhang, R.J., Hou, B.C., Wang, E.T., Li, Jr, Y., Zhang, X.X., Chen, W.X. (2011) *Rhizobium tubonense* sp. nov., isolated from root nodules of *Oxytropis glabra*. Int. J. Syst. Evol. Microbiol. 61: 512-517

Zhang, X., Li, B., Wang, H., Sui, X., Ma, X., Hong, Q., Jiang, R. (2012) *Rhizobium petrolearium* sp. nov., isolated from oil-contaminated soil. Int. J. Syst. Evol. Microbiol. 62: 1871-1876

Zhang, L., Shi, X., Si, M., Li, C., Zhu, L., Zhao, L., Shen, X., Wang, Y. (2014b) *Rhizobium smilacinae* sp. nov., an endophytic bacterium isolated from the leaf of *Smilacina japonica*. Antonie van Leeuwenhoek 106: 715-723

Zhang, S., Yang, S., Chen, W., Chen, Y., Zhang, M., Zhou, X., Fan, G., Feng, F.Y. (2017) *Rhizobium arenae* sp. nov., isolated from the sand of Desert Mu Us. China. Int. J. Syst. Evol. Microbiol. doi: 10.1099/ijsem.0.001810

Zhang, X.X., Gao, J-S., Cao, Y-H., Sheirdil, R.A., Wang, X-C., Zhang, L. (2015) *Rhizobium oryzicola* sp. nov., potential plant-growth-promoting endophytic bacteria isolated from rice roots. Int. J. Syst. Evol. Microbiol. 65: 2931-2936

Zhang, X-X., Tang, X., Sheirdil, R.A., Sun, L., Ma, X-T. (2014a) *Rhizobium rhizoryzae* sp. nov., isolated from rice roots. Int. J. Syst. Evol. Microbiol. 64: 1373-1377

Zhao, J-J., Zhang, J., Sun, L., Zhang, R-J., Zhang, C-W., Yin, H-Q., Zhang, X-X. (2017a) *Rhizobium oryziradicis* sp. nov., isolated from rice roots. Int. J. Syst. Evol. Microbiol. 67: 963-968

Zhao, J-J., Zhang, J., Zhang, R-J., Zhang, C-W., Yin, H-Q., Zhang, X-X. (2017b) *Rhizobium rhizosphaerae* sp. nov., a novel species isolated from rice rhizosphere. Antonie van Leeuwenhoek doi: 10.1007/s10482-017-0831-9
