# Supplementary material for: Both Alpha- and Beta-Rhizobia Occupy the Root Nodules of Vachellia karroo in South Africa
Source: Front Microbiol. 2019 Jun 4;10:1195. doi: 10.3389/fmicb.2019.01195 (PMC6558075; doi:10.3389/fmicb.2019.01195)
Supplement: Supplementary file 5 [file Table_5.DOCX]

**Supplementary Table S5.** Isolate names, accession numbers, host/niche, country of origin and references for the *Bradyrhizobium* isolates used in this study

| **Isolate** | ***RecA*** | **Host/Niche** | **Country** | **Reference** |
| --- | --- | --- | --- | --- |
| *B. americanum* CMVU44^T^ | KC247141 | *Centrosema macrocarpum* | Venezuela | Ramírez-Bahena et al., 2016 |
| *B. arachidis* CCBAU051107^T^ | HM107233 | *Arachis hypogaea* | China | Wang et al., 2013 |
| *B. betae* PL7HG1^T^ | FJ970378 | *Beta vulgaris* | Spain | Rivas et al., 2004 |
| *B. brasilense* UFLA03-321^T^ | KT793142 | *Vigna unguiculata* | Brazil | Martins da Costa et al., 2017 |
| *B. canariense* BTA-1^T^ | AY591553 | *Chamaecytisus proliferus* | Canary Islands | Vinuesa et al., 2005 |
| *B. centrolobii* BR10245^T^ | AYJ54_21215 | *Centrolobium paraense* | Brazil | Michel et al., 2017 |
| *B. centrosemae* A9^T^ | KC247145 | *Centrosema molle* | Venezuela | Ramírez-Bahena et al., 2016 |
| *B. cytisi* CTAW11^T^ | GU001575 | *Cytisus villosus* | Morocco | Chahboune et al., 2011 |
| *B. daqingense* CCBAU15774^T^ | HQ231270 | *Glycine max* | China | Wang et al., 2012 |
| *B. denitrificans* LMG8443^T^/KIS30-44^T^ | EU665419 | Surface water | Germany | Van Berkum et al., 2006 |
| *B. diazoefficiens* USDA110^T^ | bll5755 | *Glycine max* | USA | Delamuta et al., 2013 |
| *B. elkanii* USDA76^T^ | KF532941 | *Glycine max* | Japan | Kuykendall et al., 1992 |
| *B. embrapense* SEMIA6208^T^ | AKN83_RS12090 | *Desmodium heterocarpon* | Colombia | Delamuta et al., 2015 |
| *B. erythrophlei* CCBAU53325^T^ | KF114669 | *Erythrophleum fordii* | China | Yao et al., 2015 |
| *B. ferriligni* CCBAU51502^T^ | KJ818112 | *Erythrophleum fordii* | China | Yao et al., 2015 |
| *B. ganzhouense* RITF806^T^ | JX277144 | *Acacia melanoxylon* | China | Lu et al., 2014 |
| *B. guangdongense* CCBAU51649^T^ | KC509269 | *Arachis hypogaea* | China | Li et al., 2015 |
| *B. guangxiense* CCBAU53363 | KC509279 | *Arachis hypogaea* | China | Li et al., 2015 |
| *B. huanghuaihaiense* CCBAU23303^T^ | HQ231595 | *Glycine max* | China | Zhang et al., 2012 |
| *B. icense* LMTR13^T^ | JX943615 | *Phaseolus lunatus* | Peru | Durán et al., 2014a |
| *B. ingae* BR10250^T^ | KF927061 | *Inga laurina* | Brazil | Da Silva et al., 2014 |
| *B. iriomotense* EK05^T^ | AB300996 | *Entada koshunensis* | Japan | Islam et al., 2008 |
| *B. japonicum* USDA6^T^ | BJ6T_39800 | *Glycine max* | Japan | Jordan, 1982 |
| *B. jicamae* PAC68^T^ | LM994324 | *Pachyrhizus erosus* | Honduras | Ramírez-Bahena et al., 2009 |
| *B. kavangense* 14-3^T^ | KM378399 | *Vigna unguiculata* | Namibia | Grönemeyer et al., 2015a |
| *B. lablabi* CCBAU23086^T^ | LM994322 | *Lablab purpureus* | China | Chang et al., 2011 |
| *B. liaoningense* 2281^T^ | FM253180 | *Glycine max* | China | Xu et al., 1995 |
| *B. lupini* USDA3051^T^ | KM114866 | *Lupinus* sp. | New Zealand | Peix et al., 2015 |
| *B. macuxiense* BR10303^T^ | AS156_28455 | *Centrolobium paraense* | Brazil | Michel et al., 2017 |
| *B. manausense* BR3351^T^ | KF785992 | *Vigna unguiculata* | Brazil | Silva et al., 2014 |
| *B. neotropicale* BR10247^T^ | KJ661714 | *Centrolobium paraense* | Brazil | Zilli et al., 2014 |
| *B. oligotrophicum* LMG10732^T^ | JQ619231 | Rice paddy soil | Japan | Ramírez-Bahena et al., 2013 |
| *B. ottawaense* OO99^T^ | HQ587287 | *Glycine max* | Canada | Yu et al., 2014 |
| *B. pachyrhizi* PAC48^T^ | LM994323 | *Pachyrhizus erosus* | Costa Rica | Ramírez-Bahena et al., 2009 |
| *B. paxllaeri* LMTR21^T^ | JX943617 | *Phaseolus lunatus* | Peru | Durán et al., 2014a |
| *B. retamae* Ro19^T^ | LM994318 | *Retama monosperma* | Morocco | Guerrouj et al., 2013 |
| *B. rifense* CTAW71^T^ | GU001585 | *Cytisus villosus* | Morocco | Chahboune et al., 2012 |
| *B. stylosanthis* BR446^T^ | KU724163 | *Stylosanthes guianensis* | Brazil | Delamuta et al., 2016 |
| *B. subterraneum* 58 2-1^T^ | KM378397 | *Arachis hypogaea* | Namibia | Grönemeyer et al., 2015b |
| *B. tropiciagri* SEMIA6148^T^ | AKN84_RS27210 | *Neonotonia wightii* | Brazil | Delamuta et al., 2015 |
| *B. valentinum* LmjM3^T^ | JX518589 | *Lupinus mariae-josephae* | Spain | Durán et al., 2014b |
| *B. vignae* CCBAU05176^T^ | KM378374 | *Vigna unguiculata* | Namibia | Grönemeyer et al., 2016 |
| *B. viridifuturi* SEMIA690^T^ | ALC15_RS22445 | *Centrosema pubescens* | Brazil | Ferraz Helene et al., 2015 |
| *B. yuanmingense* B071^T^ | AY591566 | *Lespedeza cuneata* | China | Yao et al., 2002 |
| *B.* genosp. α BC-C1 | AY591540 | *Chamaecytisus proliferus* | Canary Islands | Jarabo-Lorenzo et al., 2003 |
| *B.* sp. BGA-1 | AY591558 | *Teline stenopetala* | Canary Islands | Jarabo-Lorenzo et al., 2003 |
| *B.* genosp. β BRE-1 | AY591551 | *Teline canariensis* | Spain | Vinuesa et al., 2005 |
| *B.* sp. MM5621 | KF802770 | *Indigofera gracilis* | South Africa | Lemaire et al., 2015 |
| *B.* sp. CH2437 | AM168327 | *Lupinus tominensis* | Bolivia | Stępkowski et al., 2007 |
| *B.* sp. GHa | EU364681 | *Vigna unguiculata* | Botswana | Steenkamp et al., 2008 |
| *B.* sp. GHx | EU364684 | *Vigna unguiculata* | Botswana | Steenkamp et al., 2008 |
| *B.* sp. GHvi | EU364683 | *Vigna unguiculata* | Botswana | Steenkamp et al., 2008 |
| *B.* sp. GHiv | EU364682 | *Vigna unguiculata* | Botswana | Steenkamp et al., 2008 |
| *B.* sp. CH2509 | AM168332 | *Lupinus albescens* | Brazil | Stępkowski et al., 2007 |
| *B.* sp. GC1d | EU364680 | *Vigna unguiculata* | Botswana | Steenkamp et al., 2008 |
| *B.* sp. CB756 | BRAGE_RS0107165 | *Macrotyloma africanum* | Zimbabwe | Steenkamp et al., 2008 |
| *B.* sp. RP7b | EU364696 | *Arachis hypogaea* | South Africa | Steenkamp et al., 2008 |
| *B.* sp. BM25 | EU364675 | *Vigna unguiculata* | Botswana | Steenkamp et al., 2008 |
| *B.* sp. RC2d | EU364693 | *Vigna unguiculata* | South Africa | Steenkamp et al., 2008 |
| *B.* sp. RP6b | EU364695 | *Arachis hypogaea* | South Africa | Steenkamp et al., 2008 |
| *B.* sp. R8 | EU364689 | *Vigna unguiculata* | Botswana | Steenkamp et al., 2008 |
| *B.* sp. GP2e | EU364685 | *Arachis hypogaea* | Botswana | Steenkamp et al., 2008 |
| *B.* sp. FP4f | EU364679 | *Arachis hypogaea* | Botswana | Steenkamp et al., 2008 |
| *B.* sp. BM1 | EU364674 | *Vigna unguiculata* | Botswana | Steenkamp et al., 2008 |
| *B.* sp. FC1b | EU364677 | *Vigna unguiculata* | Botswana | Steenkamp et al., 2008 |
| *B.* sp. FP1c | EU364678 | *Arachis hypogaea* | Botswana | Steenkamp et al., 2008 |
| *B.* sp. RC3b | EU364694 | *Vigna unguiculata* | South Africa | Steenkamp et al., 2008 |
| *B.* sp. ISLU16 | AY591576 | *Ornithopus compressus* | Spain | Jarabo-Lorenzo et al., 2003 |
| *B.* sp. BLUT1 | AM168322 | *Lupinus albus* | Canary Islands | Stępkowski et al., 2007 |
| *B.* sp. WSM2632 | KM247868 | *Lotononis* sp. | South Africa | Ardley et al., 2013 |
| *B.* sp. WSM2783 | YY7_RS0112325 | *Leobordea carinata* | South Africa | Ardley et al., 2013 |
| *B.* sp. MM5405 | KF802795 | *Tephrosia capensis* | South Africa | Lemaire et al., 2015 |
| *B.* sp. MM5392 | KF802769 | *Indigofera frutescens* | South Africa | Lemaire et al., 2015 |
| *B.* sp. WSM2596 | KM247867 | *Leobordea foliosa* | South Africa | Ardley et al., 2013 |
| *B.* sp. R5 | EU364688 | *Vigna unguiculata* | Botswana | Steenkamp et al., 2008 |
| *B.* sp. R10 | EU364691 | *Vigna unguiculata* | Botswana | Steenkamp et al., 2008 |
| *B.* sp. R10m | EU364692 | *Vigna unguiculata* | Botswana | Steenkamp et al., 2008 |
| *B.* sp. R3 | EU364687 | *Vigna unguiculata* | Botswana | Steenkamp et al., 2008 |
| *B.* sp. R2m | EU364686 | *Vigna unguiculata* | Botswana | Steenkamp et al., 2008 |
| *B.* sp. R8m | EU364690 | *Vigna unguiculata* | Botswana | Steenkamp et al., 2008 |
| *B.* sp. 26-1-1 | KM378393 | *Arachis hypogaea* | Namibia | Grönemeyer et al., 2014 |
| *B.* sp. 28 2-1 | KM378419 | *Arachis hypogaea* | Namibia | Grönemeyer et al., 2014 |
| *B.* sp. 10-2 | KM378376 | *Vigna unguiculata* | Namibia | Grönemeyer et al., 2014 |
| *B.* sp. 21 1-1 | KM378408 | *Vigna unguiculata* | Namibia | Grönemeyer et al., 2014 |
| *B.* sp. B14 6-1 | KM378412 | *Vigna subterranea* | Namibia | Grönemeyer et al., 2014 |
| *B.* sp. 16-10 | KM378405 | *Arachis hypogaea* | Namibia | Grönemeyer et al., 2014 |
| *B.* sp. 36 3-2 | KM378409 | *Vigna subterranea* | Namibia | Grönemeyer et al., 2014 |
| *B.* sp. 1B 2-1 | KM378386 | *Vigna subterranea* | Namibia | Grönemeyer et al., 2014 |
| *B.* sp. 1B 1-1 | KM378417 | *Vigna subterranea* | Namibia | Grönemeyer et al., 2014 |
| *B.* sp. B14 4-2 | KM378387 | *Vigna subterranea* | Namibia | Grönemeyer et al., 2014 |
| *B.* sp. 3B 4-1 | KM378418 | *Vigna subterranea* | Namibia | Grönemeyer et al., 2014 |
| *B.* sp. 27 1-1 | KM378415 | *Arachis hypogaea* | Namibia | Grönemeyer et al., 2014 |
| *B.* sp. 9-5 | KM378375 | *Vigna subterranea* | Namibia | Grönemeyer et al., 2014 |
| *B.* sp. 35 1-3 | KM378378 | *Vigna unguiculata* | Namibia | Grönemeyer et al., 2014 |
| *B.* sp. 3-2 | KM378392 | *Vigna unguiculata* | Namibia | Grönemeyer et al., 2014 |
| *B.* sp. 4-8 | KM378406 | *Vigna unguiculata* | Namibia | Grönemeyer et al., 2014 |
| *B.* sp. 1-7 | KM378372 | *Arachis hypogaea* | Namibia | Grönemeyer et al., 2014 |
| *B.* sp. 6-8 | KM378394 | *Vigna unguiculata* | Namibia | Grönemeyer et al., 2014 |
| *B.* sp. 2-13 | KM378373 | *Arachis hypogaea* | Namibia | Grönemeyer et al., 2014 |
| *B.* sp. 60 2-1 | KM378385 | *Vigna subterranea* | Namibia | Grönemeyer et al., 2014 |
| *B.* sp. 54 1-1 | KM378395 | *Vigna subterranea* | Namibia | Grönemeyer et al., 2014 |
| *B.* sp. 57 2-1 | KM378410 | *Arachis hypogaea* | Namibia | Grönemeyer et al., 2014 |
| *B.* sp. 55 1-1 | KM378402 | *Vigna subterranea* | Namibia | Grönemeyer et al., 2015b |
| *B.* sp. 36-1-1 | KM378400 | *Vigna subterranea* | Nambia | Grönemeyer et al., 2014 |
| *B.* sp. 15B | LN890752 | *Vachellia karroo* | South Africa | This study |
| *B.* sp. 30-1-1 | KM378423 | *Lablab purpureus* | Namibia | Grönemeyer et al., 2014 |
| *B.* sp. 32-2-1 | KM378424 | *Lablab purpureus* | Namibia | Grönemeyer et al., 2014 |
| *B.* sp. 21B-2-1 | KM378414 | *Vigna subterranea* | Namibia | Grönemeyer et al., 2014 |
| *B.* sp. 31-1-1 | KM378382 | *Lablab purpureus* | Namibia | Grönemeyer et al., 2014 |
| *B.* sp. 30-2-1 | KM378401 | *Lablab purpureus* | Namibia | Grönemeyer et al., 2014 |
| *B.* sp. 37-1-1 | KM378411 | *Vigna subterranae* | Namibia | Grönemeyer et al., 2014 |
| *B.* sp. 35 3-3 | KM378379 | *Vigna unguiculata* | Namibia | Grönemeyer et al., 2014 |
| *B.* sp. 35-3-4 | KM378380 | *Vigna unguiculata* | Namibia | Grönemeyer et al., 2014 |
| *B.* sp. 34 1-1 | KM378407 | *Vigna unguiculata* | Namibia | Grönemeyer et al., 2014 |
| *B.* sp. 45-1-3 | KM378427 | *Vigna unguiculata* | Angola | Grönemeyer et al., 2014 |
| *B.* sp. 45-1-4 | KM378428 | *Vigna unguiculata* | Angola | Grönemeyer et al., 2014 |
| *B.* sp. 22-2-1 | KM378391 | *Vigna unguiculata* | Namibia | Grönemeyer et al., 2014 |
| *B.* sp. 30-3-3 | KM378381 | *Lablab purpureus* | Namibia | Grönemeyer et al., 2014 |
| *B.* sp. 30 3-2 | KM378420 | *Lablab purpureus* | Namibia | Grönemeyer et al., 2014 |
| *B.* sp. 5-11 | KM378422 | *Lablab purpureus* | Namibia | Grönemeyer et al., 2014 |
| *B.* sp. 5-10 | KM378377 | *Lablab purpureus* | Namibia | Grönemeyer et al., 2014 |
| *B.* sp. 18C-1-1 | KM378434 | *Vigna unguiculata* | Namibia | Grönemeyer et al., 2014 |
| *B.* sp. P14-1-1 | KM378435 | *Vigna subterranea* | Angola | Grönemeyer et al., 2014 |
| *B.* sp. 47-1-1 | KM378404 | *Vigna unguiculata* | Angola | Grönemeyer et al., 2014 |
| *B.* sp. 51-1-3 | KM378430 | *Vigna unguiculata* | Angola | Grönemeyer et al., 2014 |
| B. sp. F1-2-1 | KM378398 | *Vigna subterranea* | Angola | Grönemeyer et al., 2014 |
| *B.* sp. 49-2-1 | KM378421 | *Phaseolus vulgaris* | Angola | Grönemeyer et al., 2014 |
| *B.* sp. F1-1-1 | KM378389 | *Vigna subterranea* | Angola | Grönemeyer et al., 2014 |
| *B.* sp. 46-1-1 | KM378429 | *Vigna unguiculata* | Angola | Grönemeyer et al., 2014 |
| *B.* sp. 40-3-1 | KM378384 | *Vigna unguiculata* | Angola | Grönemeyer et al., 2014 |
| *B.* sp. 42-1-1 | KM378403 | *Phaseolus vulgaris* | Angola | Grönemeyer et al., 2014 |
| *B.* sp. 40-2-2 | KM378383 | *Vigna unguiculata* | Angola | Grönemeyer et al., 2014 |
| *B.* sp. 44-1-1 | KM378426 | *Vigna unguiculata* | Angola | Grönemeyer et al., 2014 |
| *B.* sp. 41-1-2 | KM378431 | *Vigna unguiculata* | Angola | Grönemeyer et al., 2014 |
| *B.* sp. AD1T2-3-1 | KM378390 | *Vigna subterranea* | Angola | Grönemeyer et al., 2014 |
| *B.* sp. 26-3-1 | KM378433 | *Arachis hypogaea* | Namibia | Grönemeyer et al., 2014 |
| *B.* sp. 18C-2-1 | KM378388 | *Vigna unguiculata* | Namibia | Grönemeyer et al., 2014 |
| *B.* sp. Arg62 | LN650151 | *Argyrolobium sericeum* | South Africa | Beukes et al., 2016 |
| *B.* sp. Arg68 | LN650152 | *Argyrolobium sericeum* | South Africa | Beukes et al., 2016 |
| *B.* sp. Arg105 | LN650153 | *Argyrolobium rupestre* | South Africa | Beukes et al., 2016 |
| *B.* sp. Leo20 | LN650154 | *Leobordea pulchra* | South Africa | Beukes et al., 2016 |
| *B.* sp. Leo78 | LN650155 | *Leobordea pulchra* | South Africa | Beukes et al., 2016 |
| *B.* sp. Leo79 | LN650156 | *Leobordea pulchra* | South Africa | Beukes et al., 2016 |
| *B.* sp. Leo84 | LN650157 | *Leobordea pulchra* | South Africa | Beukes et al., 2016 |
| *B.* sp. Leo132 | LN650161 | *Leobordea pulchra* | South Africa | Beukes et al., 2016 |
| *B.* sp. Leo142 | LN650162 | *Leobordea pulchra* | South Africa | Beukes et al., 2016 |
| *B.* sp. Leo166 | LN650163 | *Leobordea pulchra* | South Africa | Beukes et al., 2016 |
| *B.* sp. Leo114 | LN650158 | *Leobordea divaricata* | South Africa | Beukes et al., 2016 |
| *B.* sp. Leo117 | LN650159 | *Leobordea divaricata* | South Africa | Beukes et al., 2016 |
| *B.* sp. Leo121 | LN650160 | *Leobordea divaricata* | South Africa | Beukes et al., 2016 |
| *B.* sp. Leo170 | LN650164 | *Leobordea lanceolata* | South Africa | Beukes et al., 2016 |
| *B.* sp. Leo176 | LN650165 | *Leobordea lanceolata* | South Africa | Beukes et al., 2016 |
| *B.* sp. Pear76 | LN650166 | *Pearsonia obovata* | South Africa | Beukes et al., 2016 |
| *B.* sp. Pear77 | LN650167 | *Pearsonia obovata* | South Africa | Beukes et al., 2016 |
| *B.* sp. Pear128 | LN650168 | *Pearsonia obovata* | South Africa | Beukes et al., 2016 |
| *B.* sp. Pear129 | LN650169 | *Pearsonia obovata* | South Africa | Beukes et al., 2016 |
| *B.* sp. Cham227 | LN650170 | *Chamaecrista* sp. | South Africa | Beukes et al., 2016 |
| *B.* sp. Cham231 | LN650171 | *Chamaecrista* sp. | South Africa | Beukes et al., 2016 |
| *B.* sp. LmicAL48 | KU752981 | *Lupinus micranthus* | Spain | Bourebaba et al., 2016 |
| *B.* sp. LmicZ10 | KU752980 | *Lupinus micranthus* | Algeria | Bourebaba et al., 2016 |
| *B.* sp. LmicT8 | KU557389 | *Lupinus micranthus* | Algeria | Bourebaba et al., 2016 |
| *B.* sp. LmicT5 | KU753002 | *Lupinus micranthus* | Algeria | Bourebaba et al., 2016 |
| *B.* sp. LmicT28 | KU752976 | *Lupinus micranthus* | Algeria | Bourebaba et al., 2016 |
| *B.* sp. LmicT26 | KU752974 | *Lupinus micranthus* | Algeria | Bourebaba et al., 2016 |
| *B.* sp. LmicT18 | KU753005 | *Lupinus micranthus* | Algeria | Bourebaba et al., 2016 |
| *B.* sp. LmicT16 | KU752996 | *Lupinus micranthus* | Algeria | Bourebaba et al., 2016 |
| *B.* sp. LmicT14 | KU752995 | *Lupinus micranthus* | Algeria | Bourebaba et al., 2016 |
| *B.* sp. LmicT13 | KU752994 | *Lupinus micranthus* | Algeria | Bourebaba et al., 2016 |
| *B.* sp. LmicT10 | KU752993 | *Lupinus micranthus* | Algeria | Bourebaba et al., 2016 |
| *B.* sp. LmicISLU122 | KU753003 | *Lupinus micranthus* | Spain | Bourebaba et al., 2016 |
| *B.* sp. LmicAM6 | KU752971 | *Lupinus micranthus* | Algeria | Bourebaba et al., 2016 |
| *B.* sp. LmicAM3 | KU752988 | *Lupinus micranthus* | Algeria | Bourebaba et al., 2016 |
| *B.* sp. LmicF65 | KU753004 | *Lupinus micranthus* | Spain | Bourebaba et al., 2016 |
| *B.* sp. LmicF63 | KU752977 | *Lupinus micranthus* | Spain | Bourebaba et al., 2016 |
| *B.* sp. LmicF61 | KU752985 | *Lupinus micranthus* | Spain | Bourebaba et al., 2016 |
| *B.* sp. LmicAL42 | KU752972 | *Lupinus micranthus* | Spain | Bourebaba et al., 2016 |
| *B.* sp. LmicAL38 | KU752978 | *Lupinus micranthus* | Spain | Bourebaba et al., 2016 |
| *B.* sp. LmicAL32 | KU752982 | *Lupinus micranthus* | Spain | Bourebaba et al., 2016 |
| *B.* sp. LmicAL27 | KU752986 | *Lupinus micranthus* | Spain | Bourebaba et al., 2016 |
| *B.* sp. LmicM12 | KU752984 | *Lupinus micranthus* | Spain | Bourebaba et al., 2016 |
| *B.* sp. LmicISLU13 | KU753006 | *Lupinus micranthus* | Spain | Bourebaba et al., 2016 |
| *B.* sp. LmicZ4 | KU753001 | *Lupinus micranthus* | Algeria | Bourebaba et al., 2016 |
| *B.* sp. LmicZ3 | KU753000 | *Lupinus micranthus* | Algeria | Bourebaba et al., 2016 |
| *B.* sp. LmicT27 | KU752975 | *Lupinus micranthus* | Algeria | Bourebaba et al., 2016 |
| *B.* sp. LmicT2 | KU752997 | *Lupinus micranthus* | Algeria | Bourebaba et al., 2016 |
| *B.* sp. LmicT3 | KU752998 | *Lupinus micranthus* | Algeria | Bourebaba et al., 2016 |
| *B.* sp. LmicIA7 | KU752992 | *Lupinus micranthus* | Algeria | Bourebaba et al., 2016 |
| *B.* sp. LmicIA4 | KU752991 | *Lupinus micranthus* | Algeria | Bourebaba et al., 2016 |
| *B.* sp. LmicIA1 | KU752990 | *Lupinus micranthus* | Algeria | Bourebaba et al., 2016 |
| *B.* sp. LmicAM4 | KU752989 | *Lupinus micranthus* | Algeria | Bourebaba et al., 2016 |
| *B.* sp. LmicM10 | KU752983 | *Lupinus micranthus* | Spain | Bourebaba et al., 2016 |
| *B.* sp. LmicM20 | KU752970 | *Lupinus micranthus* | Spain | Bourebaba et al., 2016 |
| *B.* sp. LmicAL52 | KU752979 | *Lupinus micranthus* | Spain | Bourebaba et al., 2016 |
| *B.* sp. LmicAL54 | KU752973 | *Lupinus micranthus* | Spain | Bourebaba et al., 2016 |
| *B.* sp. LmicA16 | KU752987 | *Lupinus micranthus* | Algeria | Bourebaba et al., 2016 |
| *B.* sp. LmiT6 | KX272829 | *Lupinus micranthus* | Tunisia | Msaddak et al., 2017 |
| *B.* sp. LmiT7 | KX272830 | *Lupinus micranthus* | Tunisia | Msaddak et al., 2017 |
| *B.* sp. LmiT3 | KX272827 | *Lupinus micranthus* | Tunisia | Msaddak et al., 2017 |
| *B.* sp. LmiT1c | KX272826 | *Lupinus micranthus* | Tunisia | Msaddak et al., 2017 |
| *B.* sp. LmiT15 | KX272828 | *Lupinus micranthus* | Tunisia | Msaddak et al., 2017 |
| *B.* sp. LmiT14 | KX272837 | *Lupinus micranthus* | Tunisia | Msaddak et al., 2017 |
| *B.* sp. LmiT13 | KX272836 | *Lupinus micranthus* | Tunisia | Msaddak et al., 2017 |
| *B.* sp. LmiT12 | KX272835 | *Lupinus micranthus* | Tunisia | Msaddak et al., 2017 |
| *B.* sp. LmiT11 | KX272834 | *Lupinus micranthus* | Tunisia | Msaddak et al., 2017 |
| *B.* sp. LmiT10 | KX272833 | *Lupinus micranthus* | Tunisia | Msaddak et al., 2017 |
| *B.* sp. LmiM17 | KX272825 | *Lupinus micranthus* | Tunisia | Msaddak et al., 2017 |
| *B.* sp. LmiM15 | KX272824 | *Lupinus micranthus* | Tunisia | Msaddak et al., 2017 |
| *B.* sp. LmiM11 | KX272823 | *Lupinus micranthus* | Tunisia | Msaddak et al., 2017 |
| *B.* sp. LmiT20 | KX272839 | *Lupinus micranthus* | Tunisia | Msaddak et al., 2017 |
| *B.* sp. LmiT8 | KX272831 | *Lupinus micranthus* | Tunisia | Msaddak et al., 2017 |
| *B.* sp. LmiT9 | KX272832 | *Lupinus micranthus* | Tunisia | Msaddak et al., 2017 |
| *B.* sp. LmiB16 | KX272816 | *Lupinus micranthus* | Tunisia | Msaddak et al., 2017 |
| *B.* sp. LmiB5 | KX272815 | *Lupinus micranthus* | Tunisia | Msaddak et al., 2017 |
| *B.* sp. LmiB2 | KX272812 | *Lupinus micranthus* | Tunisia | Msaddak et al., 2017 |
| *B.* sp. LmiB3 | KX272813 | *Lupinus micranthus* | Tunisia | Msaddak et al., 2017 |
| *B.* sp. LmiB4 | KX272814 | *Lupinus micranthus* | Tunisia | Msaddak et al., 2017 |
| *B.* sp. LmiM2 | KX272821 | *Lupinus micranthus* | Tunisia | Msaddak et al., 2017 |
| *B.* sp. LmiM3 | KX272822 | *Lupinus micranthus* | Tunisia | Msaddak et al., 2017 |
| *B.* sp. LmiM1 | KX272820 | *Lupinus micranthus* | Tunisia | Msaddak et al., 2017 |
| *B.* sp. LmiH2 | KX272817 | *Lupinus micranthus* | Tunisia | Msaddak et al., 2017 |
| *B.* sp. LmiH3 | KC272818 | *Lupinus micranthus* | Tunisia | Msaddak et al., 2017 |
| B. sp. LmiH4 | KX272819 | *Lupinus micranthus* | Tunisia | Msaddak et al., 2017 |
| *B.* sp. F86-1-3 | KR232255 | *Faidherbia albida* | Mozambique | Teixeira and Rodríguez-Echeverría, 2016 |
| *B.* sp. F82-2-4 | KR232237 | *Faidherbia albida* | Mozambique | Teixeira and Rodríguez-Echeverría, 2016 |
| *B.* sp. Alb2-B-3 | KR232217 | *Albizia versicolor* | Mozambique | Teixeira and Rodríguez-Echeverría, 2016 |
| *B.* sp. Alb4-2-X | KR232233 | *Albizia versicolor* | Mozambique | Teixeira and Rodríguez-Echeverría, 2016 |
| *B.* sp. F85-4-2 | KR232252 | *Faidherbia albida* | Mozambique | Teixeira and Rodríguez-Echeverría, 2016 |
| *B.* sp. Alb1-C1 | KR232214 | *Albizia versicolor* | Mozambique | Teixeira and Rodríguez-Echeverría, 2016 |
| *B.* sp. Alb3-3-1 | KR232221 | *Albizia versicolor* | Mozambique | Teixeira and Rodríguez-Echeverría, 2016 |
| *B.* sp. F82-1-2 | KR232236 | *Faidherbia albida* | Mozambique | Teixeira and Rodríguez-Echeverría, 2016 |
| *B.* sp. F83-1-4 | KR232242 | *Faidherbia albida* | Mozambique | Teixeira and Rodríguez-Echeverría, 2016 |
| *B.* sp. F83-5-2 | KR232246 | *Faidherbia albida* | Mozambique | Teixeira and Rodríguez-Echeverría, 2016 |
| *B.* sp. F86-1-2 | KR232254 | *Faidherbia albida* | Mozambique | Teixeira and Rodríguez-Echeverría, 2016 |
| *B.* sp. F86-1-4 | KR232256 | *Faidherbia albida* | Mozambique | Teixeira and Rodríguez-Echeverría, 2016 |
| *B.* sp. F85-2-3 | Kr232249 | *Faidherbia albida* | Mozambique | Teixeira and Rodríguez-Echeverría, 2016 |
| *B.* sp. F83-2-1 | KR232243 | *Faidherbia albida* | Mozambique | Teixeira and Rodríguez-Echeverría, 2016 |
| *B.* sp. F83-2-3 | KR232244 | *Faidherbia albida* | Mozambique | Teixeira and Rodríguez-Echeverría, 2016 |
| *B.* sp. F82-4-1 | KR232238 | *Faidherbia albida* | Mozambique | Teixeira and Rodríguez-Echeverría, 2016 |
| *B.* sp. F82-1-1 | KR232235 | *Faidherbia albida* | Mozambique | Teixeira and Rodríguez-Echeverría, 2016 |
| *B.* sp. F82-4-3 | KR232239 | *Faidherbia albida* | Mozambique | Teixeira and Rodríguez-Echeverría, 2016 |
| *B.* sp. F83-5-1 | KR232245 | *Faidherbia albida* | Mozambique | Teixeira and Rodríguez-Echeverría, 2016 |
| *B.* sp. F83-1-3 | KR232241 | *Faidherbia albida* | Mozambique | Teixeira and Rodríguez-Echeverría, 2016 |
| *B.* sp. F86-5-2 | KR232260 | *Faidherbia albida* | Mozambique | Teixeira and Rodríguez-Echeverría, 2016 |
| *B.* sp. F86-4-2 | KR232259 | *Faidherbia albida* | Mozambique | Teixeira and Rodríguez-Echeverría, 2016 |
| *B.* sp. F85-3-4 | KR232251 | *Faidherbia albida* | Mozambique | Teixeira and Rodríguez-Echeverría, 2016 |
| *B.* sp. F85-1-4 | KR232247 | *Faidherbia albida* | Mozambique | Teixeira and Rodríguez-Echeverría, 2016 |
| *B.* sp. F86-2-3 | KR232257 | *Faidherbia albida* | Mozambique | Teixeira and Rodríguez-Echeverría, 2016 |
| *B.* sp. F85-4-3 | KR232253 | *Faidherbia albida* | Mozambique | Teixeira and Rodríguez-Echeverría, 2016 |
| *B.* sp. F86-3-3 | KR232258 | *Faidherbia albida* | Mozambique | Teixeira and Rodríguez-Echeverría, 2016 |
| *B.* sp. F85-2-1 | KR232248 | *Faidherbia albida* | Mozambique | Teixeira and Rodríguez-Echeverría, 2016 |
| *B.* sp. F85-3-3 | KR232250 | *Faidherbia albida* | Mozambique | Teixeira and Rodríguez-Echeverría, 2016 |
| *B.* sp. Alb4-1-4 | KR232230 | *Albizia versicolor* | Mozambique | Teixeira and Rodríguez-Echeverría, 2016 |
| *B.* sp. Alb4-1-5 | KR232231 | *Albizia versicolor* | Mozambique | Teixeira and Rodríguez-Echeverría, 2016 |
| *B.* sp. Alb4-1-6 | KR232232 | *Albizia versicolor* | Mozambique | Teixeira and Rodríguez-Echeverría, 2016 |
| *B.* sp. Alb2-B-4 | KR232218 | *Albizia versicolor* | Mozambique | Teixeira and Rodríguez-Echeverría, 2016 |
| *B.* sp. Alb2-B-2 | KR232216 | *Albizia versicolor* | Mozambique | Teixeira and Rodríguez-Echeverría, 2016 |
| *B.* sp. Alb1-C-2 | KR232215 | *Albizia versicolor* | Mozambique | Teixeira and Rodríguez-Echeverría, 2016 |
| *B.* sp. Alb5-2-6 | KR232234 | *Albizia versicolor* | Mozambique | Teixeira and Rodríguez-Echeverría, 2016 |
| *B.* sp. Alb4-1-3 | KR232229 | *Albizia versicolor* | Mozambique | Teixeira and Rodríguez-Echeverría, 2016 |
| *B.* sp. Alb1-B-2 | KR232213 | *Albizia versicolor* | Mozambique | Teixeira and Rodríguez-Echeverría, 2016 |
| *B.* sp. Alb3-4-5 | KR232227 | *Albizia versicolor* | Mozambique | Teixeira and Rodríguez-Echeverría, 2016 |
| *B.* sp. Alb4-1-2 | KR232228 | *Albizia versicolor* | Mozambique | Teixeira and Rodríguez-Echeverría, 2016 |
| *B.* sp. Alb3-1-5 | KR232219 | *Albizia versicolor* | Mozambique | Teixeira and Rodríguez-Echeverría, 2016 |
| *B.* sp. Alb3-3-3 | KR232222 | *Albizia versicolor* | Mozambique | Teixeira and Rodríguez-Echeverría, 2016 |
| *B.* sp. Alb3-3-4 | KR232223 | *Albizia versicolor* | Mozambique | Teixeira and Rodríguez-Echeverría, 2016 |
| *B.* sp. Alb3-4-1 | KR232224 | *Albizia versicolor* | Mozambique | Teixeira and Rodríguez-Echeverría, 2016 |
| *B.* sp. Alb3-2-1 | KR232220 | *Albizia versicolor* | Mozambique | Teixeira and Rodríguez-Echeverría, 2016 |
| *B.* sp. Alb3-4-4 | KR232226 | *Albizia versicolor* | Mozambique | Teixeira and Rodríguez-Echeverría, 2016 |
| *B.* sp. Alb3-4-3 |  | *Albizia versicolor* | Mozambique | Teixeira and Rodríguez-Echeverría, 2016 |
| *B.* sp. F83-1-1 | KR232240 | *Faidherbia albida* | Mozambique | Teixeira and Rodríguez-Echeverría, 2016 |

**References:**

Ardley, J.K., Reeve, W.G., O’Hara, G.W., Yates, R.J., Dilworth, M.J., Howieson, J.G. (2013) Nodule morphology, symbiotic specificity and association with unusual rhizobia are distinguishing features of the genus *Listia* within the southern African crotalarioid clade *Lotononis* s.l. Annals of Botany 112: 1-15

Beukes, C.W., Stępkowski, T., Venter, S.N., Cłapa, T., Phalane, F.L., le Roux, M.M., Steenkamp, E.T. (2016) Crotalarieae and Genisteae of the South African Great Escarpment are nodulated by novel *Bradyrhizobium* species with unique and diverse symbiotic loci. Mol. Phylogenet. Evol. 39: 266-274

Bourebaba, Y., Dúran, D., Boulila, F., Ahnia, H., Boulila, A., Temprano, F. Palacios, J.M., Imperial, J., Ruiz-Argüeso, T., Rey, L. (2016) Diversity of *Bradyrhizobium* strains nodulating *Lupinus micranthus* on both sides of the Western Mediterranean: Algeria and Spain. Syst. Appl. Microbiol. 39: 266-274

Chahboune, R., Carro, L., Peix, A., Barrijal, S., Velázquez, E., Bedmar, E.J. (2011) *Bradyrhizobium cytisi* sp. nov., isolated from effective nodules of *Cytisus villosus*. Int. J. Syst. Evol. Microbiol. 61: 2922-2927

Chahboune, R., Carro, L., Peix, A., Ramírez-Bahena, M-H., Barrijal, S., Velázquez, E., Bedmar, E.J. (2012) *Bradyrhizobium rifense* sp. nov., isolated from effective nodules of *Cytisus villosus* grown in the Moroccan Rif. Syst. Appl. Microbiol. 35: 302-305

Chang, Y.L., Wang, J.Y., Wang, E.T., Liu, H.C., Sui, X.H., Chen, W.X. (2011) *Bradyrhizobium lablabi* sp. nov., isolated from effective nodules of *Lablab* *purpureus* and *Arachis hypogaea*. 61: 2496-2502

Da Silva, K., De Meyer, S.E., Rouws, L.F.M., Farias, E.N.C., dos Santos, M.A.O., O’Hara, G., Ardley, J.K., Willems, A., Pitard, R.M., Zilli, J.E. (2014) *Bradyrhizobium ingae* sp. nov., isolated from effective nodules of *Inga laurina* grown in Cerrado soil. Int. J. Syst. Evol. Microbiol. 64: 3395-3401

Delamuta, J.R.M., Ribeiro, R.A., Ormeño-Orrillo, E., Melo, I.S., Martínez-Romero, E., Hungria, M. (2013) Polyphasic evidence supporting the reclassification of *Bradyrhizobium japonicum* Group Ia strains as *Bradyrhizobium diazoefficiens* sp. nov. Int. J. Syst. Environ. Microbiol. doi: 10.1099/ijs.0.049130-0

Delamuta, J.R.M., Ribeiro, R.A., Ormeño-Orrillo, E., Parma, M.M., Melo, I.S., Martínez-Romero, E., Hungria, M. (2015) *Bradyrhizobium tropiciagri* sp. nov. and *Bradyrhizobium embrapense* sp. nov., nitrogen-fixing symbionts of tropical forage legumes. Int. J. Syst. Evol. Microbiol. 65: 4424-4433

Durán, D., Rey, L., Mayo, J., Zúñiga-Dávila, D., Imperial, J., Ruiz-Argüeso, T., Martínez-Romero, E., Ormeño-Orrillo, E. (2014a) *Bradyrhizobium paxllaeri* sp. nov. and *Bradyrhizobium icense* sp. nov., nitrogen-fixing rhizobial symbionts of Lima bean (*Phaseolus lunatus* L.) in Peru. Int. J. Syst. Evol. Microbiol. 64: 2072-2078

Durán, D., Rey, L., Navarro, A., Busquets, A., Imperial, J., Ruiz-Argüeso, T. (2014b) *Bradyrhizobium valentinum* sp. nov., isolated from effective nodules of *Lupinus mariae-josephae*, a lupine endemic of basic-lime soils in Eastern Spain. Syst. Appl. Microbiol. 37: 336-341

Ferraz Helene, L.C., Delamuta, J.R.M., Ribeiro, R.A., Ormeño-Orrillo, E., Rogel, M.A., Martínez-Romero, E., Hungria, M. (2015) *Bradyrhizobium viridifuturi* sp. nov., encompassing nitrogen-fixing symbionts of legumes used for green manure and environmental services. Int. J. Syst. Evol. Microbiol. 65: 4441-4448

Grönemeyer, J.L., Chimwamurombe, P., Reinhold-Hurek, B. (2015b) *Bradyrhizobium* *subterraneum* sp. nov., a symbiotic nitrogen-fixing bacterium from root nodules of groundnuts. Int. J. Syst. Evol. Microbiol. 65: 3241-3247

Grönemeyer, J.L., Hurek, T., Reinhold-Hurek, B. (2015a) *Bradyrhizobium kavangense* sp. nov., a symbiotic nitrogen-fixing bacterium from root nodules of traditional Namibian pulses. Int. J. Syst. Evol. Microbiol. 65: 4886-4894

Grönemeyer, J.L., Hurek, T., Bünger, W., Reinhold-Hurek, B. (2016) *Bradyrhizobium vignae* sp. nov., a nitrogen-fixing symbiont isolated from effective nodules of *Vigna* and *Arachis*. Int. J. Syst. Evol. Microbiol. 66: 62-69

Grönemeyer, J.L., Kulkarni, A., Berkelmann, D., Hurek, T., Reinhold-Hurek, B. (2014) Rhizobia indigenous to the Okavango region in sub-Saharan Africa: diversity, adaptations, and host specificity. Appl. Environ. Microbiol. 80: 7244-7257

Guerrouj, K., Ruíz-Díez, B., Chahboune, R., Ramírez-Bahena, M-H., Abdelmoumen, H., Quiñones, M.A., El Idrissi, M.M., Velázquez, E., Fernández-Pascual, M., Bedmar, E.J., Peix, A. (2013) Definition of a novel symbiovar (sv. Retamae) within *Bradyrhizobium retamae* sp. nov., nodulating *Retama sphaerocarpa* and *Retama monosperma*. Syst. Appl. Microbiol. 36: 218-223

Islam, M.S., Kawasaki, H., Muramatsu, Y., Nakagawa, Y., Seki, T. (2008) *Bradyrhizobium iriomotense* sp. nov., isolated from a tumor-like root of the legume *Entada koshunensis* from Iriomote island in Japan. Biosci. Biotechnol. Biochem. 72: 1416-1429

Jarabo-Lorenzo, A., Pérez-Galdona, R., Donate-Correa, J., Rivas, R., Velázquez, E., Hernández, M., Temprano, F., Martínez-Molina, E., Ruiz-Argüeso, T., León-Barrios, M. (2003) Genetic diversity of bradyrhizobial populations from diverse geographic origins that nodulate *Lupinus* spp. and *Ornithopus* spp. Syst. Appl. Microbiol. 26: 611-623

Jordan, D.C. (1982) Transfer or *Rhizobium japonicum* Buchanan 1980 to *Bradyrhizobium* gen. nov., a genus of slow-growing, root nodule bacteria from leguminous plants. Int. J. Syst. Bacteriol. 32: 136-139

Kuykendall, L.D., Saxena, B., Devine, T.E., Udell, S.E. (1992) Genetic diversity in *Bradyrhizobium japonicum* Jordan 1982 and a proposal for *Bradyrhizobium* *elkanii* sp. nov. Canadian Journal of Microbiology 38: 501-505

Lemaire, B., Dlodlo, O., Chimphango, S., Stirton, C., Schrire, B., Boatwright, J.S., Honnay, O., Smets, E., Sprent, J., James, E.K., Muasya, A.M. (2015) Symbiotic diversity, specificity and distribution of rhizobia in native legumes of the Core Cape Subregion (South Africa). FEMS Microbiology Ecology 91. doi: 10.1093/femsec/fiu024

Li, Y.H., Wang, R., Zhang, X.X., Young, J.P.W., Wang, E.T., Sui, X.H., Chen, W.X. (2015) *Bradyrhizobium guangdongense* sp. nov. and *Bradyrhizobium* *guangxiense* sp. nov., isolated from effective nodules of peanut. Int. J. Syst. Evol. Microbiol. 65: 4655-4661

Lu, J.K., Dou, Y.J., Zhu, Y.J., Wang, S.K., Sui, X.H., Kang, L.H. (2014) *Bradyrhizobium ganzhouense* sp. nov., an effective symbiotic bacterium isolated from *Acacia* *melanoxylon* R. Br. nodules. Int. J. Syst. Evol. Microbiol. 64: 1900-1905

Martins da Costa, E., Azarias Guimarães, A., Vicentin, R.P., de Almeida Ribeiro, P.R., Ribas Leão, A.C., Balsanelli, E., Lebbe, L., Aerts, M., Willems, A., de Souza Moreira, F.M. (2017) *Bradyrhizobium brasilense* sp. nov., a symbiotic nitrogen-fixing bacterium isolated from Brazilian tropical soils. Arch. Microbiol. doi: 10.1007/s00203-017-1390-1

Michel, D.C., Passos, S.R., Simões-Araujo, J.L., Baraúna, A.C., da Silva, K., Parma, M.M., Melo, I.S., De Meyer, S.E., O’Hara, G., Zilli, J.E. (2017) *Bradyrhizobium centrolobii* and *Bradyrhizobium macuxiense* sp. nov. isolated from Centrolobium paraense grown in soil of Amazonia, Brazil. Arch. Microbiol. doi: 10.1007/s00203-017-1340-y

Msaddak, A., Durán, D., Rejili, M., Mars, M., Ruiz-Argüeso, T., Imperial, J., Palacios, J., Rey, L. (2017) Diverse bacteria affiliated with the genera *Microvirga*, *Phyllobacterium* and *Bradyrhizobium* nodulate *Lupinus micranthus* growing in soils of Northern Tunisia. Appl. Environ. Microbiol. doi: 10.1128/AEM.02820-16

Peix, A., Ramírez-Bahena, M.H., Flores-Félix, J.D., de la Vega, P.A., Rivas, R., Mateos, P.F., Igual, J.M., Martínez-Molina, E., Trujillo, M.E., Velázquez, E. (2015) Revision of the taxonomic status of the species *Rhizobium lupini* and reclassification as *Bradyrhizobium lupini* comb. nov. Int. J. Syst. Evol. Microbiol. 65: 1213-1219

Ramírez-Bahena, M-H., Chachboune, R., Peix, A., Velázquez, E. (2013) Reclassification of *Agromonas oligotrophica* into the genus *Bradyrhizobium* as *Bradyrhizobium oligotrophicum* comb. nov. Int. J. Syst. Evol. Microbiol. 63: 1013-1016

Ramírez-Bahena, M.H., Flores-Félix, J.D., Chahboune, R., Toro, M., Velázquez, E., Peix, A. (2016) *Bradyrhizobium centrosemae* (symbiovar centrosemae) sp. nov., *Bradyrhizobium americanum* (symbiovar phaseolarum) sp. nov. and a new symbiovar (tropici) of *Bradyrhizobium viridifuturi* establish symbiosis with *Centrosema* species native to America. Syst. Appl. Microbiol. 39: 378-383

Ramírez-Bahena, M.H., Peix, A., Rivas, R., Camacho, M., Rodríguez-Navarro, D.N., Mateos, P.F., Martínez-Molina, E., Willems, A., Velázquez, E. (2009) *Bradyrhizobium pachyrhizi* sp. nov. and *Bradyrhizobium jicamae* sp. nov., isolated from effective nodules of *Pachyrhizus erosus*. Int. J. Syst. Evol. Microbiol. 59: 1929-1934

Rivas, R., Willems, A., Palomo, J.L., García-Benavides, P., Mateos, P.F., Martínez-Molina, E., Gillis, M., Velázquez, E. (2004) *Bradyrhizobium betae* sp. nov., isolated from roots of Beta vulgaris affected by tumor-like deformations. Int. J. Syst. Evol. Microbiol. 54: 1271-1275

Silva, F.V., De Meyer, S.E., Simões-Araújo, J.L., da Costa Barbé, T., Xavier, G.R., O’Hara, G., Ardley, J.K., Rumjanek, N.G., Willems, A., Zilli, J.E. (2014) *Bradyrhizobium manausense* sp. nov., isolated from effective nodules of *Vigna unguiculata* grown in Brazilian Amazonian rainforest soils. Int. J. Syst. Evol. Microbiol. 64: 2358-2363

Steenkamp, E.T., Stępkowski, T., Przymusiak, A., Botha, W.J., Law, I.J. (2008) Cowpea and peanut in southern Africa are nodulated by diverse *Bradyrhizobium* strains harbouring nodution genes that belong to the large pantropical clade common in Africa. Mol. Phylogenet. Evol. 48: 1131-1144

Stępkowski, T., Hughes, C.E., Law, I.J., Markiewicz, Ł., Gurda, D., Chlebicka, A., Moulin, L. (2007) Diversification of Lupine *Bradyrhizobium* strains: evidence from nodulation gene trees. Appl. Environ. Microbiol. 73: 3254-3264

Teixeira, H., Rodríguez-Echeverría, S. (2016) Identification of symbiotic nitrogen-fixing bacteria from three African leguminous trees in Gorongosa National Park. Syst. Appl. Microbiol. Doi: http://dx.doi.org/10.1016/j.syapm.2016.05.004

Van Berkum, P., Leibold, J.M., Eardly, B.D. (2006) Proposal for combining *Bradyrhizobium* spp. (*Aeschynomene indica*) with *Blastobacter denitrificans* and to transfer *Blastobacter denitrificans* (Hirsch and Muller, 1985) to the genus *Bradyrhizobium* as *Bradyrhizobium denitrificans* (comb. nov.). Syst. Appl. Microbiol. 29: 207-215

Vinuesa, P., León-Barrios, M., Silva, C., Willems, A., Jarabo-Lorenzo, A., Pérez-Galdona, R., Werner, D., Martínez-Romero, E. (2005) *Bradyrhizobium* *canariense* sp. nov., an acid-tolerant endosymbiont that nodulates endemic genistoid legumes (Papilionoideae: Genisteae) from the Canary Islands, along with *Bradyrhizobium japonicum* bv. *genistearum*, *Bradyrhizobium* genospecies alpha and *Bradyrhizobium* genospecies beta. Int. J. Syst. Evol. Microbiol. 55: 569-575

Wang, R., Chang, Y.L., Zheng, W.T., Zhang, D., Zhang, X.X., Sui, X.H., Wang, E.T., Hu, J.Q., Zhang, L.Y., Chen, W.X. (2013) *Bradyrhizobium arachidis* sp. nov., isolated from effective nodules of *Arachis hypogaea* grown in China. Syst. Appl. Microbiol. 36: 101-105

Wang, J.Y., Wang, R., Zhang, Y.M., Liu, H.C., Chen, W.F., Wang, E.T., Sui, X.H., Chen, W.X. (2012) *Bradyrhizobium daqingense* sp. nov., isolated from nodules of soybean grown in Daqing City of China. Int. J. Syst. Evol. Microbiol. doi: 10.1099/ijs.0.034280-0

Xu, L.M., Ge, C., Cui, Z., Li, J., Fan, H. (1995) *Bradyrhizobium liaoningense* sp. nov., isolated from the root nodules of soybeans. Int. J. Syst. Bacteriol. 45: 706-711

Yao, Y., Sui, X.H., Zhang, X.X., Wang, E.T., Chen, W.X. (2015) *Bradyrhizobium erythrophlei* sp. nov. and *Bradyrhizobium ferriligni* sp. nov., isolated from effective nodules of *Erythrophleum fordii*. Int. J. Syst. Evol. Microbiol. 65: 1831-1837

Yao, Z.Y., Kan, F.L., Wang, E.T., Wei, G.H., Chen, W.X. (2002) Characterization of rhizobia that nodulate legumes species of the genus *Lespedeza* and description of *Bradyrhizobium yuanmingense* sp. nov. Int. J. Syst. Evol. Microbiol. 52: 2219-2230

Yu, X., Cloutier, S., Tambong, J.T., Bromfield, E.S.P. (2014) *Bradyrhizobium ottawaense* sp. nov., a symbiotic nitrogen fixing bacterium from root nodules of soybeans in Canada. Int. J. Syst. Evol. Microbiol. 64: 3202-3207

Zhang, Y.M., Li, Y.Jr., Chen, W.F., Wang, E.T., Sui, X.H., Li, Q.Q., Zhang, Y.Z., Zhou, Y.G., Chen, W.X. (2012) *Bradyrhizobium huanghuaihaiense* sp. nov., an effective symbiotic bacterium isolated from soybean (Glycine max L.) nodules. Int. J. Syst. Evol. Microbiol. 62: 1951-1957

Zilli, J.E., Baraúna, A.C., da Silva, K., De Meyer, S.E., Farias, E.N.C., Kaminski, P.E., da Costa, I.B., Ardley, J.K., Willems, A., Camacho, N.N., dos S. Dourado, F., O’Hara, G. (2014) *Bradyrhizobium neotropicale* sp. nov., isolated from effective nodules of *Centrolobium paraense*. Int. J. Syst. Evol. Microbiol. 64: 3950-3957
