# Supplementary material for: Both Alpha- and Beta-Rhizobia Occupy the Root Nodules of Vachellia karroo in South Africa
Source: Front Microbiol. 2019 Jun 4;10:1195. doi: 10.3389/fmicb.2019.01195 (PMC6558075; doi:10.3389/fmicb.2019.01195)
Supplement: Supplementary file 6 [file Table_6.DOCX]

Supplementary Table S6. Information regarding the phylogenetic analyses for each *recA* dataset

|  | ***Ensifer*** | ***Mesorhizobium*** | ***Paraburkholderia*** | ***Rhizobium*** | ***Bradyrhizobium*** |
| --- | --- | --- | --- | --- | --- |
| Number of isolates | 32 | 27 | 19 | 6 | 1 |
| ***recA*** |  |  |  |  |  |
| Initial number of taxa | 160 | 174 | 213 | 132 | 272 |
| Number of haplotypes | 94 | 131 | 156 | 115 | 194 |
| Length of alignment | 525 | 474 | 549 | 480 | 492 |
| Evolutionary model^a^ | TrN+I+G | TrN+I+G | TIM2+I+G | HKY+I+G | TIM2+I+G |
|  |  |  |  |  |  |

^a^ Specifics for the evolutionary models used: TrN (Tamura and Nei, 1993); TIM2 (“transitional model”; Posada, 2008) and HKY (Hasegawa et al. 1985). For each of these models the datasets had a proportion of invariable sites (+I) and had rate variation among sites (+G).
