# Supplementary material for: Both Alpha- and Beta-Rhizobia Occupy the Root Nodules of Vachellia karroo in South Africa
Source: Front Microbiol. 2019 Jun 4;10:1195. doi: 10.3389/fmicb.2019.01195 (PMC6558075; doi:10.3389/fmicb.2019.01195)
Supplement: Supplementary file 7 [file Data_Sheet_1.PDF]

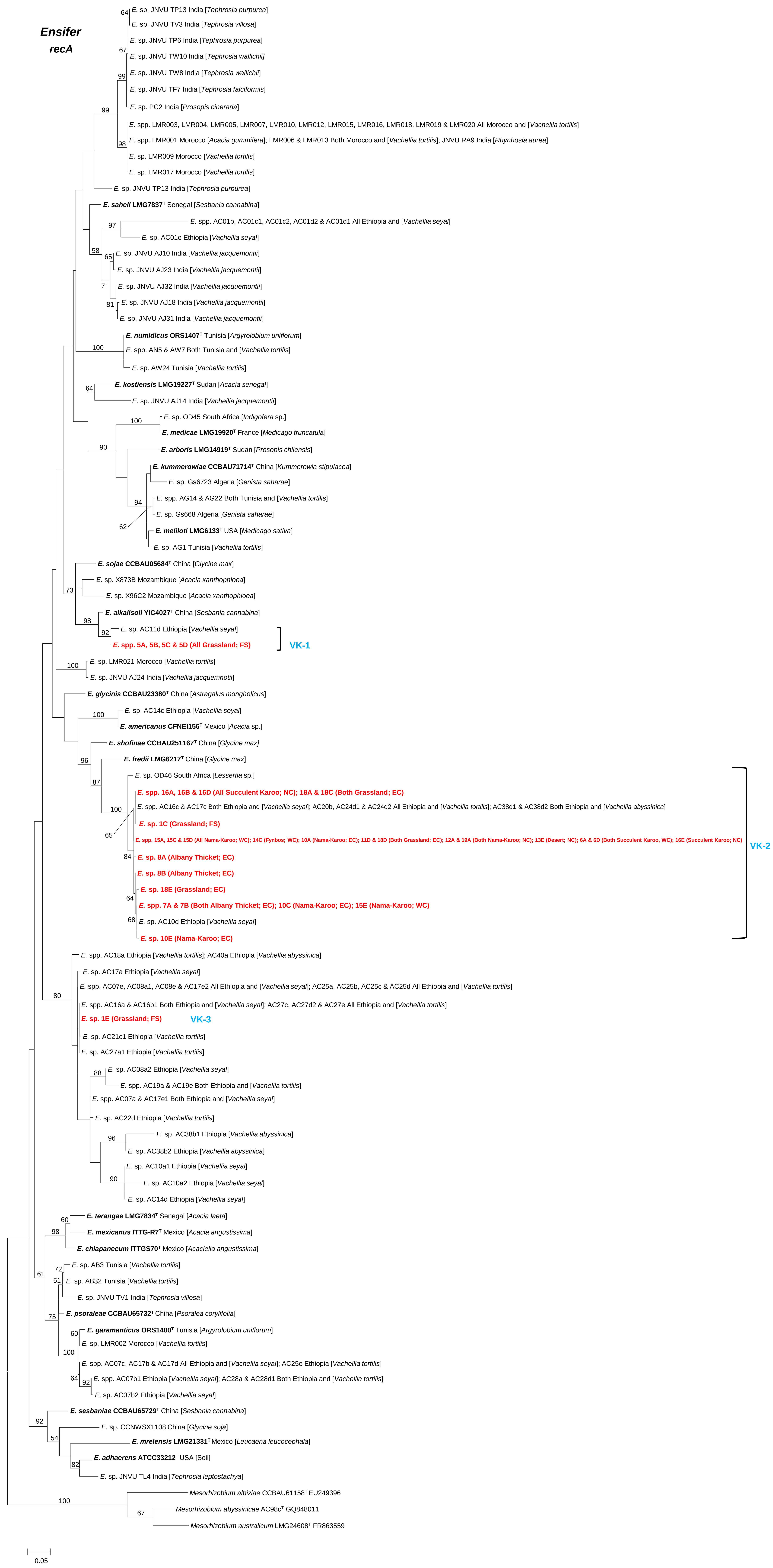
Ensifer
recA
E. sp. JNVU TP13 India [Tephrosia purpurea]
E. sp. JNVU TV3 India [Tephrosia villosa]
E. sp. JNVU TP6 India [Tephrosia purpurea]
E. sp. JNVU TW10 India [Tephrosia wallichii]
E. sp. JNVU TW8 India [Tephrosia wallichii]
E. sp. JNVU TF7 India [Tephrosia falciformis]
E. sp. PC2 India [Prosopis cineraria]
E. spp. LMR003, LMR004, LMR005, LMR007, LMR010, LMR012, LMR015, LMR016, LMR018, LMR019 & LMR020 All Morocco and [Vachellia tortilis]
E. spp. LMR001 Morocco [Acacia gummifera]; LMR006 & LMR013 Both Morocco and [Vachellia tortilis]; JNVU RA9 India [Rhynhosia aurea]
E. sp. LMR009 Morocco [Vachellia tortilis]
E. sp. LMR017 Morocco [Vachellia tortilis]
E. sp. JNVU TP13 India [Tephrosia purpurea]
E. saheli LMG7837T Senegal [Sesbania cannabina]
E. spp. AC01b, AC01c1, AC01c2, AC01d2 & AC01d1 All Ethiopia and [Vachellia seyal]
E. sp. AC01e Ethiopia [Vachellia seyal]
E. sp. JNVU AJ10 India [Vachellia jacquemontii]
E. sp. JNVU AJ23 India [Vachellia jacquemontii]
E. sp. JNVU AJ32 India [Vachellia jacquemontii]
E. sp. JNVU AJ18 India [Vachellia jacquemontii]
E. sp. JNVU AJ31 India [Vachellia jacquemontii]
E. numidicus ORS1407T Tunisia [Argyrolobium uniflorum]
E. spp. AN5 & AW7 Both Tunisia and [Vachellia tortilis]
E. sp. AW24 Tunisia [Vachellia tortilis]
E. kostiensis LMG19227T Sudan [Acacia senegal]
E. sp. JNVU AJ14 India [Vachellia jacquemontii]
E. sp. OD45 South Africa [Indigofera sp.]
E. medicae LMG19920T France [Medicago truncatula]
E. arboris LMG14919T Sudan [Prosopis chilensis]
E. kummerowiae CCBAU71714T China [Kummerowia stipulacea]
E. sp. Gs6723 Algeria [Genista saharae]
E. spp. AG14 & AG22 Both Tunisia and [Vachellia tortilis]
E. sp. Gs668 Algeria [Genista saharae]
E. meliloti LMG6133T USA [Medicago sativa]
E. sp. AG1 Tunisia [Vachellia tortilis]
E. sojae CCBAU05684T China [Glycine max]
E. sp. X873B Mozambique [Acacia xanthophloea]
E. sp. X96C2 Mozambique [Acacia xanthophloea]
E. alkalisoli YIC4027T China [Sesbania cannabina]
E. sp. AC11d Ethiopia [Vachellia seyal]
E. spp. 5A, 5B, 5C & 5D (All Grassland; FS)
VK-1
E. sp. LMR021 Morocco [Vachellia tortilis]
E. sp. JNVU AJ24 India [Vachellia jacquemnotii]
E. glycinis CCBAU23380T China [Astragalus mongholicus]
E. sp. AC14c Ethiopia [Vachellia seyal]
E. americanus CFNEI156T Mexico [Acacia sp.]
E. shofinae CCBAU251167T China [Glycine max]
E. fredii LMG6217T China [Glycine max]
E. sp. OD46 South Africa [Lessertia sp.]
E. spp. 16A, 16B & 16D (All Succulent Karoo; NC); 18A & 18C (Both Grassland; EC)
E. spp. AC16c & AC17c Both Ethiopia and [Vachellia seyal]; AC20b, AC24d1 & AC24d2 All Ethiopia and [Vachellia tortilis]; AC38d1 & AC38d2 Both Ethiopia and [Vachellia abyssinica]
E. sp. 1C (Grassland; FS)
E. spp. 15A, 15C & 15D (All Nama-Karoo; WC); 14C (Fynbos; WC); 10A (Nama-Karoo; EC); 11D & 18D (Both Grassland; EC); 12A & 19A (Both Nama-Karoo; NC); 13E (Desert; NC); 6A & 6D (Both Succulent Karoo, WC); 16E (Succulent Karoo; NC)
E. sp. 8A (Albany Thicket; EC)
E. sp. 8B (Albany Thicket; EC)
E. sp. 18E (Grassland; EC)
E. spp. 7A & 7B (Both Albany Thicket; EC); 10C (Nama-Karoo; EC); 15E (Nama-Karoo; WC)
E. sp. AC10d Ethiopia [Vachellia seyal]
E. sp. 10E (Nama-Karoo; EC)
VK-2
E. spp. AC18a Ethiopia [Vachellia tortilis]; AC40a Ethiopia [Vachellia abyssinica]
E. sp. AC17a Ethiopia [Vachellia seyal]
E. spp. AC07e, AC08a1, AC08e & AC17e2 All Ethiopia and [Vachellia seyal]; AC25a, AC25b, AC25c & AC25d All Ethiopia and [Vachellia tortilis]
E. spp. AC16a & AC16b1 Both Ethiopia and [Vachellia seyal]; AC27c, AC27d2 & AC27e All Ethiopia and [Vachellia tortilis]
E. sp. 1E (Grassland; FS)
VK-3
E. sp. AC21c1 Ethiopia [Vachellia tortilis]
E. sp. AC27a1 Ethiopia [Vachellia tortilis]
E. sp. AC08a2 Ethiopia [Vachellia seyal]
E. spp. AC19a & AC19e Both Ethiopia and [Vachellia tortilis]
E. spp. AC07a & AC17e1 Both Ethiopia and [Vachellia seyal]
E. sp. AC22d Ethiopia [Vachellia tortilis]
E. sp. AC38b1 Ethiopia [Vachellia abyssinica]
E. sp. AC38b2 Ethiopia [Vachellia abyssinica]
E. sp. AC10a1 Ethiopia [Vachellia seyal]
E. sp. AC10a2 Ethiopia [Vachellia seyal]
E. sp. AC14d Ethiopia [Vachellia seyal]
E. terangae LMG7834T Senegal [Acacia laeta]
E. mexicanus ITTG-R7T Mexico [Acacia angustissima]
E. chiapanecum ITTGS70T Mexico [Acaciella angustissima]
E. sp. AB3 Tunisia [Vachellia tortilis]
E. sp. AB32 Tunisia [Vachellia tortilis]
E. sp. JNVU TV1 India [Tephrosia villosa]
E. psoraleae CCBAU65732T China [Psoralea corylifolia]
E. garamanticus ORS1400T Tunisia [Argyrolobium uniflorum]
E. sp. LMR002 Morocco [Vachellia tortilis]
E. spp. AC07c, AC17b & AC17d All Ethiopia and [Vachellia seyal]; AC25e Ethiopia [Vachellia tortilis]
E. spp. AC07b1 Ethiopia [Vachellia seyal]; AC28a & AC28d1 Both Ethiopia and [Vachellia tortilis]
E. sp. AC07b2 Ethiopia [Vachellia seyal]
E. sesbaniae CCBAU65729T China [Sesbania cannabina]
E. sp. CCNWSX1108 China [Glycine soja]
E. mrelensis LMG21331T Mexico [Leucaena leucocephala]
E. adhaerens ATCC33212T USA [Soil]
E. sp. JNVU TL4 India [Tephrosia leptostachya]
Mesorhizobium albiziae CCBAU61158T EU249396
Mesorhizobium abyssinicae AC98cT GQ848011
Mesorhizobium australicum LMG24608T FR863559
0.05

Suppl. Fig. S1 A *recA* maximum-likelihood phylogeny of the genus *Ensifer*. Isolates from this study appear in red followed by information for the biome and province (abbreviated as described for Table 1) from which the soil of the 'trapping' experiment originates. Lineages pertaining to this study (VK-1 to VK-3) are demarcated and indicated in blue. *Ensifer* type strains appear in bold and all the isolates have their country of origin and source or host listed. GenBank accession numbers and references for all the isolates in the ingroup are listed in Suppl. Table S1. Three *Mesorhizobium* species are used as the outgroup and are indicated with the type strain numbers and GenBank accession numbers. Bootstrap support of ≥ 50% are indicated and scale bar indicates the number of nucleotide substitutions per site.
