# Supplementary material for: Both Alpha- and Beta-Rhizobia Occupy the Root Nodules of Vachellia karroo in South Africa
Source: Front Microbiol. 2019 Jun 4;10:1195. doi: 10.3389/fmicb.2019.01195 (PMC6558075; doi:10.3389/fmicb.2019.01195)
Supplement: Supplementary file 8 [file Data_Sheet_2.PDF]

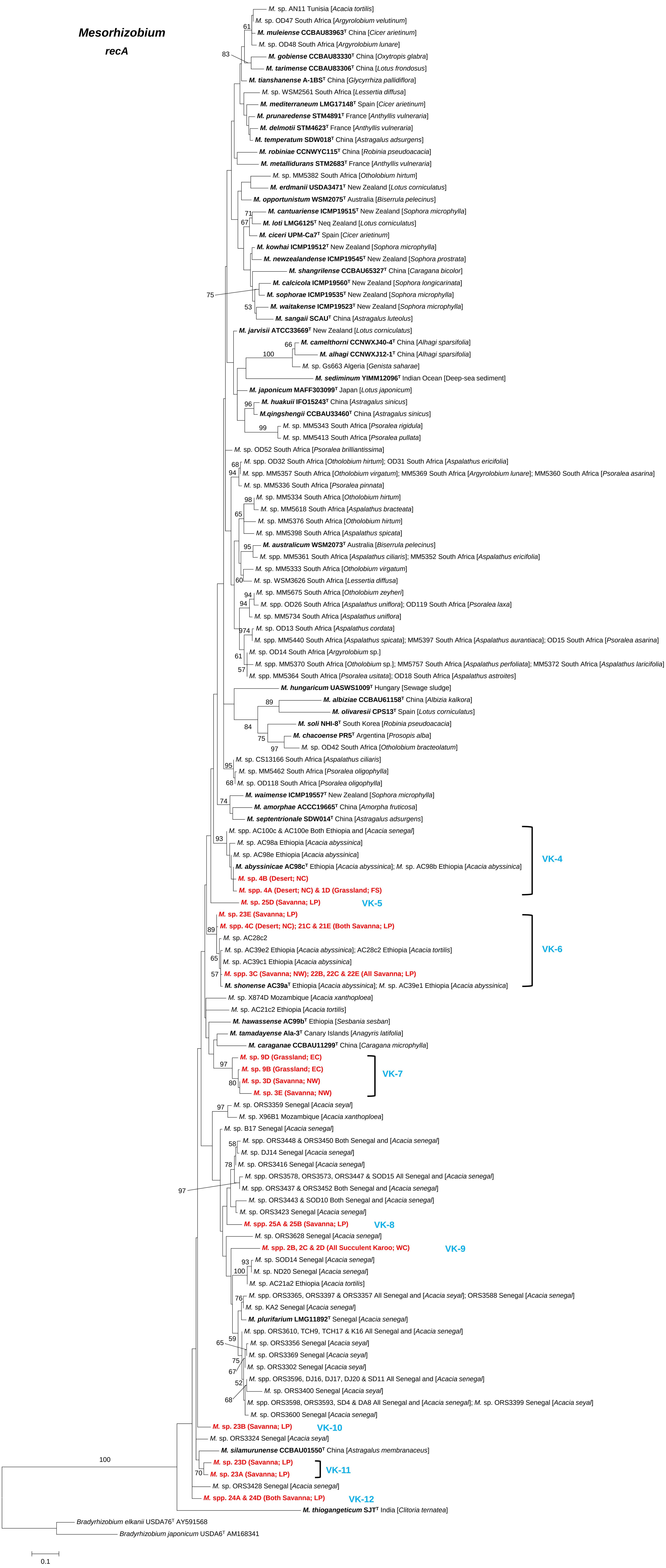

Mesorhizobium
recA
M. sp. AN11 Tunisia [Acacia tortilis]
M. sp. OD47 South Africa [Argyrolobium velutinum]
M. muleiense CCBAU83963T China [Cicer arietinum]
M. sp. OD48 South Africa [Argyrolobium lunare]
M. gobiense CCBAU83330T China [Oxytropis glabra]
M. tarimense CCBAU83306T China [Lotus frondosus]
M. tianshanense A-1BST China [Glycyrrhiza pallidiflora]
M. sp. WSM2561 South Africa [Lessertia diffusa]
M. mediterraneum LMG17148T Spain [Cicer arietinum]
M. prunaredense STM4891T France [Anthyllis vulneraria]
M. delmotii STM4623T France [Anthyllis vulneraria]
M. temperatum SDW018T China [Astragalus adsurgens]
M. robiniae CCNWYC115T China [Robinia pseudoacacia]
M. metallidurans STM2683T France [Anthyllis vulneraria]
M. sp. MM5382 South Africa [Otholobium hirtum]
M. erdmanii USDA3471T New Zealand [Lotus corniculatus]
M. opportunistum WSM2075T Australia [Biserrula pelecinus]
M. cantuariense ICMP19515T New Zealand [Sophora microphylla]
M. loti LMG6125T Neq Zealand [Lotus corniculatus]
M. ciceri UPM-Ca7T Spain [Cicer arietinum]
M. kowhai ICMP19512T New Zealand [Sophora microphylla]
M. newzealandense ICMP19545T New Zealand [Sophora prostrata]
M. shangrilense CCBAU65327T China [Caragana bicolor]
M. calcicola ICMP19560T New Zealand [Sophora longicarinata]
M. sophorae ICMP19535T New Zealand [Sophora microphylla]
M. waitakense ICMP19523T New Zealand [Sophora microphylla]
M. sangaii SCAUT China [Astragalus luteolus]
M. jarvisii ATCC33669T New Zealand [Lotus corniculatus]
M. camelthorni CCNWXJ40-4T China [Alhagi sparsifolia]
M. alhagi CCNWXJ12-1T China [Alhagi sparsifolia]
M. sp. Gs663 Algeria [Genista saharae]
M. sediminum YIMM12096T Indian Ocean [Deep-sea sediment]
M. japonicum MAFF303099T Japan [Lotus japonicum]
M. huakuii IFO15243T China [Astragalus sinicus]
M.qingshengii CCBAU33460T China [Astragalus sinicus]
M. sp. MM5343 South Africa [Psoralea rigidula]
M. sp. MM5413 South Africa [Psoralea pullata]
M. sp. OD52 South Africa [Psoralea brilliantissima]
M. spp. OD32 South Africa [Otholobium hirtum]; OD31 South Africa [Aspalathus ericifolia]
M. spp. MM5357 South Africa [Otholobium virgatum]; MM5369 South Africa [Argyrolobium lunare]; MM5360 South Africa [Psoralea asarina]
M. sp. MM5336 South Africa [Psoralea pinnata]
M. sp. MM5334 South Africa [Otholobium hirtum]
M. sp. MM5618 South Africa [Aspalathus bracteata]
M. sp. MM5376 South Africa [Otholobium hirtum]
M. sp. MM5398 South Africa [Aspalathus spicata]
M. australicum WSM2073T Australia [Biserrula pelecinus]
M. spp. MM5361 South Africa [Aspalathus ciliaris]; MM5352 South Africa [Aspalathus ericifolia]
M. sp. MM5333 South Africa [Otholobium virgatum]
M. sp. WSM3626 South Africa [Lessertia diffusa]
M. sp. MM5675 South Africa [Otholobium zeyheri]
M. sp. OD26 South Africa [Aspalathus uniflora]; OD119 South Africa [Psoralea laxa]
M. sp. MM5734 South Africa [Aspalathus uniflora]
M. sp. OD13 South Africa [Aspalathus cordata]
M. spp. MM5440 South Africa [Aspalathus spicata]; MM5397 South Africa [Aspalathus aurantiaca]; OD15 South Africa [Psoralea asarina]
M. sp. OD14 South Africa [Argyrolobium sp.]
M. spp. MM5370 South Africa [Otholobium sp.]; MM5757 South Africa [Aspalathus perfoliata]; MM5372 South Africa [Aspalathus laricifolia]
M. spp. MM5364 South Africa [Psoralea usitata]; OD18 South Africa [Aspalathus astroites]
M. hungaricum UASWS1009T Hungary [Sewage sludge]
M. albiziae CCBAU61158T China [Albizia kalkora]
M. olivaresii CPS13T Spain [Lotus corniculatus]
M. soli NHI-8T South Korea [Robinia pseudoacacia]
M. chacoense PR5T Argentina [Prosopis alba]
M. sp. OD42 South Africa [Otholobium bracteolatum]
M. sp. CS13166 South Africa [Aspalathus ciliaris]
M. sp. MM5462 South Africa [Psoralea oligophylla]
M. sp. OD118 South Africa [Psoralea oligophylla]
M. waimense ICMP19557T New Zealand [Sophora microphylla]
M. amorphae ACCC19665T China [Amorpha fruticosa]
M. septentrionale SDW014T China [Astragalus adsurgens]
M. spp. AC100c & AC100e Both Ethiopia and [Acacia senegal]
M. sp. AC98a Ethiopia [Acacia abyssinica]
M. sp. AC98e Ethiopia [Acacia abyssinica]
M. abyssinicae AC98cT Ethiopia [Acacia abyssinica]; M. sp. AC98b Ethiopia [Acacia abyssinica]
M. sp. 4B (Desert; NC)
M. spp. 4A (Desert; NC) & 1D (Grassland; FS)
VK-4
M. sp. 25D (Savanna; LP)
VK-5
M. sp. 23E (Savanna; LP)
M. spp. 4C (Desert; NC); 21C & 21E (Both Savanna; LP)
M. sp. AC28c2
M. sp. AC39e2 Ethiopia [Acacia abyssinica]; AC28c2 Ethiopia [Acacia tortilis]
M. sp. AC39c1 Ethiopia [Acacia abyssinica]
M. spp. 3C (Savanna; NW); 22B, 22C & 22E (All Savanna; LP)
M. shonense AC39aT Ethiopia [Acacia abyssinica]; M. sp. AC39e1 Ethiopia [Acacia abyssinica]
VK-6
M. sp. X874D Mozambique [Acacia xanthoploea]
M. sp. AC21c2 Ethiopia [Acacia tortilis]
M. hawassense AC99bT Ethiopia [Sesbania sesban]
M. tamadayense Ala-3T Canary Islands [Anagyris latifolia]
M. caraganae CCBAU11299T China [Caragana microphylla]
M. sp. 9D (Grassland; EC)
M. sp. 9B (Grassland; EC)
M. sp. 3D (Savanna; NW)
M. sp. 3E (Savanna; NW)
VK-7
M. sp. ORS3359 Senegal [Acacia seyal]
M. sp. X96B1 Mozambique [Acacia xanthoploea]
M. sp. B17 Senegal [Acacia senegal]
M. spp. ORS3448 & ORS3450 Both Senegal and [Acacia senegal]
M. sp. DJ14 Senegal [Acacia senegal]
M. sp. ORS3416 Senegal [Acacia senegal]
M. spp. ORS3578, ORS3573, ORS3447 & SOD15 All Senegal and [Acacia senegal]
M. spp. ORS3437 & ORS3452 Both Senegal and [Acacia senegal]
M. sp. ORS3443 & SOD10 Both Senegal and [Acacia senegal]
M. sp. ORS3423 Senegal [Acacia senegal]
M. spp. 25A & 25B (Savanna; LP)
VK-8
M. sp. ORS3628 Senegal [Acacia senegal]
M. spp. 2B, 2C & 2D (All Succulent Karoo; WC)
VK-9
M. sp. SOD14 Senegal [Acacia senegal]
M. sp. ND20 Senegal [Acacia senegal]
M. sp. AC21a2 Ethiopia [Acacia tortilis]
M. spp. ORS3365, ORS3397 & ORS3357 All Senegal and [Acacia seyal]; ORS3588 Senegal [Acacia senegal]
M. sp. KA2 Senegal [Acacia senegal]
M. plurifarium LMG11892T Senegal [Acacia senegal]
M. spp. ORS3610, TCH9, TCH17 & K16 All Senegal and [Acacia senegal]
M. sp. ORS3356 Senegal [Acacia seyal]
M. sp. ORS3369 Senegal [Acacia seyal]
M. sp. ORS3302 Senegal [Acacia seyal]
M. spp. ORS3596, DJ16, DJ17, DJ20 & SD11 All Senegal and [Acacia senegal]
M. sp. ORS3400 Senegal [Acacia seyal]
M. spp. ORS3598, ORS3593, SD4 & DA8 All Senegal and [Acacia senegal]; M. sp. ORS3399 Senegal [Acacia seyal]
M. sp. ORS3600 Senegal [Acacia senegal]
M. sp. 23B (Savanna; LP)
VK-10
M. sp. ORS3324 Senegal [Acacia seyal]
M. silamurunense CCBAU01550T China [Astragalus membranaceus]
M. sp. 23D (Savanna; LP)
M. sp. 23A (Savanna; LP)
VK-11
M. sp. ORS3428 Senegal [Acacia senegal]
M. spp. 24A & 24D (Both Savanna; LP)
VK-12
M. thiogangeticum SJTT India [Clitoria ternatea]
Bradyrhizobium elkanii USDA76T AY591568
Bradyrhizobium japonicum USDA6T AM168341
0.1

Suppl. Fig. S2 A *recA* maximum-likelihood phylogeny of the genus *Mesorhizobium*. Isolates from this study are indicated in red, followed by information for biome and province (abbreviated as for Table 1) from which the 'trapping' soil was collected. Demarcated lineages (VK-4 to VK-12) are indicated in blue. All *Mesorhizobium* type strains are in bold and all isolates appear with information regarding their country of origin and source or host. GenBank accession numbers and associated references for all the isolates in the phylogeny are listed in Suppl. Table S2. *Bradyrhizobium elkanii* and *B. japonicum* were used as the outgroup and their accession numbers are listed on the phylogeny. Bootstrap support of ≥ 50% are indicated and the scale bar indicates the number of nucleotide substitutions per site.
