# Supplementary material for: Both Alpha- and Beta-Rhizobia Occupy the Root Nodules of Vachellia karroo in South Africa
Source: Front Microbiol. 2019 Jun 4;10:1195. doi: 10.3389/fmicb.2019.01195 (PMC6558075; doi:10.3389/fmicb.2019.01195)
Supplement: Supplementary file 9 [file Data_Sheet_3.PDF]

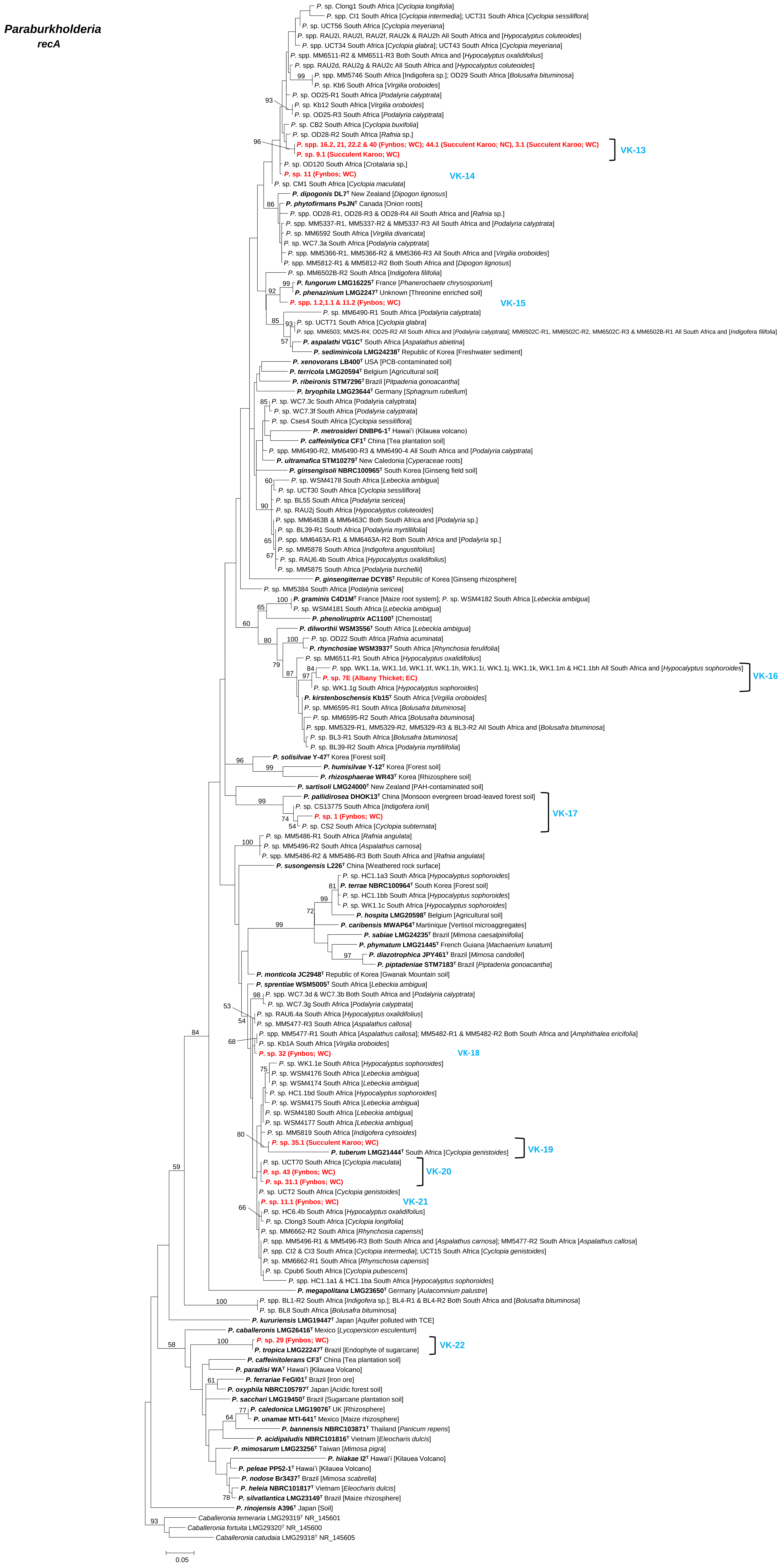

Paraburkholderia
recA
P. sp. Clong1 South Africa [Cyclopia longifolia]
P. spp. CI1 South Africa [Cyclopia intermedia]; UCT31 South Africa [Cyclopia sessiliflora]
P. sp. UCT56 South Africa [Cyclopia meyeriana]
P. spp. RAU2i, RAU2l, RAU2f, RAU2k & RAU2h All South Africa and [Hypocalyptus coluteoides]
P. spp. UCT34 South Africa [Cyclopia glabra]; UCT43 South Africa [Cyclopia meyeriana]
P. spp. MM6511-R2 & MM6511-R3 Both South Africa and [Hypocalyptus oxalidifolius]
P. spp. RAU2d, RAU2g & RAU2c All South Africa and [Hypocalyptus coluteoides]
P. spp. MM5746 South Africa [Indigofera sp.]; OD29 South Africa [Bolusafra bituminosa]
P. sp. Kb6 South Africa [Virgilia oroboides]
P. sp. OD25-R1 South Africa [Podalyria calyptrata]
P. sp. Kb12 South Africa [Virgilia oroboides]
P. sp. OD25-R3 South Africa [Podalyria calyptrata]
P. sp. CB2 South Africa [Cyclopia buxifolia]
P. sp. OD28-R2 South Africa [Rafnia sp.]
P. spp. 16.2, 21, 22.2 & 40 (Fynbos; WC); 44.1 (Succulent Karoo; NC), 3.1 (Succulent Karoo; WC)
P. sp. 9.1 (Succulent Karoo; WC)
VK-13
P. sp. OD120 South Africa [Crotalaria sp.]
P. sp. 11 (Fynbos; WC)
VK-14
P. sp. CM1 South Africa [Cyclopia maculata]
P. dipogonis DL7T New Zealand [Dipogon lignosus]
P. phytofirmans PsJNT Canada [Onion roots]
P. spp. OD28-R1, OD28-R3 & OD28-R4 All South Africa and [Rafnia sp.]
P. spp. MM5337-R1, MM5337-R2 & MM5337-R3 All South Africa and [Podalyria calyptrata]
P. sp. MM6592 South Africa [Virgilia divaricata]
P. sp. WC7.3a South Africa [Podalyria calyptrata]
P. spp. MM5366-R1, MM5366-R2 & MM5366-R3 All South Africa and [Virgilia oroboides]
P. spp. MM5812-R1 & MM5812-R2 Both South Africa and [Dipogon lignosus]
P. sp. MM6502B-R2 South Africa [Indigofera filifolia]
P. fungorum LMG16225T France [Phanerochaete chrysosporium]
P. phenazinium LMG2247T Unknown [Threonine enriched soil]
P. spp. 1.2,1.1 & 11.2 (Fynbos; WC)
VK-15
P. sp. MM6490-R1 South Africa [Podalyria calyptrata]
P. sp. UCT71 South Africa [Cyclopia glabra]
P. spp. MM6503; MM25-R4; OD25-R2 All South Africa and [Podalyria calyptrata]; MM6502C-R1, MM6502C-R2, MM6502C-R3 & MM6502B-R1 All South Africa and [Indigofera filifolia]
P. aspalathi VG1CT South Africa [Aspalathus abietina]
P. sediminicola LMG24238T Republic of Korea [Freshwater sediment]
P. xenovorans LB400T USA [PCB-contaminated soil]
P. terricola LMG20594T Belgium [Agricultural soil]
P. ribeironis STM7296T Brazil [Pitpadenia gonoacantha]
P. bryophila LMG23644T Germany [Sphagnum rubellum]
P. sp. WC7.3c South Africa [Podalyria calyptrata]
P. sp. WC7.3f South Africa [Podalyria calyptrata]
P. sp. Cses4 South Africa [Cyclopia sessiliflora]
P. metrosideri DNBP6-1T Hawai'i (Kilauea volcano)
P. caffeinilytica CF1T China [Tea plantation soil]
P. spp. MM6490-R2, MM6490-R3 & MM6490-4 All South Africa and [Podalyria calyptrata]
P. ultramafica STM10279T New Caledonia [Cyperaceae roots]
P. ginsengisoli NBRC100965T South Korea [Ginseng field soil]
P. sp. WSM4178 South Africa [Lebeckia ambigua]
P. sp. UCT30 South Africa [Cyclopia sessiliflora]
P. sp. BL55 South Africa [Podalyria sericea]
P. sp. RAU2j South Africa [Hypocalyptus coluteoides]
P. spp. MM6463B & MM6463C Both South Africa and [Podalyria sp.]
P. sp. BL39-R1 South Africa [Podalyria myrtillifolia]
P. spp. MM6463A-R1 & MM6463A-R2 Both South Africa and [Podalyria sp.]
P. sp. MM5878 South Africa [Indigofera angustifolius]
P. sp. RAU6.4b South Africa [Hypocalyptus oxalidifolius]
P. sp. MM5875 South Africa [Podalyria burchellii]
P. ginsengiterrae DCY85T Republic of Korea [Ginseng rhizosphere]
P. sp. MM5384 South Africa [Podalyria sericea]
P. graminis C4D1MT France [Maize root system]; P. sp. WSM4182 South Africa [Lebeckia ambigua]
P. sp. WSM4181 South Africa [Lebeckia ambigua]
P. phenoliruptrix AC1100T [Chemostat]
P. dilworthii WSM3556T South Africa [Lebeckia ambigua]
P. sp. OD22 South Africa [Rafnia acuminata]
P. rhynchosiae WSM3937T South Africa [Rhynchosia ferulifolia]
P. sp. MM6511-R1 South Africa [Hypocalyptus oxalidifolius]
P. spp. WK1.1a, WK1.1d, WK1.1f, WK1.1h, WK1.1i, WK1.1j, WK1.1k, WK1.1m & HC1.1bh All South Africa and [Hypocalyptus sophoroides]
P. sp. 7E (Albany Thicket; EC)
VK-16
P. sp. WK1.1g South Africa [Hypocalyptus sophoroides]
P. kirstenboschensis Kb15T South Africa [Virgilia oroboides]
P. sp. MM6595-R1 South Africa [Bolusafra bituminosa]
P. sp. MM6595-R2 South Africa [Bolusafra bituminosa]
P. spp. MM5329-R1, MM5329-R2, MM5329-R3 & BL3-R2 All South Africa and [Bolusafra bituminosa]
P. sp. BL3-R1 South Africa [Bolusafra bituminosa]
P. sp. BL39-R2 South Africa [Podalyria myrtillifolia]
P. solisilvae Y-47T Korea [Forest soil]
P. humisilvae Y-12T Korea [Forest soil]
P. rhizosphaerae WR43T Korea [Rhizosphere soil]
P. sartisoli LMG24000T New Zealand [PAH-contaminated soil]
P. pallidirosea DHOK13T China [Monsoon evergreen broad-leaved forest soil]
P. sp. CS13775 South Africa [Indigofera ionii]
P. sp. 1 (Fynbos; WC)
VK-17
P. sp. CS2 South Africa [Cyclopia subternata]
P. sp. MM5486-R1 South Africa [Rafnia angulata]
P. sp. MM5496-R2 South Africa [Aspalathus carnosa]
P. spp. MM5486-R2 & MM5486-R3 Both South Africa and [Rafnia angulata]
P. susongensis L226T China [Weathered rock surface]
P. sp. HC1.1a3 South Africa [Hypocalyptus sophoroides]
P. terrae NBRC100964T South Korea [Forest soil]
P. sp. HC1.1bb South Africa [Hypocalyptus sophoroides]
P. sp. WK1.1c South Africa [Hypocalyptus sophoroides]
P. hospita LMG20598T Belgium [Agricultural soil]
P. caribensis MWAP64T Martinique [Vertisol microaggregates]
P. sabiae LMG24235T Brazil [Mimosa caesalpiniifolia]
P. phymatum LMG21445T French Guiana [Machaerium lunatum]
P. diazotrophica JPY461T Brazil [Mimosa candollei]
P. piptadeniae STM7183T Brazil [Piptadenia gonoacantha]
P. monticola JC2948T Republic of Korea [Gwanak Mountain soil]
P. sprentiae WSM5005T South Africa [Lebeckia ambigua]
P. spp. WC7.3d & WC7.3b Both South Africa and [Podalyria calyptrata]
P. sp. WC7.3g South Africa [Podalyria calyptrata]
P. sp. RAU6.4a South Africa [Hypocalyptus oxalidifolius]
P. sp. MM5477-R3 South Africa [Aspalathus callosa]
P. spp. MM5477-R1 South Africa [Aspalathus callosa]; MM5482-R1 & MM5482-R2 Both South Africa and [Amphithalea ericifolia]
P. sp. Kb1A South Africa [Virgilia oroboides]
P. sp. 32 (Fynbos; WC)
VK-18
P. sp. WK1.1e South Africa [Hypocalyptus sophoroides]
P. sp. WSM4176 South Africa [Lebeckia ambigua]
P. sp. WSM4174 South Africa [Lebeckia ambigua]
P. sp. HC1.1bd South Africa [Hypocalyptus sophoroides]
P. sp. WSM4175 South Africa [Lebeckia ambigua]
P. sp. WSM4180 South Africa [Lebeckia ambigua]
P. sp. WSM4177 South Africa [Lebeckia ambigua]
P. sp. MM5819 South Africa [Indigofera cytisoides]
P. sp. 35.1 (Succulent Karoo; WC)
P. tuberum LMG21444T South Africa [Cyclopia genistoides]
VK-19
P. sp. UCT70 South Africa [Cyclopia maculata]
P. sp. 43 (Fynbos; WC)
P. sp. 31.1 (Fynbos; WC)
VK-20
P. sp. UCT2 South Africa [Cyclopia genistoides]
P. sp. 11.1 (Fynbos; WC)
VK-21
P. sp. HC6.4b South Africa [Hypocalyptus oxalidifolius]
P. sp. Clong3 South Africa [Cyclopia longifolia]
P. sp. MM6662-R2 South Africa [Rhynchosia capensis]
P. spp. MM5496-R1 & MM5496-R3 Both South Africa and [Aspalathus carnosa]; MM5477-R2 South Africa [Aspalathus callosa]
P. spp. CI2 & CI3 South Africa [Cyclopia intermedia]; UCT15 South Africa [Cyclopia genistoides]
P. sp. MM6662-R1 South Africa [Rhynschosia capensis]
P. sp. Cpub6 South Africa [Cyclopia pubescens]
P. spp. HC1.1a1 & HC1.1ba South Africa [Hypocalyptus sophoroides]
P. megapolitana LMG23650T Germany [Aulacomnium palustre]
P. spp. BL1-R2 South Africa [Indigofera sp.]; BL4-R1 & BL4-R2 Both South Africa and [Bolusafra bituminosa]
P. sp. BL8 South Africa [Bolusafra bituminosa]
P. kururiensis LMG19447T Japan [Aquifer polluted with TCE]
P. caballeronis LMG26416T Mexico [Lycopersicon esculentum]
P. sp. 29 (Fynbos; WC)
P. tropica LMG22247T Brazil [Endophyte of sugarcane]
VK-22
P. caffeinitolerans CF3T China [Tea plantation soil]
P. paradisi WAT Hawai'i [Kilauea Volcano]
P. ferrariae FeGl01T Brazil [Iron ore]
P. oxyphila NBRC105797T Japan [Acidic forest soil]
P. sacchari LMG19450T Brazil [Sugarcane plantation soil]
P. caledonica LMG19076T UK [Rhizosphere]
P. unamae MTI-641T Mexico [Maize rhizosphere]
P. bannensis NBRC103871T Thailand [Panicum repens]
P. acidipaludis NBRC101816T Vietnam [Eleocharis dulcis]
P. mimosarum LMG23256T Taiwan [Mimosa pigra]
P. hiiakae I2T Hawai'i [Kilauea Volcano]
P. peleae PP52-1T Hawai'i [Kilauea Volcano]
P. nodosa Br3437T Brazil [Mimosa scabrella]
P. heleia NBRC101817T Vietnam [Eleocharis dulcis]
P. silvatlantica LMG23149T Brazil [Maize rhizosphere]
P. rinojensis A396T Japan [Soil]
Caballeronia temeraria LMG29319T NR_145601
Caballeronia fortuita LMG29320T NR_145600
Caballeronia catudaia LMG29318T NR_145605
0.05

Suppl. Fig. S3 A *recA* maximum-likelihood phylogeny of the genus *Paraburkholderia*. Isolates from this study appear in red followed by information for the biome and province (as abbreviated for Table 1) from which the 'trapping' soil originated. *Paraburkholderia* type strains appear in bold, and all the isolates list information for their country of origin and host or source. Suppl. Table S3 contains the GenBank accessions and associated references for all the *Paraburkholderia* isolates included in this phylogeny. The delineated *Vachellia karroo*-associated lineages (VK-13 to VK-22) are listed in blue. Three species of the genus *Caballeronia* were used for an outgroup (their accession numbers are listed in the phylogeny). Nodes with ≥ 50% bootstrap support are shown, while the scale bar indicates the number of nucleotide substitutions per site.
