# Supplementary material for: Both Alpha- and Beta-Rhizobia Occupy the Root Nodules of Vachellia karroo in South Africa
Source: Front Microbiol. 2019 Jun 4;10:1195. doi: 10.3389/fmicb.2019.01195 (PMC6558075; doi:10.3389/fmicb.2019.01195)
Supplement: Supplementary file 10 [file Data_Sheet_4.PDF]

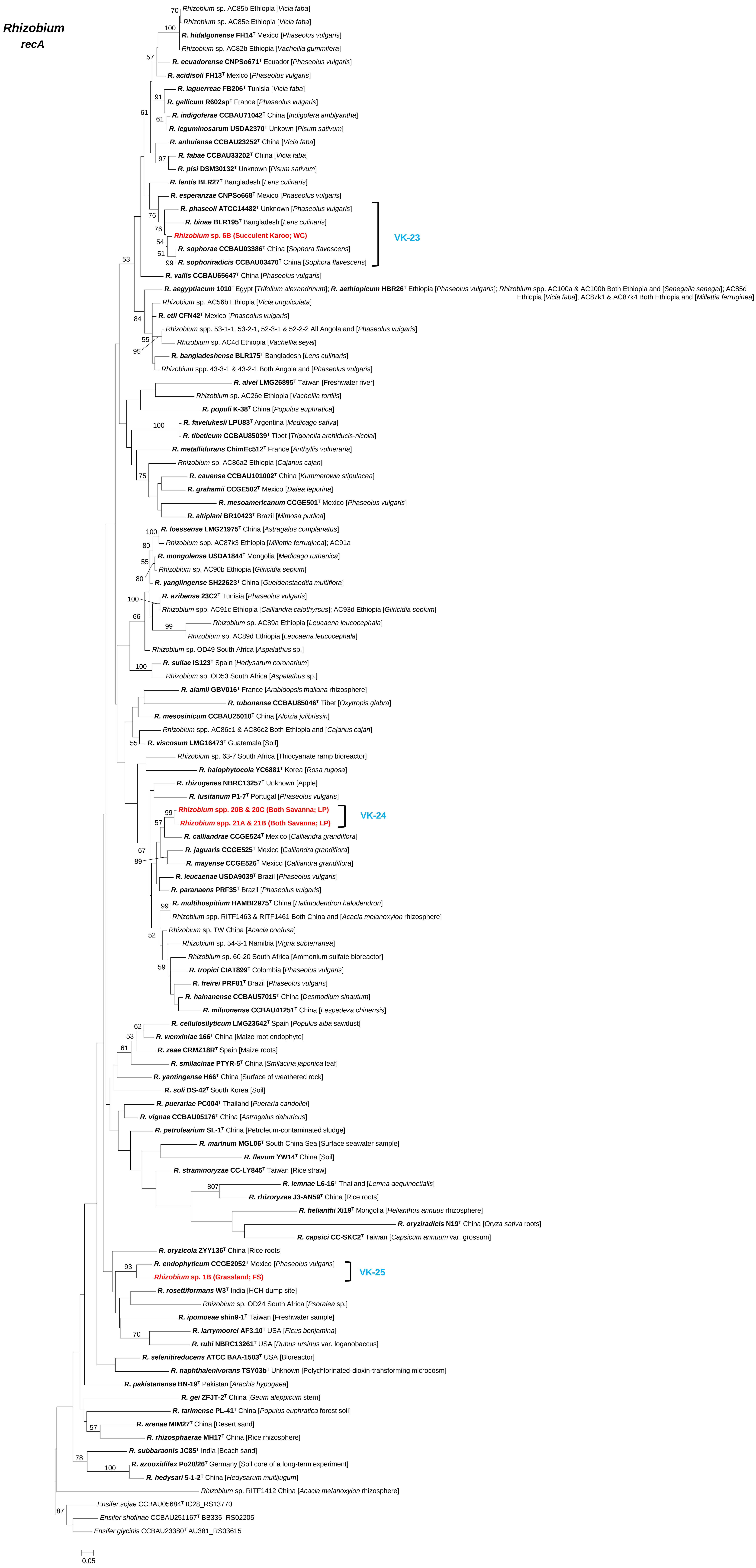

Rhizobium recA
Rhizobium sp. AC85b Ethiopia [Vicia faba]
Rhizobium sp. AC85e Ethiopia [Vicia faba]
R. hidalgonense FH14^{T} Mexico [Phaseolus vulgaris]
Rhizobium sp. AC82b Ethiopia [Vachellia gummifera]
R. ecuadorense CNPSo671^{T} Ecuador [Phaseolus vulgaris]
R. acidisoli FH13^{T} Mexico [Phaseolus vulgaris]
R. laguerreae FB206^{T} Tunisia [Vicia faba]
R. gallicum R602sp^{T} France [Phaseolus vulgaris]
R. indigoferae CCBAU71042^{T} China [Indigofera amblyantha]
R. leguminosarum USDA2370^{T} Unkown [Pisum sativum]
R. anhuiense CCBAU23252^{T} China [Vicia faba]
R. fabae CCBAU33202^{T} China [Vicia faba]
R. pisi DSM30132^{T} Unknown [Pisum sativum]
R. lentis BLR27^{T} Bangladesh [Lens culinaris]
R. esperanzae CNPSo668^{T} Mexico [Phaseolus vulgaris]
R. phaseoli ATCC14482^{T} Unknown [Phaseolus vulgaris]
R. binae BLR195^{T} Bangladesh [Lens culinaris]
Rhizobium sp. 6B (Succulent Karoo; WC)
R. sophorae CCBAU03386^{T} China [Sophora flavescens]
R. sophoriradicis CCBAU03470^{T} China [Sophora flavescens]
VK-23
R. vallis CCBAU65647^{T} China [Phaseolus vulgaris]
R. aegyptiacum 1010^{T} Egypt [Trifolium alexandrinum]; R. aethiopicum HBR26^{T} Ethiopia [Phaseolus vulgaris]; Rhizobium spp. AC100a & AC100b Both Ethiopia and [Senegalia senegal]; AC85d Ethiopia [Vicia faba]; AC87k1 & AC87k4 Both Ethiopia and [Millettia ferruginea]
Rhizobium sp. AC56b Ethiopia [Vicia unguiculata]
R. etli CFN42^{T} Mexico [Phaseolus vulgaris]
Rhizobium spp. 53-1-1, 53-2-1, 52-3-1 & 52-2-2 All Angola and [Phaseolus vulgaris]
Rhizobium sp. AC4d Ethiopia [Vachellia seyal]
R. bangladeshense BLR175^{T} Bangladesh [Lens culinaris]
Rhizobium spp. 43-3-1 & 43-2-1 Both Angola and [Phaseolus vulgaris]
R. alvei LMG26895^{T} Taiwan [Freshwater river]
Rhizobium sp. AC26e Ethiopia [Vachellia tortilis]
R. populi K-38^{T} China [Populus euphratica]
R. favelukesii LPU83^{T} Argentina [Medicago sativa]
R. tibeticum CCBAU85039^{T} Tibet [Trigonella archiducis-nicolai]
R. metallidurans ChimEc512^{T} France [Anthyllis vulneraria]
Rhizobium sp. AC86a2 Ethiopia [Cajanus cajan]
R. cauense CCBAU101002^{T} China [Kummerowia stipulacea]
R. grahamii CCGE502^{T} Mexico [Dalea leporina]
R. mesoamericanum CCGE501^{T} Mexico [Phaseolus vulgaris]
R. altiplani BR10423^{T} Brazil [Mimosa pudica]
R. loessense LMG21975^{T} China [Astragalus complanatus]
Rhizobium spp. AC87k3 Ethiopia [Millettia ferruginea]; AC91a
R. mongolense USDA1844^{T} Mongolia [Medicago ruthenica]
Rhizobium sp. AC90b Ethiopia [Gliricidia sepium]
R. yanglingense SH22623^{T} China [Gueldenstaedtia multiflora]
R. azibense 23C2^{T} Tunisia [Phaseolus vulgaris]
Rhizobium spp. AC91c Ethiopia [Calliandra calothyrsus]; AC93d Ethiopia [Gliricidia sepium]
Rhizobium sp. AC89a Ethiopia [Leucaena leucocephala]
Rhizobium sp. AC89d Ethiopia [Leucaena leucocephala]
Rhizobium sp. OD49 South Africa [Aspalathus sp.]
R. sullae IS123^{T} Spain [Hedysarum coronarium]
Rhizobium sp. OD53 South Africa [Aspalathus sp.]
R. alamii GBV016^{T} France [Arabidopsis thaliana rhizosphere]
R. tubonense CCBAU85046^{T} Tibet [Oxytropis glabra]
R. mesosinicum CCBAU25010^{T} China [Albizia julibrissin]
Rhizobium spp. AC86c1 & AC86c2 Both Ethiopia and [Cajanus cajan]
R. viscosum LMG16473^{T} Guatemala [Soil]
Rhizobium sp. 63-7 South Africa [Thiocyanate ramp bioreactor]
R. halophytocola YC6881^{T} Korea [Rosa rugosa]
R. rhizogenes NBRC13257^{T} Unknown [Apple]
R. lusitanum P1-7^{T} Portugal [Phaseolus vulgaris]
Rhizobium spp. 20B & 20C (Both Savanna; LP)
Rhizobium spp. 21A & 21B (Both Savanna; LP)
VK-24
R. calliandrae CCGE524^{T} Mexico [Calliandra grandiflora]
R. jaguaris CCGE525^{T} Mexico [Calliandra grandiflora]
R. mayense CCGE526^{T} Mexico [Calliandra grandiflora]
R. leucaenae USDA9039^{T} Brazil [Phaseolus vulgaris]
R. paranaens PRF35^{T} Brazil [Phaseolus vulgaris]
R. multihospitium HAMBI2975^{T} China [Halimodendron halodendron]
Rhizobium spp. RITF1463 & RITF1461 Both China and [Acacia melanoxylon rhizosphere]
Rhizobium sp. TW China [Acacia confusa]
Rhizobium sp. 54-3-1 Namibia [Vigna subterranea]
Rhizobium sp. 60-20 South Africa [Ammonium sulfate bioreactor]
R. tropici CIAT899^{T} Colombia [Phaseolus vulgaris]
R. freirei PRF81^{T} Brazil [Phaseolus vulgaris]
R. hainanense CCBAU57015^{T} China [Desmodium sinautum]
R. miluonense CCBAU41251^{T} China [Lespedeza chinensis]
R. cellulosilyticum LMG23642^{T} Spain [Populus alba sawdust]
R. wenxiniae 166^{T} China [Maize root endophyte]
R. zeae CRMZ18R^{T} Spain [Maize roots]
R. smilacinae PTYR-5^{T} China [Smilacina japonica leaf]
R. yantingense H66^{T} China [Surface of weathered rock]
R. soli DS-42^{T} South Korea [Soil]
R. puerariae PC004^{T} Thailand [Pueraria candollei]
R. vignae CCBAU05176^{T} China [Astragalus dahuricus]
R. petrolearium SL-1^{T} China [Petroleum-contaminated sludge]
R. marinum MGL06^{T} South China Sea [Surface seawater sample]
R. flavum YW14^{T} China [Soil]
R. straminoryzae CC-LY845^{T} Taiwan [Rice straw]
R. lemnae L6-16^{T} Thailand [Lemna aequinoctialis]
R. rhizoryzae J3-AN59^{T} China [Rice roots]
R. helianthi Xi19^{T} Mongolia [Helianthus annuus rhizosphere]
R. oryziradicis N19^{T} China [Oryza sativa roots]
R. capsici CC-SKC2^{T} Taiwan [Capsicum annuum var. grossum]
R. oryzicola ZYY136^{T} China [Rice roots]
R. endophyticum CCGE2052^{T} Mexico [Phaseolus vulgaris]
Rhizobium sp. 1B (Grassland; FS)
VK-25
R. rosettiformans W3^{T} India [HCH dump site]
Rhizobium sp. OD24 South Africa [Psoralea sp.]
R. ipomoeae shin9-1^{T} Taiwan [Freshwater sample]
R. larrymoorei AF3.10^{T} USA [Ficus benjamina]
R. rubi NBRC13261^{T} USA [Rubus ursinus var. loganobaccus]
R. selenitireducens ATCC BAA-1503^{T} USA [Bioreactor]
R. naphthalenivorans TSY03b^{T} Unknown [Polychlorinated-dioxin-transforming microcosm]
R. pakistanense BN-19^{T} Pakistan [Arachis hypogaea]
R. gei ZFJT-2^{T} China [Geum aleppicum stem]
R. tarimense PL-41^{T} China [Populus euphratica forest soil]
R. arenae MIM27^{T} China [Desert sand]
R. rhizosphaerae MH17^{T} China [Rice rhizosphere]
R. subbaraonis JC85^{T} India [Beach sand]
R. azooxidifex Po20/26^{T} Germany [Soil core of a long-term experiment]
R. hedysari 5-1-2^{T} China [Hedysarum multijugum]
Rhizobium sp. RITF1412 China [Acacia melanoxylon rhizosphere]
Ensifer sojae CCBAU05684^{T} IC28_RS13770
Ensifer shofinae CCBAU251167^{T} BB335_RS02205
Ensifer glycinis CCBAU23380^{T} AU381_RS03615
0.05

Suppl. Fig. S4 A *recA* maximum-likelihood phylogeny consisting of isolates of the genus *Rhizobium*. All type strains are indicated in bold, and all isolates list information for their country of origin and host or source. Isolates from this study appear in red together with the information for the biome and province (abbreviated as in Table 1) from which the 'trapping' soil originated. The lineages to which these isolates were assigned (VK-23 to VK-25) are in blue. GenBank accession numbers and the references associated with the included isolates can be found in Suppl. Table S4. Three *Ensifer* species were used as the outgroup for the analyses on which bootstrap support of ≥ 50% are indicated. The scale bar indicates the number of nucleotide substitutions per site.
