# Supplementary material for: Both Alpha- and Beta-Rhizobia Occupy the Root Nodules of Vachellia karroo in South Africa
Source: Front Microbiol. 2019 Jun 4;10:1195. doi: 10.3389/fmicb.2019.01195 (PMC6558075; doi:10.3389/fmicb.2019.01195)
Supplement: Supplementary file 11 [file Data_Sheet_5.PDF]

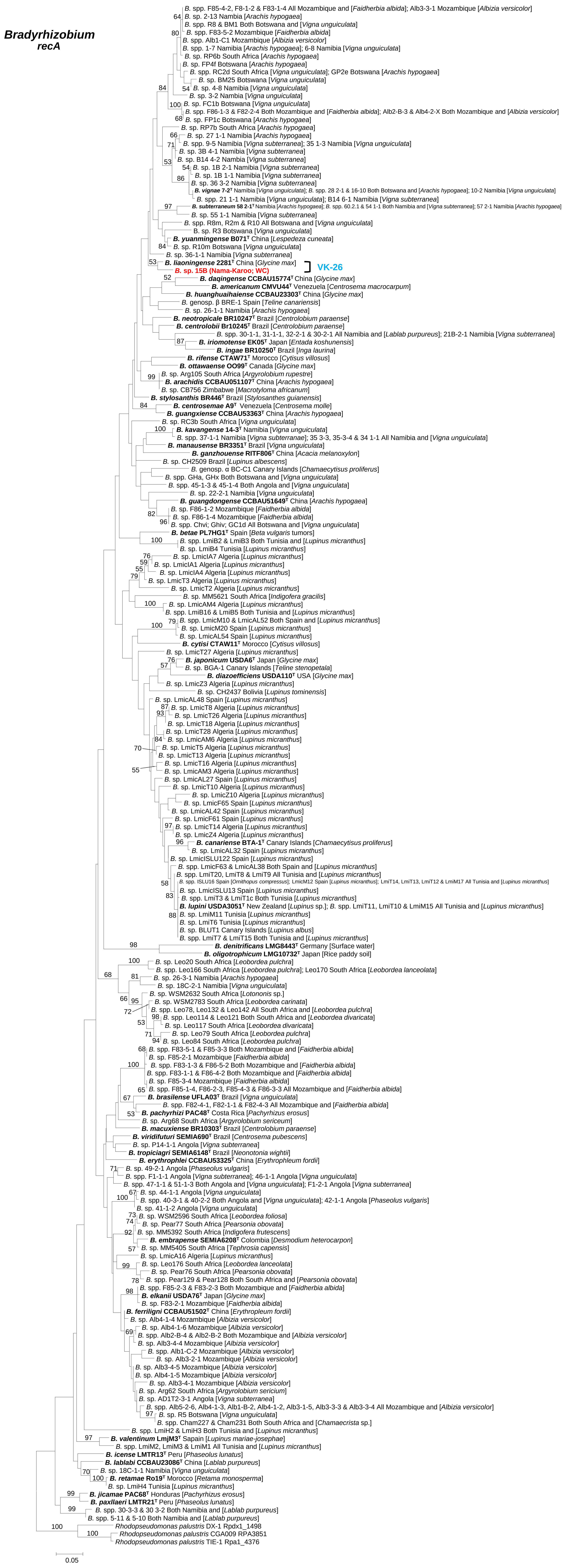

Bradyrhizobium
recA
B. spp. F85-4-2, F8-1-2 & F83-1-4 All Mozambique and [Faidherbia albida]; Alb3-3-1 Mozambique [Albizia versicolor]
B. sp. 2-13 Nambia [Arachis hypogaea]
B. spp. R8 & BM1 Both Botswana and [Vigna unguiculata]
B. sp. F83-5-2 Mozambique [Faidherbia albida]
B. spp. Alb1-C1 Mozambique [Albizia versicolor]
B. spp. 1-7 Namibia [Arachis hypogaea]; 6-8 Namibia [Vigna unguiculata]
B. sp. RP6b South Africa [Arachis hypogaea]
B. sp. FP4f Botswana [Arachis hypogaea]
B. spp. RC2d South Africa [Vigna unguiculata]; GP2e Botswana [Arachis hypogaea]
B. sp. BM25 Botswana [Vigna unguiculata]
B. sp. 4-8 Namibia [Vigna unguiculata]
B. sp. 3-2 Nambia [Vigna unguiculata]
B. sp. FC1b Botswana [Vigna unguiculata]
B. spp. F86-1-3 & F82-2-4 Both Mozambique and [Faidherbia albida]; Alb2-B-3 & Alb4-2-X Both Mozambique and [Albizia versicolor]
B. sp. FP1c Botswana [Arachis hypogaea]
B. sp. RP7b South Africa [Arachis hypogaea]
B. sp. 27 1-1 Namibia [Arachis hypogaea]
B. spp. 9-5 Namibia [Vigna subterranea]; 35 1-3 Namibia [Vigna unguiculata]
B. sp. 3B 4-1 Namibia [Vigna subterranea]
B. sp. B14 4-2 Nambia [Vigna subterranea]
B. sp. 1B 2-1 Namibia [Vigna subterranea]
B. sp. 1B 1-1 Namibia [Vigna subterranea]
B. sp. 36 3-2 Namibia [Vigna subterranea]
B. vignae 7-2T Namibia [Vigna unguiculata]; B. spp. 28 2-1 & 16-10 Both Botswana and [Arachis hypogaea]; 10-2 Namibia [Vigna unguiculata]
B. spp. 21 1-1 Namibia [Vigna unguiculata]; B14 6-1 Namibia [Vigna subterranea]
B. subterraneum 58 2-1T Namibia [Arachis hypogaea]; B. spp. 60.2.1 & 54 1-1 Both Namibia and [Vigna subterranea]; 57 2-1 Namibia [Arachis hypogaea]
B. sp. 55 1-1 Namibia [Vigna subterranea]
B. spp. R8m, R2m & R10 All Botswana and [Vigna unguiculata]
B. sp. R3 Botswana [Vigna unguiculata]
B. yuanmingense B071T China [Lespedeza cuneata]
B. sp. R10m Botswana [Vigna unguiculata]
B. sp. 36-1-1 Namibia [Vigna subterranea]
B. liaoningense 2281T China [Glycine max]
B. sp. 15B (Nama-Karoo; WC)
VK-26
B. daqingense CCBAU15774T China [Glycine max]
B. americanum CMVU44T Venezuela [Centrosema macrocarpum]
B. huanghuaihaiense CCBAU23303T China [Glycine max]
B. genosp. β BRE-1 Spain [Teline canariensis]
B. sp. 26-1-1 Namibia [Arachis hypogaea]
B. neotropicale BR10247T Brazil [Centrolobium paraense]
B. centrolobii Br10245T Brazil [Centrolobium paraense]
B. spp. 30-1-1, 31-1-1, 32-2-1 & 30-2-1 All Namibia and [Lablab purpureus]; 21B-2-1 Namibia [Vigna subterranea]
B. iriomotense EK05T Japan [Entada koshunensis]
B. ingae BR10250T Brazil [Inga laurina]
B. rifense CTAW71T Morocco [Cytisus villosus]
B. ottawaense OO99T Canada [Glycine max]
B. sp. Arg105 South Africa [Argyrolobium rupestre]
B. arachidis CCBAU051107T China [Arachis hypogaea]
B. sp. CB756 Zimbabwe [Macrotyloma africanum]
B. stylosanthis BR446T Brazil [Stylosanthes guianensis]
B. centrosemae A9T Venezuela [Centrosema molle]
B. guangxiense CCBAU53363T China [Arachis hypogaea]
B. sp. RC3b South Africa [Vigna unguiculata]
B. kavangense 14-3T Namibia [Vigna unguiculata]
B. spp. 37-1-1 Namibia [Vigna subterranae]; 35 3-3, 35-3-4 & 34 1-1 All Namibia and [Vigna unguiculata]
B. manausense BR3351T Brazil [Vigna unguiculata]
B. ganzhouense RITF806T China [Acacia melanoxylon]
B. sp. CH2509 Brazil [Lupinus albescens]
B. genosp. α BC-C1 Canary Islands [Chamaecytisus proliferus]
B. spp. GHa, GHx Both Botswana and [Vigna unguiculata]
B. spp. 45-1-3 & 45-1-4 Both Angola and [Vigna unguiculata]
B. sp. 22-2-1 Namibia [Vigna unguiculata]
B. guangdongense CCBAU51649T China [Arachis hypogaea]
B. sp. F86-1-2 Mozambique [Faidherbia albida]
B. sp. F86-1-4 Mozambique [Faidherbia albida]
B. spp. Chvi; Ghiv; GC1d All Botswana and [Vigna unguiculata]
B. betae PL7HG1T Spain [Beta vulgaris tumors]
B. spp. LmiB2 & LmiB3 Both Tunisia and [Lupinus micranthus]
B. sp. LmiB4 Tunisia [Lupinus micranthus]
B. sp. LmicIA7 Algeria [Lupinus micranthus]
B. sp. LmicIA1 Algeria [Lupinus micranthus]
B. sp. LmicIA4 Algeria [Lupinus micranthus]
B. sp. LmicT3 Algeria [Lupinus micranthus]
B. sp. LmicT2 Algeria [Lupinus micranthus]
B. sp. MM5621 South Africa [Indigofera gracilis]
B. sp. LmicAM4 Algeria [Lupinus micranthus]
B. spp. LmiB16 & LmiB5 Both Tunisia and [Lupinus micranthus]
B. spp. LmicM10 & LmicAL52 Both Spain and [Lupinus micranthus]
B. sp. LmicM20 Spain [Lupinus micranthus]
B. sp. LmicAL54 Spain [Lupinus micranthus]
B. cytisi CTAW11T Morocco [Cytisus villosus]
B. sp. LmicT27 Algeria [Lupinus micranthus]
B. japonicum USDA6T Japan [Glycine max]
B. sp. BGA-1 Canary Islands [Teline stenopetala]
B. diazoefficiens USDA110T USA [Glycine max]
B. sp. LmicZ3 Algeria [Lupinus micranthus]
B. sp. CH2437 Bolivia [Lupinus tominensis]
B. sp. LmicAL48 Spain [Lupinus micranthus]
B. sp. LmicT8 Algeria [Lupinus micranthus]
B. sp. LmicT26 Algeria [Lupinus micranthus]
B. sp. LmicT18 Algeria [Lupinus micranthus]
B. sp. LmicT28 Algeria [Lupinus micranthus]
B. sp. LmicAM6 Algeria [Lupinus micranthus]
B. sp. LmicT5 Algeria [Lupinus micranthus]
B. sp. LmicT13 Algeria [Lupinus micranthus]
B. sp. LmicT16 Algeria [Lupinus micranthus]
B. sp. LmicAM3 Algeria [Lupinus micranthus]
B. sp. LmicAL27 Spain [Lupinus micranthus]
B. sp. LmicT10 Algeria [Lupinus micranthus]
B. sp. LmicZ10 Algeria [Lupinus micranthus]
B. sp. LmicF65 Spain [Lupinus micranthus]
B. sp. LmicAL42 Spain [Lupinus micranthus]
B. sp. LmicF61 Spain [Lupinus micranthus]
B. sp. LmicT14 Algeria [Lupinus micranthus]
B. sp. LmicZ4 Algeria [Lupinus micranthus]
B. canariense BTA-1T Canary Islands [Chamaecytisus proliferus]
B. sp. LmicAL32 Spain [Lupinus micranthus]
B. sp. LmicISLU122 Spain [Lupinus micranthus]
B. spp. LmicF63 & LmicAL38 Both Spain and [Lupinus micranthus]
B. spp. LmiT20, LmiT8 & LmiT9 All Tunisia and [Lupinus micranthus]
B. spp. ISLU16 Spain [Ornithopus compressus]; LmicM12 Spain [Lupinus micranthus]; LmiT14, LmiT13, LmiT12 & LmiM17 All Tunisia and [Lupinus micranthus]
B. sp. LmicISLU13 Spain [Lupinus micranthus]
B. spp. LmiT3 & LmiT1c Both Tunisia [Lupinus micranthus]
B. lupini USDA3051T New Zealand [Lupinus sp.]; B. spp. LmiT11, LmiT10 & LmiM15 All Tunisia and [Lupinus micranthus]
B. sp. LmiM11 Tunisia [Lupinus micranthus]
B. sp. LmiT6 Tunisia [Lupinus micranthus]
B. sp. BLUT1 Canary Islands [Lupinus albus]
B. spp. LmiT7 & LmiT15 Both Tunisia and [Lupinus micranthus]
B. denitrificans LMG8443T Germany [Surface water]
B. oligotrophicum LMG10732T Japan [Rice paddy soil]
B. sp. Leo20 South Africa [Leobordea pulchra]
B. spp. Leo166 South Africa [Leobordea pulchra]; Leo170 South Africa [Leobordea lanceolata]
B. sp. 26-3-1 Namibia [Arachis hypogaea]
B. sp. 18C-2-1 Namibia [Vigna unguiculata]
B. sp. WSM2632 South Africa [Lotononis sp.]
B. sp. WSM2783 South Africa [Leobordea carinata]
B. spp. Leo78, Leo132 & Leo142 All South Africa and [Leobordea pulchra]
B. spp. Leo114 & Leo121 Both South Africa and [Leobordea divaricata]
B. sp. Leo117 South Africa [Leobordea divaricata]
B. sp. Leo79 South Africa [Leobordea pulchra]
B. sp. Leo84 South Africa [Leobordea pulchra]
B. spp. F83-5-1 & F85-3-3 Both Mozambique and [Faidherbia albida]
B. sp. F85-2-1 Mozambique [Faidherbia albida]
B. spp. F83-1-3 & F86-5-2 Both Mozambique and [Faidherbia albida]
B. spp. F83-1-1 & F86-4-2 Both Mozambique and [Faidherbia albida]
B. sp. F85-3-4 Mozambique [Faidherbia albida]
B. spp. F85-1-4, F86-2-3, F85-4-3 & F86-3-3 All Mozambique and [Faidherbia albida]
B. brasilense UFLA03T Brazil [Vigna unguiculata]
B. spp. F82-4-1, F82-1-1 & F82-4-3 All Mozambique and [Faidherbia albida]
B. pachyrhizi PAC48T Costa Rica [Pachyrhizus erosus]
B. sp. Arg68 South Africa [Argyrolobium sericeum]
B. macuxiense BR10303T Brazil [Centrolobium paraense]
B. viridifuturi SEMIA690T Brazil [Centrosema pubescens]
B. sp. P14-1-1 Angola [Vigna subterranea]
B. tropiciagri SEMIA6148T Brazil [Neonotonia wightii]
B. erythrophlei CCBAU53325T China [Erythrophleum fordii]
B. sp. 49-2-1 Angola [Phaseolus vulgaris]
B. spp. F1-1-1 Angola [Vigna subterranea]; 46-1-1 Angola [Vigna unguiculata]
B. spp. 47-1-1 & 51-1-3 Both Angola and [Vigna unguiculata]; F1-2-1 Angola [Vigna subterranea]
B. sp. 44-1-1 Angola [Vigna unguiculata]
B. sp. 40-3-1 & 40-2-2 Both Angola and [Vigna unguiculata]; 42-1-1 Angola [Phaseolus vulgaris]
B. sp. 41-1-2 Angola [Vigna unguiculata]
B. sp. WSM2596 South Africa [Leobordea foliosa]
B. sp. Pear77 South Africa [Pearsonia obovata]
B. sp. MM5392 South Africa [Indigofera frutescens]
B. embrapense SEMIA6208T Colombia [Desmodium heterocarpon]
B. sp. MM5405 South Africa [Tephrosia capensis]
B. sp. LmicA16 Algeria [Lupinus micranthus]
B. sp. Leo176 South Africa [Leobordea lanceolata]
B. sp. Pear76 South Africa [Pearsonia obovata]
B. spp. Pear129 & Pear128 Both South Africa and [Pearsonia obovata]
B. spp. F85-2-3 & F83-2-3 Both Mozambique and [Faidherbia albida]
B. elkanii USDA76T Japan [Glycine max]
B. sp. F83-2-1 Mozambique [Faidherbia albida]
B. ferriligni CCBAU51502T China [Erythropleum fordii]
B. sp. Alb4-1-4 Mozambique [Albizia versicolor]
B. sp. Alb4-1-6 Mozambique [Albizia versicolor]
B. spp. Alb2-B-4 & Alb2-B-2 Both Mozambique and [Albizia versicolor]
B. sp. Alb3-4-4 Mozambique [Albizia versicolor]
B. spp. Alb1-C-2 Mozambique [Albizia versicolor]
B. sp. Alb3-2-1 Mozambique [Albizia versicolor]
B. sp. Alb3-4-5 Mozambique [Albizia versicolor]
B. sp. Alb4-1-5 Mozambique [Albizia versicolor]
B. sp. Alb3-4-1 Mozambique [Albizia versicolor]
B. sp. Arg62 South Africa [Argyrolobium sericium]
B. sp. AD1T2-3-1 Angola [Vigna subterranea]
B. spp. Alb5-2-6, Alb4-1-3; Alb1-B-2, Alb4-1-2, Alb3-1-5, Alb3-3-3 & Alb3-3-4 All Mozambique and [Albizia versicolor]
B. sp. R5 Botswana [Vigna unguiculata]
B. spp. Cham227 & Cham231 Both South Africa and [Chamaecrista sp.]
B. spp. LmiH2 & LmiH3 Both Tunisia and [Lupinus micranthus]
B. valentinum LmjM3T Sapain [Lupinus mariae-josephae]
B. spp. LmiM2, LmiM3 & LmiM1 All Tunisia and [Lupinus micranthus]
B. icense LMTR13T Peru [Phaseolus lunatus]
B. lablabi CCBAU23086T China [Lablab purpureus]
B. sp. 18C-1-1 Namibia [Vigna unguiculata]
B. retamae Ro19T Morocco [Retama monosperma]
B. sp. LmiH4 Tunisia [Lupinus micranthus]
B. jicamae PAC68T Honduras [Pachyrhizus erosus]
B. paxllaeri LMTR21T Peru [Phaseolus lunatus]
B. spp. 30-3-3 & 30 3-2 Both Namibia and [Lablab purpureus]
B. spp. 5-11 & 5-10 Both Namibia and [Lablab purpureus]
Rhodopseudomonas palustris DX-1 Rpdx1_1498
Rhodopseudomonas palustris CGA009 RPA3851
Rhodopseudomonas palustris TIE-1 Rpa1_4376
0.05

Suppl. Fig. S5 A *recA* maximum-likelihood phylogeny of the genus *Bradyrhizobium*. The single isolate recovered during this study is indicated in red, together with the information for which biome and province (abbreviated as in Table 1) the soil for the 'trapping' originated from. The lineage to which this isolate belongs (VK-26) appears in blue. *Bradyrhizobium* type strains are in bold, and all isolates include information for their country of origin and host or source. GenBank accession numbers and associated references for the included isolates are listed in Suppl. Table S5. Three *Rhodopseudomonas* isolates were used for outgroup purposes. Only bootstrap support of ≥ 50% is indicated and the scale bar indicates the number of nucleotide changes per site.
